# In vivo functional profiling and structural characterization of the human *GLP1R* A316T variant

**DOI:** 10.1126/sciadv.adw0899

**Published:** 2026-02-04

**Authors:** Liliane El Eid, Yusman Manchanda, Gregory Austin, Kieran Deane-Alder, Roxana-Maria Rujan, Zamara Mariam, Affiong I. Oqua, Matthew J. Belousoff, Jorge Bernardino de la Serna, Kyle W. Sloop, Guy A. Rutter, Alex Montoya, Dominic J. Withers, Steven J. Millership, Karim Bouzakri, Ben Jones, Christopher A. Reynolds, Patrick M. Sexton, Denise Wootten, Giuseppe Deganutti, Alejandra Tomas

**Affiliations:** ^1^ Section of Cell Biology and Functional Genomics, Division of Diabetes, Endocrinology and Metabolism, Department of Metabolism, Digestion and Reproduction, Imperial College London, London, UK.; ^2^ Drug Discovery Biology, Monash Institute of Pharmaceutical Sciences, Monash University, Parkville, Victoria, Australia.; ^3^ ARC Centre for Cryo-Electron Microscopy of Membrane Proteins, Monash Institute of Pharmaceutical Sciences, Monash University, Parkville, Victoria, Australia.; ^4^ Centre for Health and Life Sciences, Coventry University, Coventry, UK.; ^5^ National Heart and Lung Institute, Imperial College London, London, UK.; ^6^ Diabetes, Obesity and Complications, Lilly Research Laboratories, Eli Lilly and Company, Indianapolis, IN, USA.; ^7^ CHUM Research Centre, Faculty of Medicine, University of Montreal, Montreal, QC, Canada.; ^8^ Research Institute of the McGill University Health Centre, Montreal, QC, Canada.; ^9^ LKC School of Medicine, Nanyang Technological University, Singapore.; ^10^ MRC Laboratory of Medical Sciences, London, UK.; ^11^ Institute of Clinical Sciences, Faculty of Medicine, Imperial College London, London, UK.; ^12^ Centre Européen d’Étude du Diabète, Strasbourg, France.; ^13^ Section of Endocrinology and Investigative Medicine, Division of Diabetes, Endocrinology and Metabolism, Department of Metabolism, Digestion and Reproduction, Imperial College London, London, UK.; ^14^ School of Life Sciences, University of Essex, Colchester, UK.

## Abstract

Glucagon-like peptide-1 receptor agonists (GLP-1RAs) are effective therapies for type 2 diabetes (T2D) and obesity, yet patient responses are variable, with *GLP1R* gene variation potentially linked to therapeutic outcomes. A *GLP1R* natural missense variant, A316T, protects against T2D and cardiovascular disease. Here, we generated and characterized a human *GLP1R* A316T mouse model. Human *GLP1R*^A316T/A316T^ mice displayed lower fasting blood glucose versus wild-type littermates even under metabolic stress, as well as slower weight gain and alterations in islet cytoarchitecture, glucagon secretion, and liver metabolism under a high-fat, high-sucrose diet. This was however associated with blunted responses to pharmacological GLP-1RAs in vivo. Further investigations in β cell models demonstrated that human *GLP1R* A316T exhibits characteristics of constitutive activation but dampened GLP-1RA responses. Results are further supported by cryo-EM analyses and molecular dynamics simulations of GLP-1R A316T structure, collectively demonstrating that the A316T variant governs basal GLP-1R activity and pharmacological responses to GLP-1R–targeting therapies.

## Teaser

The *GLP1R* A316T missense variant displays improved glucose tolerance but impaired pharmacological incretin responses in vivo.

## INTRODUCTION

The glucagon-like peptide-1 receptor (GLP-1R), an important drug target for the treatment of type 2 diabetes (T2D), obesity, and cardiovascular disease, is a class B1 secretin-like G protein–coupled receptor (GPCR) predominantly expressed in the pancreas but also present in other organs such as the brain, heart, stomach, intestine, and kidneys (*1*–*3*). The endogenous ligand for GLP-1R, GLP-1, is a 30– or 31–amino acid peptide incretin hormone secreted from intestinal L cells upon food intake (*4*), unsuitable for pharmacological use due to its short half-life (~2 min). Several pharmacological GLP-1 analogs, developed to bypass rapid degradation by dipeptidyl peptidase 4 (DPP-4) and, in conjunction with lipid modifications to reduce renal elimination, prolong pharmacokinetics (*5*), have been proven highly efficacious in controlling blood glucose levels and reducing body weight (*6*, *7*).

Akin to other GPCRs, the GLP-1R undergoes a conformational change upon agonist binding, pleiotropically coupling with heterotrimeric G proteins (*8*, *9*), with preferential engagement of Gα_s_, leading to activation of adenylate cyclase, generation of cyclic adenosine monophosphate (cAMP), potentiation of intracellular calcium rises, insulin granule mobilization, and exocytosis in pancreatic β cells (*4*, *10*). The GLP-1R can additionally inhibit glucagon secretion, delay gastric emptying, and suppress appetite (*4*, *11*), with further physiological functions, including blood pressure regulation (*12*, *13*), maintenance of renal function (*14*, *15*), and neuroprotection (*16*–*19*), as well as beneficial effects in metabolic dysfunction–associated steatotic liver disease (MASLD) (*20*) revealed by recent studies.

Despite the clinical success of GLP-1R agonists (GLP-1RAs), there is considerable heterogeneity among patient responses, with some exhibiting marked therapeutic improvements while others show little to no beneficial effect and/or prominent adverse side effects (*21*). Natural genetic variants of human GPCRs can be linked to changes in receptor function, e.g., alterations in receptor structure, basal and ligand-dependent activity, and/or level of expression (*22*). Several *GLP1R* missense variants have been identified, some of which are associated with changes in T2D risk and related glycemic traits in genome-wide association studies (GWAS) (*23*–*26*). Large-scale sequencing and functional genetics have revealed a *GLP1R* missense variant (A316T; rs10305492; minor allele frequency = 1.4%), associated with lower fasting glucose and reduced T2D and cardiovascular disease risk (*23*–*25*). The consequences of this variant on receptor function remain poorly defined, with conflicting results reported (*27*, *28*). Previous analyses of variant receptor signaling in vitro suggest that it confers a gain-of-function (GoF) phenotype (*25*, *29*, *30*), but in-depth characterization in primary tissues and in vivo has not been performed to date.

Here, we present a detailed comparison between wild-type (WT) and A316T GLP-1R, including functional characterization in pancreatic β cell lines and primary murine and human islets, as well as in vivo analysis of A316T variant effects using a newly generated mouse model bearing a homozygous A316T (c.946G>A) substitution in the human *GLP1R* gene (*hGLP1R*^A316T/A316T^), both under physiological conditions and following diabetes induction by prolonged feeding with a high-fat, high-sucrose (HFHS) diet. We additionally present the cryo–electron microscopy (cryo-EM) structure of the A316T GLP-1R bound to GLP-1, together with computational analyses based on molecular dynamics (MD) simulations of WT versus A316T receptor, which elucidate the structural changes that underlie the functional effects of the A316T variant.

## RESULTS

### Human *GLP1R*^A316T/A316T^ mice exhibit enhanced glucose homeostasis but reduced pharmacological responses to GLP-1RAs under different nutritional states

Previous GWAS results have led to the hypothesis that the A316T variant constitutively activates the GLP-1R, causing increased insulin secretion at lower ambient glucose levels, which in turn might trigger receptor down-regulation over time, potentially resulting in incretin resistance (*31*). To test this hypothesis in vivo, a mouse line expressing human GLP-1R from the murine *Glp1r* locus (*32*) was used to generate a global knock-in model harboring the A316T single-nucleotide polymorphism (SNP) (*hGLP1R*^A316T/A316T^), with WT *hGLP1R*^+/+^ littermates used as controls (fig. S1, A and B). Mouse genotypes were confirmed by Sanger sequencing, and glucose homeostasis was analyzed by crossover studies in a mixed sex cohort.

**Fig. 1.**
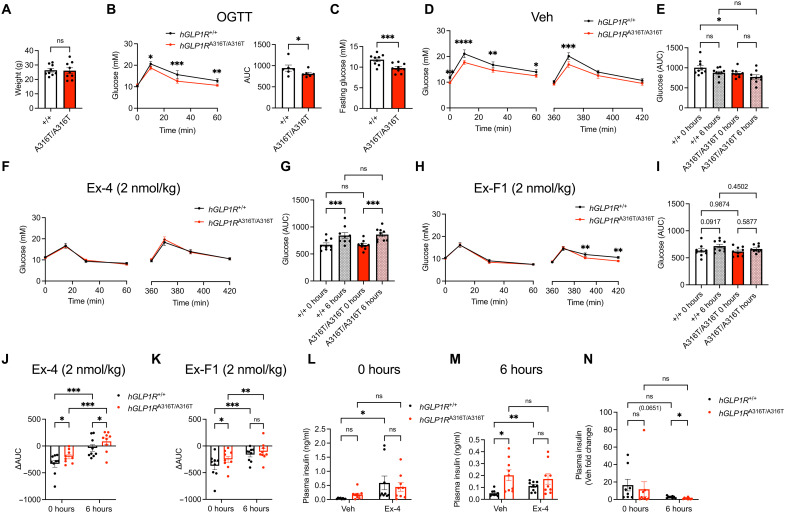
In vivo glucose responses of adult *hGLP1R*^A316T/A316T^ versus *hGLP1R*^+/+^ mice on a chow diet. (**A**) Age-matched body weight of *hGLP1R*^+/+^ versus ^A316T/A316T^ mixed sex adult littermate mice (*n* = 9 per genotype) maintained on a normal chow diet. (**B**) OGTT responses and corresponding AUCs following 2 g/kg body weight glucose administration via oral gavage after 5 hours of fasting in a mixed sex cohort of lean, adult *hGLP1R*^+/+^ versus ^A316T/A316T^ mice on a chow diet (*n* = 6 per genotype). (**C**) Glucose levels after 2 hours of fasting from mice in (A). (**D** and **E**) IPGTTs (2 g/kg body weight glucose) following acute (0 hours) or 6 hours postintraperitoneal administration of saline vehicle (Veh) in chow-fed *hGLP1R*^+/+^ versus ^A316T/A316T^ mixed sex adult mice. Glucose curves (D) and corresponding AUCs (E) shown; *n* = 9 mice per genotype. (**F** to **I**) As in (D) and (E) following administration of exendin-4 (Ex-4; 2 nmol/kg) [(F) and (G)] or exendin-F1 (Ex-F1) [(H) and (I)]. (**J** and **K**) Vehicle-corrected ΔAUC responses from (G) and (I), respectively. (**L** and **M**) Plasma insulin levels acutely (L) and 6 hours (M) postadministration of Veh or Ex-4 in chow-fed *hGLP1R*^+/+^ versus ^A316T/A316T^ mixed sex adult mice. (**N**) Plasma insulin vehicle fold changes from (L) and (M). Data are mean ± SEM; *P < 0.05, **P < 0.01, ***P < 0.001, and ****P < 0.0001; ns, not significant; by paired *t* tests, one-, or two-way analysis of variance (ANOVA) with Sidak’s post hoc tests.

Despite no discernible differences in weight or peripheral insulin sensitivity between *hGLP1R*^A316T/A316T^ and WT littermates on a chow diet (Fig. 1A and fig. S2A), and a nonsignificant tendency for reduced plasma GLP-1 in response to oral glucose in *hGLP1R*^A316T/A316T^ mice (fig. S2B), glucose tolerance was improved following an oral gavage (Fig. 1B). In addition, fasting blood glucose was significantly lower in *hGLP1R*^A316T/A316T^ mice (Fig. 1C), in agreement with previous GWAS data (*23*–*25*). To examine the acute versus sustained pharmacogenetic effects of the GLP-1R A316T variant, we performed intraperitoneal glucose tolerance tests (IPGTTs) immediately after (“acute”) and 6 hours postadministration (“sustained”) of vehicle or the GLP-1RAs exendin-4 or exendin-F1, which have differing biased agonism profiles when assessed in vitro at the WT GLP-1R (*33*). We observed significantly improved acute glucose responses to vehicle administration in *hGLP1R*^A316T/A316T^ versus *hGLP1R*^+/+^ littermates, with a similar trend at the 6-hour time point (Fig. 1, D and E). Conversely, *hGLP1R*^A316T/A316T^ and *hGLP1R*^+/+^ mice exhibited similar glucose responses to exendin-4 both acutely and 6 hours postagonist administration (Fig. 1, F and G), with comparably attenuated glucose effects at the 6-hour time point, associated with exendin-4–induced receptor desensitization (*33*). A similar trend was observed with exendin-F1, which, as expected by its bias profile away from β-arrestin recruitment, displayed reduced desensitization at the 6-hour IPGTT for both mouse genotypes versus exendin-4 (*33*), with prolonged glucose levels slightly improved for *hGLP1R*^A316T/A316T^ mice (Fig. 1H), without reaching statistical significance when measured as area under the curve (AUC) versus *hGLP1R*^+/+^ mice (Fig. 1I). Direct comparison between AUCs for the different conditions and genotypes is shown in fig. S2 (C and D). Agonist-specific glycemic responses (ΔAUC, corresponding to agonist- minus vehicle-treated AUC, measurable due to the crossover design of the study) revealed significantly reduced acute exendin-4 responses in *hGLP1R*^A316T/A316T^ versus *hGLP1R*^+/+^ littermates, a difference that was maintained at the 6-hour time point, when exendin-4 treatment resulted in a paradoxical increase in blood glucose levels compared to vehicle in *hGLP1R*^A316T/A316T^ mice (Fig. 1J). While *hGLP1R*^A316T/A316T^ mice displayed similarly reduced acute responses to exendin-F1 when compared to *hGLP1R*^+/+^ mice, this difference was absent after 6 hours, suggesting a beneficial impact of the reduced desensitization afforded by this agonist (*33*) in mice expressing the A316T variant (Fig. 1K). These effects were also apparent when calculated as vehicle fold changes (fig. S2E). No significant differences were detected per genotype at prolonged minus acute time points (fig. S2, F and G), indicating that the loss of effect in response to GLP-1RAs is already present acutely. Similar experiments in heterozygous *hGLP1R*^A316T/+^ mice showed no clear differences versus WT *hGLP1R*^+/+^ littermates under a chow diet (fig. S3, A to K).

Plasma samples collected 10 min into the IPGTTs showed higher insulin levels in *hGLP1R*^A316T/A316T^ versus *hGLP1R*^+/+^ littermates after vehicle administration, an effect that became significant for the 6-hour time point (Fig. 1, L to M). As expected, plasma insulin levels increased in response to exendin-4 for the *hGLP1R*^+/+^ mice, but this effect was attenuated in *hGLP1R*^A316T/A316T^. When expressed as vehicle fold changes, exendin-4–dependent plasma insulin rises were reduced for *hGLP1R*^A316T/A316T^ versus *hGLP1R*^+/+^ littermates, a difference that became significant at the 6-hour time point (Fig. 1N).

**Fig. 2.**
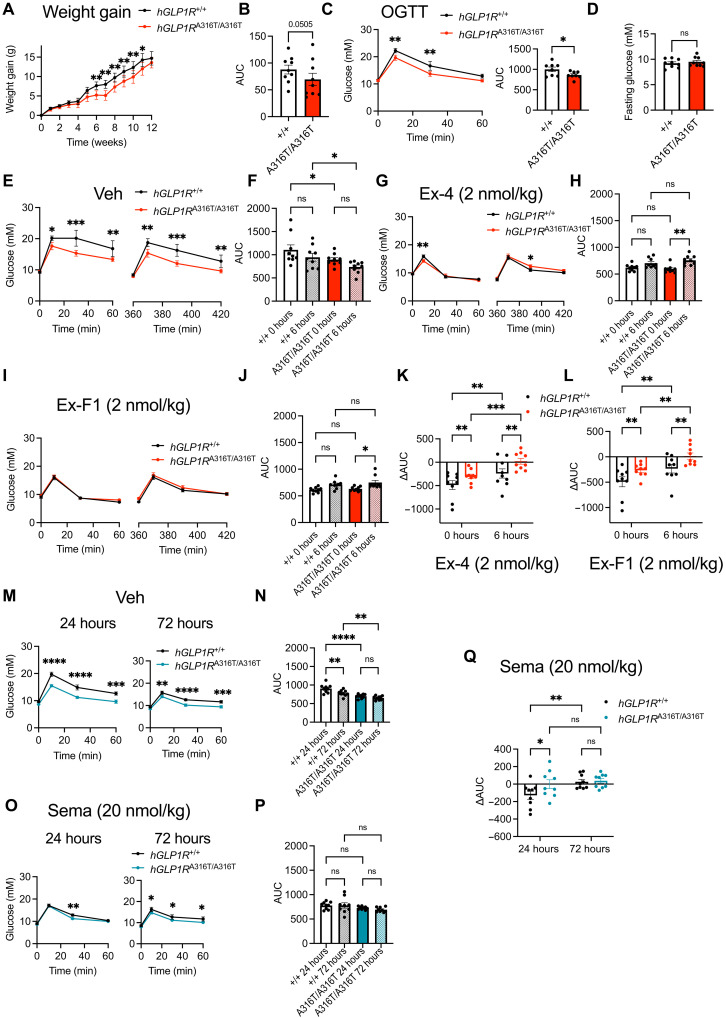
In vivo glucose responses of adult *hGLP1R*^A316T/A316T^ versus *hGLP1R*^+/+^ mice on a HFHS diet. (**A**) Body weight gain for *hGLP1R*^+/+^ versus ^A316T/A316T^ mixed sex adult littermate mice (*n* = 9 per genotype) maintained on a HFHS diet for the indicated times. (**B**) AUCs from (A). (**C**) OGTT responses and corresponding AUCs following 2 g/kg body weight glucose administration via oral gavage after 5 hours of fasting in a mixed sex cohort of adult *hGLP1R*^+/+^ versus ^A316T/A316T^ mice on a HFHS diet (*n* = 8 per genotype). (**D**) Glucose levels after 2 hours of fasting in mice from (A). (**E** and **F**) IPGTTs (2 g/kg body weight glucose) following acute (0 hours) or 6 hours postintraperitoneal administration of saline vehicle (Veh) in HFHS-fed *hGLP1R*^+/+^ versus ^A316T/A316T^ mixed sex adult mice. Glucose curves (E) and corresponding AUCs (F) shown; *n* = 9 mice per genotype. (**G** to **J**) As in (E) and (F) following administration of exendin-4 (Ex-4; 2 nmol/kg) [(G) and (H)] or exendin-F1 (Ex-F1) [(I) and (J)]. (**K** and **L**) Vehicle-corrected ΔAUC responses from (H) and (J), respectively. (**M** to **P**) Glucose curves and corresponding AUCs following vehicle (Veh) [(M) and (N)] or semaglutide (Sema; 20 nmol/kg) [(O) and (P)] treatment of HFHS-fed *hGLP1R*^+/+^ versus ^A316T/A316T^ mice; IPGTTs performed 24 and 72 hours postagonist administration. (**Q**) Vehicle-corrected ΔAUC responses from (P). Data are mean ± SEM; *P < 0.05, **P < 0.01, ***P < 0.001, and ****P < 0.0001; by paired *t* tests, one-, or two-way ANOVA with Sidak’s post hoc tests.

We next subjected our mouse cohort to 12 to 14 weeks of HFHS diet feeding to investigate variant-associated phenotypes under diet-induced metabolic stress. During the HFHS feeding period, *hGLP1R*^A316T/A316T^ mice displayed slower weight gain compared to *hGLP1R*^+/+^ littermates (Fig. 2, A and B) but still reached comparable weights at the end of the HFHS feeding period, allowing us to assess glucoregulatory effects without confounding differences in body weight. Consistently with results in lean conditions, oral glucose tolerance was significantly improved in *hGLP1R*^A316T/A316T^ versus *hGLP1R*^+/+^ mice (Fig. 2C), with no changes observed in fasting glycemia (Fig. 2D) but a nonsignificant tendency for improved peripheral insulin sensitivity measured in *hGLP1R*^A316T/A316T^ mice by an intraperitoneal insulin tolerance test (IPITT) (fig. S4A). HFHS-fed *hGLP1R*^A316T/A316T^ mice also exhibited significantly enhanced intraperitoneal glucose tolerance under vehicle conditions compared to *hGLP1R*^+/+^ mice, both acutely and at the 6-hour time point (Fig. 2, E and F). Glucose responses to exendin-4 and exendin-F1 resembled those of *hGLP1R*^+/+^ mice but with slightly more pronounced desensitization for *hGLP1R*^A316T/A316T^ mice (Fig. 2, G to J). Direct comparison between AUCs for the different conditions and genotypes is shown in fig. S4 (B and C). When calculated as vehicle ΔAUC, the glucose-lowering effect of both GLP-1RAs was significantly reduced in HFHS-fed *hGLP1R*^A316T/A316T^ versus *hGLP1R*^+/+^ littermates, with differences apparent both acutely and 6 hours postadministration of either agonist (Fig. 2, K and L). Effects were also apparent when calculated as vehicle fold changes (fig. S4D), with no significant differences in prolonged minus acute effect per agonist and genotype, indicating that the loss of GLP-1RA responses in *hGLP1R*^A316T/A316T^ mice is already present acutely (fig. S4, E and F). Basal plasma insulin levels were once more increased in HFHS-fed *hGLP1R*^A316T/A316T^ versus *hGLP1R*^+/+^ mice but now significantly also at the acute time point (fig. S4G).

To assess the effects of the A316T variant in response to a GLP-1RA with prolonged pharmacokinetics, we conducted additional experiments in HFHS-fed animals with the long-acting GLP-1RA semaglutide (*34*). We observed a similar profile of enhanced glucose tolerance under vehicle conditions at 24 and 72 hours in HFHS-fed *hGLP1R*^A316T/A316T^ versus *hGLP1R*^+/+^ littermates (Fig. 2, M and N). Glucose responses to semaglutide were very similar to those of *hGLP1R*^+/+^ littermates, with slightly lower glucose levels across the time course for *hGLP1R*^A316T/A316T^ but no significant changes in AUC (Fig. 2, O and P). Direct comparison between AUCs is shown in fig. S4 (H to I). Results calculated as ΔAUC showed that semaglutide induces lower responses in *hGLP1R*^A316T/A316T^ versus *hGLP1R*^+/+^ littermates 24 hours postagonist treatment, with these differences no longer apparent at the 72-hour time point, when both genotypes display fully desensitized responses (Fig. 2Q). These effects were similarly apparent when calculated as vehicle fold changes (fig. S4J). Consequently, we observed a near-significant reduction in prolonged minus acute effects for semaglutide in *hGLP1R*^A316T/A316T^ compared to *hGLP1R*^+/+^ mice (fig. S4K), indicating that the loss of semaglutide response in *hGLP1R*^A316T/A316T^ mice occurs acutely, potentially due to accelerated receptor desensitization. Despite heterozygous *hGLP1R*^A316T/+^ mice showing more attenuated differences versus *hGLP1R*^+/+^ littermates for weight gain, oral glucose tolerance, fasting glucose, and acute versus 6-hour IPGTT responses under a HFHS diet (fig. S5, A to L), these mice exhibited significantly increased desensitization 6 hours post–exendin-4 (fig. S5, G and H), as well as improved glucose tolerance 24 hours postvehicle administration (fig. S5, M and N), the latter leading to significantly worse ΔAUC responses to semaglutide versus *hGLP1R*^+/+^ littermates (fig. S5, O to Q), an effect that, as for exendin-4 administration in chow-fed homozygous mice, resulted in paradoxically higher glucose levels versus those of vehicle-treated *hGLP1R*^A316T/+^ mice (fig. S5Q).

Taken as a whole, these results indicate that the A316T variant displays improved in vivo glucose tolerance under vehicle conditions, both in response to oral glucose ingestion and to intraperitoneal glucose administration but is associated with pharmacological incretin resistance linked to increased desensitization propensity of the variant receptor.

### Alterations of islet cytoarchitecture and α/β cell identity in human *GLP1R*^A316T/A316T^ mice

**Fig. 3.**
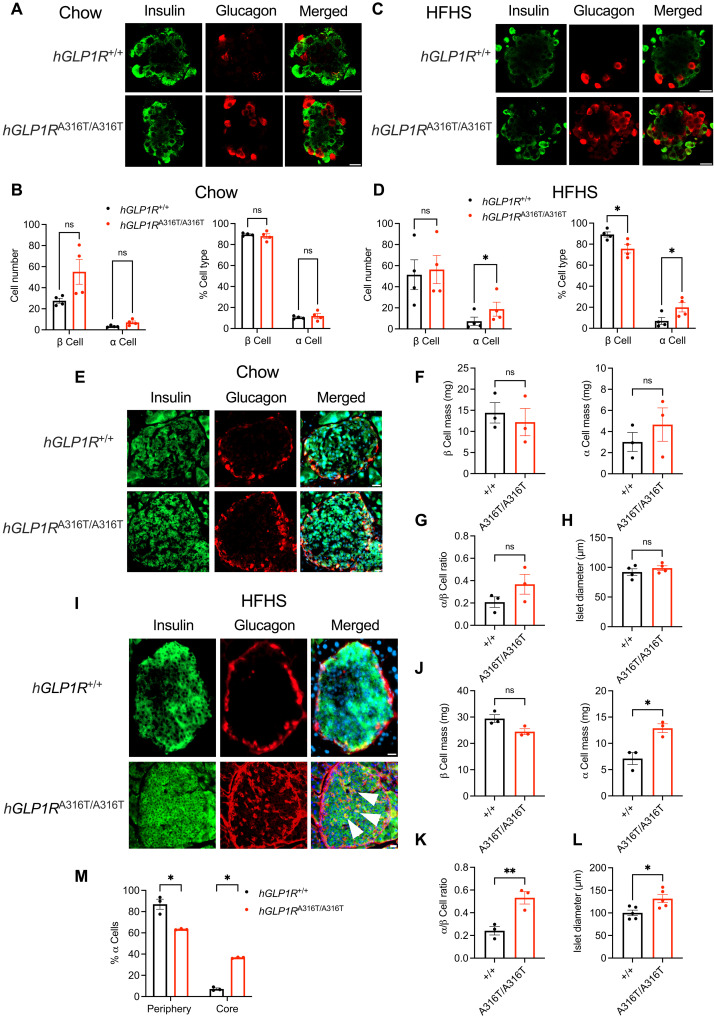
Morphological analyses of *hGLP1R*^A316T/A316T^ versus *hGLP1R*^+/+^ mouse islets. (**A** and **B**) Immunofluorescence analysis of insulin (green) and glucagon (red) localization in isolated islets from chow-fed *hGLP1R*^+/+^ versus ^A316T/A316T^ mice; scale bars, 20 μm. Representative images (A) and α versus β cell number and percentage quantifications (B); *n* = 4. (**C** and **D**) As in (A) and (B) for islets from HFHS-fed *hGLP1R*^+/+^ versus ^A316T/A316T^ mice; *n* = 4. (**E** to **H**) Immunofluorescence analysis of insulin (green) and glucagon (red) localization in pancreas sections from chow-fed *hGLP1R*^+/+^ versus ^A316T/A316T^ mice; nuclei [4′,6-diamidino-2-phenylindole (DAPI)], blue; scale bars, 20 μm. Representative images (E), quantification of α and β cell number (F), α over β cell ratio (G), and islet diameter (H); *n* = 3 to 4. (**I** to **L**) As for (E) to (H) in pancreas sections from HFHS-fed *hGLP1R*^+/+^ versus ^A316T/A316T^ mice. Representative images (I), quantification of α and β cell number (J), α over β cell ratio (K), and islet diameter (L); *n* = 3 to 5; scale bars, 20 μm; arrowheads indicate cells positive for both insulin and glucagon. (**M**) Percentage of islet peripheral/mantle versus core α cells in pancreas sections from HFHS-fed *hGLP1R*^+/+^ versus ^A316T/A316T^ mice; *n* = 3. Data are mean ± SEM; *P < 0.05 and **P < 0.01; by paired *t* tests or two-way ANOVA with Sidak’s post hoc tests.

Previous studies suggest that, because of its proliferative and antiapoptotic effects, prolonged GLP-1R activity might lead to alterations in critical aspects of islet cytoarchitecture such as size, cellular composition, and α/β cell identity (*35*–*38*). Changes in islet cytoarchitecture have also been linked to β cell dysfunction and T2D progression (*39*–*42*). With the A316T variant conferring a GoF phenotype in both oral glucose tolerance and intraperitoneal glucose responses, indicative of increased basal GLP-1R activity, we hypothesized that it might also lead to changes in islet cytoarchitecture and α and/or β cell identity, a possibility that has not been assessed in previous in vitro analyses of this variant. To test this, we conducted histological examinations of pancreatic tissue and islets isolated from chow- and HFHS-fed *hGLP1R*^A316T/A316T^ versus *hGLP1R*^+/+^ mice. Chow-fed *hGLP1R*^A316T/A316T^ islets showed a nonsignificant tendency for increased numbers of both insulin- and glucagon-positive cells, without changes in percentages (Fig. 3, A and B). HFHS-fed *hGLP1R*^A316T/A316T^ islets, however, displayed a remarkable increase in the percentage of glucagon-positive cells, and a concomitant decrease in insulin-positive cells compared to *hGLP1R*^+/+^ islets (Fig. 3, C and D). Results were recapitulated in whole pancreas sections: while chow-fed mice did not show significant differences in β or α cell mass, α-to-β cell ratio, or islet diameter between genotypes (Fig. 3, E to H), immunolabeling of pancreas sections from HFHS-fed *hGLP1R*^A316T/A316T^ mice revealed increased α cell mass, α-to-β cell ratio, and islet diameter versus *hGLP1R*^+/+^ (Fig. 3, I to L). α Cells from HFHS-fed *hGLP1R*^A316T/A316T^ islets also showed a distinct loss of localization to the islet periphery (the two outermost islet cell layers) and were instead intermingled with β cells throughout the islet core (Fig. 3M). Closer examination of intra-islet endocrine cell localization revealed a significant proportion of nonperipheral cells expressing both insulin and glucagon (Fig. 3I, arrowheads), without signs of increased proliferation within this cell pool at the 12-week HFHS diet time point (fig. S6A), suggesting earlier α cell expansion. Analysis of expression of β cell “enriched” and “disallowed” genes revealed up-regulation of *Mafa*, a gene associated with β cell identity, in islets from both chow- and HFHS-fed *hGLP1R*^A316T/A316T^ mice, with no changes in β cell disallowed gene expression (*Slc16a1* and *Ldha*), and only a tendency for increased expression of α cell–specific *Gcg* (fig. S6, B and C). Expression of the β cell–disallowed gene *Acot7* (*43*) was significantly up-regulated in chow-fed *hGLP1R*^A316T/A316T^ islets.

### Altered glucagon secretion and liver metabolism in HFHS-fed human *GLP1R*^A316T/A316T^ mice

**Fig. 4.**
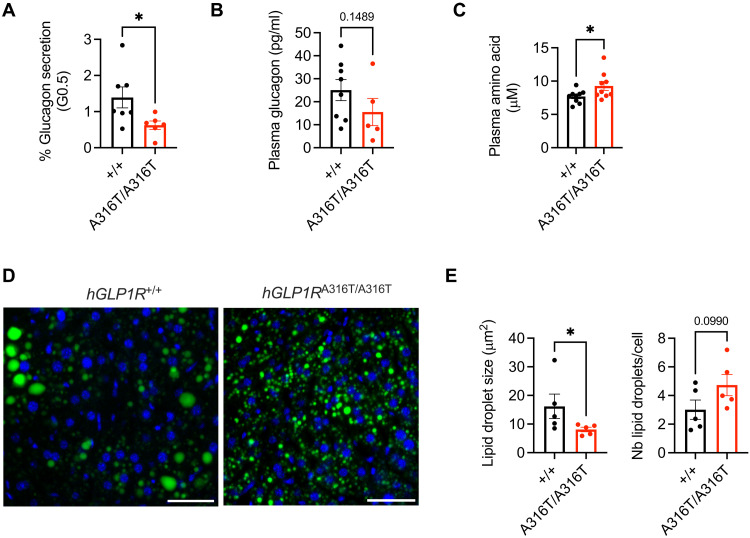
Glucagon and liver responses from *hGLP1R*^A316T/A316T^ versus *hGLP1R*^+/+^ mice. (**A**) Percentage of glucagon secretion in HFHS-fed *hGLP1R*^+/+^ versus ^A316T/A316T^ mouse islets at 0.5 mM glucose (G0.5); *n* = 6 for *hGLP1R*^A316T/A316T^ and *n* = 7 for *hGLP1R*^+/+^. (**B**) Plasma glucagon levels in HFHS-fed *hGLP1R*^+/+^ versus ^A316T/A316T^ mixed sex adult mice following 5-hour fasting; *n* = 5 for *hGLP1R*^A316T/A316T^ and *n* = 7 for *hGLP1R*^+/+^. (**C**) Plasma amino acid levels in HFHS-fed *hGLP1R*^+/+^ versus ^A316T/A316T^ mixed sex adult mice; *n* = 9 mice per genotype. (**D** and **E**) Lipid droplet labeling in liver sections from HFHS-fed *hGLP1R*^+/+^ versus ^A316T/A316T^ mice; representative images (D), quantification of average lipid droplet size, and number of lipid droplets per cell (E); *n* = 5; lipid droplets (BODIPY), green; nuclei (DAPI), blue; scale bars, 50 μm. Data are mean ± SEM; *P < 0.05; by unpaired *t* tests.

Given the observed increase in α cell mass in *hGLP1R*^A316T/A316T^ islets from HFHS-fed mice, and the known inhibitory effect of GLP-1R action on glucagon secretion (*44*), we next analyzed glucagon outputs from islets extracted from *hGLP1R*^+/+^ versus *hGLP1R*^A316T/A316T^ mice fed a HFHS diet for 10 weeks. Glucagon secretion at a permissive low glucose concentration (0.5 mM) was significantly decreased in *hGLP1R*^A316T/A316T^ islets (Fig. 4A) despite the increased number of glucagon-expressing cells versus *hGLP1R*^+/+^ islets. Accordingly, we also measured a tendency toward decreased fasting plasma glucagon levels in HFHS-fed *hGLP1R*^A316T/A316T^ compared to control *hGLP1R*^+/+^ mice (Fig. 4B). The development of α cell hyperplasia is closely linked to the disruption of the liver–α cell axis, with defective liver glucagon signaling leading to reduced amino acid metabolism resulting in plasma hyperaminoacidemia, with high levels of plasma amino acids subsequently promoting α cell expansion in a feedback loop attempt to restore glucagon secretion (*45*, *46*). Consistently, we measured a significant increase in plasma amino acid levels in HFHS-fed *hGLP1R*^A316T/A316T^ compared with *hGLP1R*^+/+^ mice (Fig. 4C). As both indirect GLP-1R action and direct glucagon signaling in the liver are linked to reduced liver steatosis (*47*–*49*), we next examined the degree of lipid accumulation in liver sections from HFHS-fed *hGLP1R*^+/+^ versus *hGLP1R*^A316T/A316T^ mice. While liver samples from HFHS-fed *hGLP1R*^+/+^ mice showed accumulation of large lipid droplets indicative of macrovesicular steatosis, those from *hGLP1R*^A316T/A316T^ mice contained a higher number of small lipid droplets, suggestive of microvesicular steatosis (Fig. 4D). Quantifications confirmed a significant reduction in size but a near-significant increase in the number of lipid droplets in HFHS-fed *hGLP1R*^A316T/A316T^ mouse livers (Fig. 4E).

Overall, our results indicate a reduction in glucagon secretion concomitant to improved glucose tolerance of the A316T variant, leading to glucagon resistance in the liver associated with α cell hyperplasia and changes in liver steatosis under HFHS diet–triggered metabolic stress.

### Changes in cell surface expression, agonist-induced cAMP, and insulin secretion in human *GLP1R*^A316T/A316T^ mouse islets

Accounting for potential variations in cell surface expression of GLP-1R variants is crucial, as differences in this parameter can influence baseline receptor activity and subsequent GLP-1RA responses (*30*). Of note, the humanized *GLP1R* mouse model used in this study displays reduced levels of GLP-1R surface expression compared to nonhumanized mice (fig. S6, D and E), potentially due to *hGLP1R* being expressed as a mini-gene in the murine *Glp1r* locus (*32*). We therefore have restricted our comparisons to humanized *GLP1R* mice with the same genetic background to avoid any confounding effects.

**Fig. 5.**
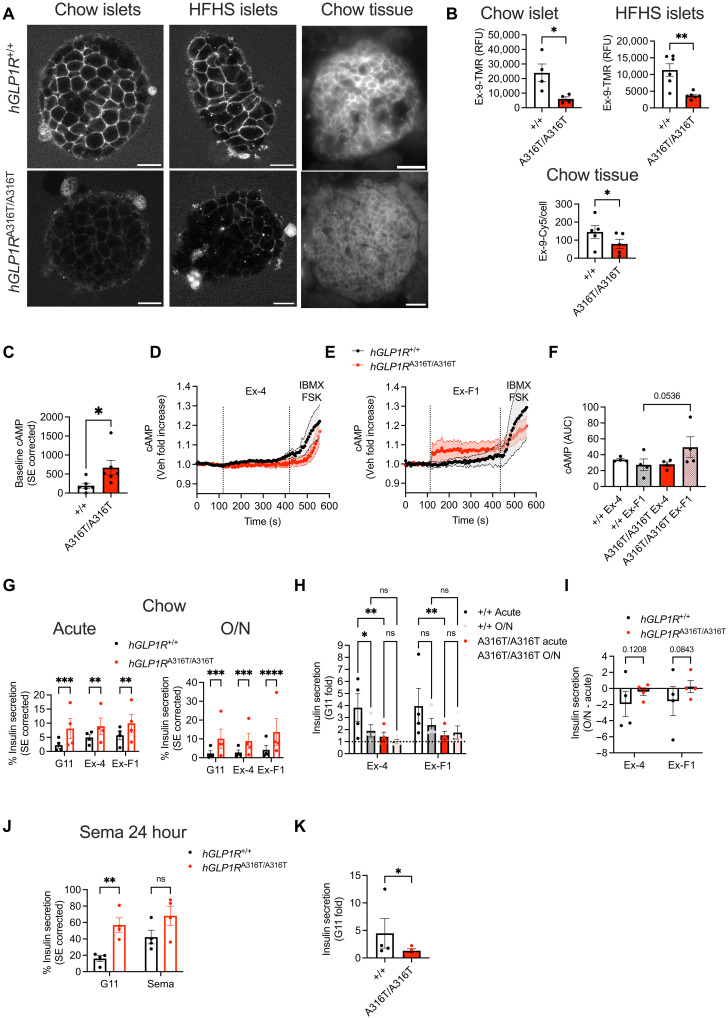
GLP-1R surface expression and downstream signaling in *hGLP1R*^A316T/A316T^ versus *hGLP1R*^+/+^ mouse islets. (**A** and **B**) GLP-1R surface expression, assessed ex vivo with exendin-9–TMR (Ex-9-TMR) in purified chow and HFHS islets or in vivo following subcutaneous injection of exendin-9–Cy5 (Ex-9-Cy5, LUXendin645) and fluorescence imaging of pancreatic tissue sections of *hGLP1R*^+/+^ versus ^A316T/A316T^ mice. Representative images (A) and quantifications of surface GLP-1R levels (B); scale bars, 20 μm; *n* = 4 to 6. RFU, relative fluorescence units. (**C**) Basal cAMP responses measured with the Green Up cADDis cAMP biosensor in chow-fed *hGLP1R*^+/+^ versus ^A316T/A316T^ mouse islets; *n* = 6. (**D** and **E**) cAMP responses to 100 nM exendin-4 (Ex-4) (D) or exendin-F1 (Ex-F1) (E) in islets from (C); responses normalized to baseline; IBMX and forskolin (FSK) added at the acquisition end for maximal responses; *n* = 4. (**F**) AUCs calculated from agonist-stimulated periods in (D) and (E); *n* = 4 per genotype. (**G**) Percentage of acute and overnight (O/N) surface expression (SE)–corrected insulin secretion in chow-fed *hGLP1R*^+/+^ versus ^A316T/A316T^ mouse islets stimulated with 11 mM glucose (G11) alone or supplemented with 100 nM exendin-4 (Ex-4) or exendin-F1 (Ex-F1); *n* = 4. (**H**) Insulin secretion fold changes to G11 calculated from data in (G). (**I**) Overnight over acute insulin secretion responses calculated from data in (H). (**J**) Percentage of SE-corrected insulin secretion in chow-fed *hGLP1R*^+/+^ versus ^A316T/A316T^ mouse islets stimulated for 24 hours with 11 mM glucose (G11) alone or supplemented with 100 nM semaglutide; *n* = 4. (**K**) Insulin secretion fold changes to G11 calculated from data in (J). Data are mean ± SEM; *P < 0.05, **P < 0.01, ***P < 0.001, and ****P < 0.0001; by paired *t* tests, one-, or two-way ANOVA with Sidak’s post hoc tests.

Islets extracted from both chow- and HFHS-fed *hGLP1R*^A316T/A316T^ mice showed reduced surface GLP-1R levels versus *hGLP1R*^+/+^, assessed by labeling with a saturating concentration of the fluorescent GLP-1R antagonist exendin-9–TMR. Results were corroborated in vivo by subcutaneous injection of exendin-9–Cy5 [LUXendin645 (*2*)] before transcardial fixation (Fig. 5, A and B).

We next analyzed acute cAMP responses in chow-fed *hGLP1R*^A316T/A316^ versus *hGLP1R*^+/+^ islets transduced with the baculoviral cAMP biosensor cADDis (*50*). Once corrected for surface expression, basal cAMP was significantly increased in *hGLP1R*^A316T/A316^ relative to *hGLP1R*^+/+^ islets (Fig. 5C). While agonist-induced responses were not significantly different between genotypes, we observed a trend for reduced cAMP in response to exendin-4, whereas, conversely, there was a near-significant increase in cAMP in response to exendin-F1 in *hGLP1R*^A316T/A316^ islets (Fig. 5, D to F). Ex vivo analyses of insulin secretion from the same islets showed increased outputs in response to both acute (30 min) and sustained (16 hours, cumulative) exposure to 11 mM glucose in *hGLP1R*^A316T/A316^ relative to *hGLP1R*^+/+^ (Fig. 5G). Agonist-mediated increases in glucose-stimulated insulin secretion, measured as fold over 11 mM glucose, were however blunted in *hGLP1R*^A316T/A316^ versus *hGLP1R*^+/+^ islets, with complete loss of the potentiating effect of exendin-4 but no significantly detrimental effect for exendin-F1 following overnight exposure (Fig. 5H). As in vivo, no differences were detected in prolonged minus acute responses for either agonist versus *hGLP1R*^+/+^ islets, although there was a tendency for the bulk of the A316T detrimental effect to occur acutely (Fig. 5I). Parallel experiments in islets from HFHS-fed mice showed similar tendencies which, however, did not reach statistical significance (fig. S6, F to H). Secretion responses to 24-hour stimulation with semaglutide were again significantly decreased in islets from HFHS-fed *hGLP1R*^A316T/A316^ versus *hGLP1R*^+/+^ mice, due to increased basal secretion from *hGLP1R*^A316T/A316^ islets exposed to 11 mM glucose alone (Fig. 5, J and K). The increased basal insulin secretion profile of A316T was likely due to constitutively raised activity of the variant receptor as it was significantly reduced ex vivo in islets exposed to exendin-9, a compound known to act both as an antagonist (*51*) and as an inverse agonist for the GLP-1R (fig. S6I) (*52*).

Further experiments were performed using islets from *hGLP1R*^−/−^ mice, generated in house from the *hGLP1R*^+/+^ mouse line, subsequently transduced with adenoviral vectors expressing SNAP/FLAG-tagged WT or A316T hGLP-1R (pAV-SNAP/FLAG-*hGLP1R*^WT^ or ^A316T^). Using a membrane-impermeable fluorescent SNAP tag probe, we again measured reduced surface expression of SNAP/FLAG-*hGLP1R*^A316T^ compared to WT (fig. S7, A and B). Intracellular Ca^2+^ mobilization, another downstream signaling readout associated with insulin granule exocytosis, was examined using the fluorescent calcium indicator Cal-520, AM. A clear increase in basal Ca^2+^ levels was apparent in SNAP/FLAG-*hGLP1R*^A316T^ versus SNAP/FLAG-*hGLP1R*^WT^-expressing islets (fig. S7C). Agonist-induced Ca^2+^ responses were not significantly different between WT and A316T for GLP-1, exendin-4, or semaglutide but were increased in response to exendin-F1 in *hGLP1R*^−/−^ pAV-SNAP/FLAG-*hGLP1R*^A316T^ islets (fig. S7, D to H), in line with our previous cAMP results with this biased GLP-1RA. Overall, the A316T variant was associated with enhanced islet basal cAMP and insulin secretion responses but loss of action of unbiased GLP-1RAs, with some preservation associated with the Gα_s_-biased agonist exendin-F1.

### Impact of the A316T variant on primary human islets and a human β cell line

**Fig. 6.**
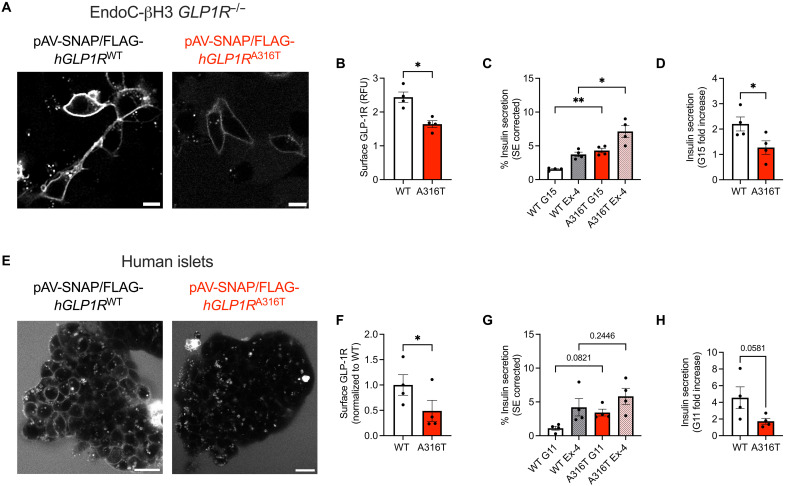
SNAP/FLAG-*hGLP1R*^WT^ versus ^A316T^ surface expression levels and insulin secretion in human β cell models. (**A** and **B**) Surface expression in EndoC-βH3 *GLP1R*^−/−^-enriched cells transduced with pAV-SNAP/FLAG-*hGLP1R*^WT^ or ^A316T^ labeled with SNAP-Surface Alexa Fluor 647. Representative images (A) and quantification of surface GLP-1R (B); scale bars, 10 μm; *n* = 4. (**C**) Percentage of surface expression (SE)–corrected insulin secretion in EndoC-βH3 *GLP1R*^−/−^-enriched cells transduced with pAV-SNAP/FLAG-*hGLP1R*^WT^ or ^A316T^ in response to 15 mM glucose (G15) alone or supplemented with 100 nM exendin-4 (Ex-4); *n* = 4. (**D**) Insulin secretion fold changes to G15 calculated from data in (C). (**E** and **F**) Surface expression in primary human islets transduced with pAV-SNAP/FLAG-*hGLP1R*^WT^ or ^A316T^ labeled with SNAP-Surface Alexa Fluor 647. Representative images (E) and quantification of surface GLP-1R (F); scale bars, 20 μm; *n* = 4. (**G**) Percentage of SE-corrected insulin secretion in primary human islets transduced with pAV-SNAP/FLAG-*hGLP1R*^WT^ or ^A316T^ in response to 11 mM glucose (G11) alone or supplemented with 100 nM exendin-4 (Ex-4); *n* = 4. (**H**) Insulin secretion fold changes to G11 calculated from data in (G). Data are mean ± SEM; *P < 0.05, **P < 0.01; by paired *t* tests or one-way ANOVA with Sidak’s post hoc tests.

Given the strong evidence from previous GWAS and in vitro human embryonic kidney (HEK) 293 data (*53*), and our in vivo observations of the effect of the A316T variant on glucose homeostasis, we next investigated its impact on human β cell models, including human donor islets and the well-characterized human β cell line EndoC-βH3 (*54*), genetically modified by CRISPR-Cas9 to generate a partial enrichment in *GLP1R*^−/−^ cells (see Materials and Methods and fig. S1D), both subsequently transduced with adenoviruses expressing SNAP/FLAG-*hGLP1R*^WT^ or ^A316T^. EndoC-βH3 *GLP1R*^*−*/*−*^-enriched SNAP/FLAG-*hGLP1R*^A316T^ cells again showed reduced GLP-1R cell surface expression (Fig. 6, A and B) and a similar insulin secretion profile to *hGLP1R*^A316T/A316T^ mouse islets, with enhanced basal insulin secretion but significantly reduced exendin-4–stimulated secretory fold increases (Fig. 6, C and D). Primary human islets transduced with SNAP/FLAG-*hGLP1R*^WT^ or ^A316T^ adenoviruses, a system that mimics a heterozygous phenotype for the A316T variant, again showed reduced surface expression of SNAP/FLAG-*hGLP1R*^A316T^ versus ^WT^ (Fig. 6, E and F), as well as a near-significant loss of exendin-4–induced potentiation of insulin secretion (Fig. 6, G and H). Both human A316T models retained some degree of exendin-4 responsiveness compared to glucose alone responses, for the most part absent in islets from *hGLP1R*^A316T/A316^ mice, an effect that we ascribe to the (at least partial) coexpression of WT and A316T GLP-1Rs in these models.

### Modulation of GLP-1R trafficking by the A316T variant

**Fig. 7.**
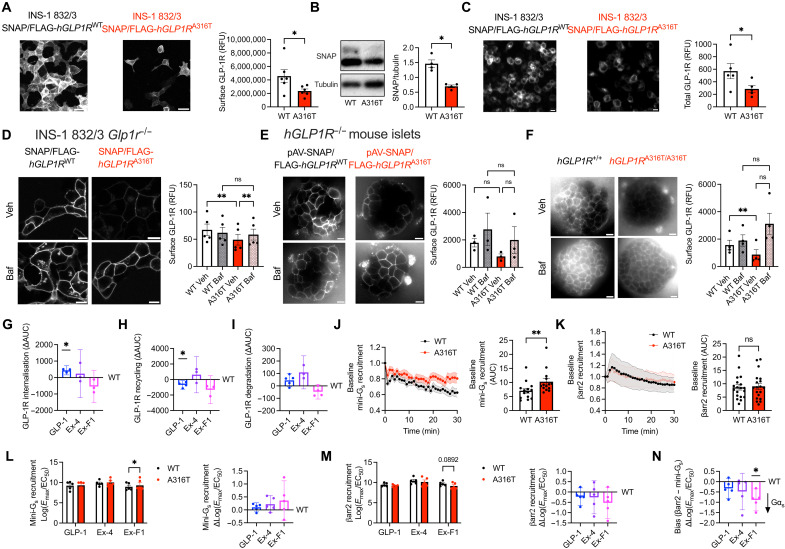
Functional characterization of INS-1 832/3 *Glp1r*^−/−^ SNAP/FLAG-*hGLP1R*^WT^ versus ^A316T^ sublines. (**A**) SNAP/FLAG-*hGLP1R*^WT^ versus ^A316T^ surface expression; scale bars, 10 μm; *n* = 6. (**B** and **C**) Total GLP-1R expression, by Western blotting (B) or BG-OG (C); *n* = 4 for (B), *n* = 5 for (C); scale bars, 10 μm. (**D**) Surface GLP-1R expression following 2 hours vehicle (Veh) or 400 nM bafilomycin A1 (Baf) exposure; scale bars, 10 μm; *n* = 5. (**E**) As in (D) for *hGlp1r*^−/−^ islets transduced with pAV-SNAP/FLAG-*hGLP1R*^WT^ or ^A316T^; scale bars, 10 μm; *n* = 3. (**F**) As in (D) for *hGLP1R*^+/+^ versus ^A316T/A316T^ islets labeled with LUXendin645; scale bars, 10 μm; *n* = 4. (**G**) GLP-1R internalization **Δ**AUC [SNAP/FLAG-*hGLP1R*^A316T^ minus ^WT^, calculated from fig. S8 (A and B)]; *n* = 3. (**H**) GLP-1R plasma membrane recycling **Δ**AUC, calculated from fig. S8 (C and D); *n* = 3-4. (**I**) GLP-1R degradation **Δ**AUC, calculated from fig. S8 (E and F); *n* = 3 to 5. (**J**) Baseline LgBiT-mini-G_s_ recruitment to *hGLP1R*^WT^ or ^A316T^-SmBiT in INS-1 832/3 *Glp1r*^−/−^ cells; responses over time and AUCs shown; *n* = 14. (**K**) Baseline LgBiT–β-arrestin 2 (βarr2) recruitment to *hGLP1R*^WT^ or ^A316T^-SmBiT; *n* = 19. (**L**) LgBiT–mini-G_s_ recruitment to *hGLP1R*^WT^ or ^A316T^-SmBiT with indicated agonists; log(*E*_max_/EC_50_) calculated from fig. S8 (G and H); SNAP/FLAG-*hGLP1R*^A316T^ minus ^WT^ **Δ**log(*E*_max_/EC_50_) shown; *n* = 5. (**M**) LgBiT–β-arrestin 2 (βarr2) recruitment to *hGLP1R*^WT^ or ^A316T^-SmBiT with indicated agonists; log(*E*_max_/EC_50_) calculated from fig. S8 (I and J); SNAP/FLAG-*hGLP1R*^A316T^ minus ^WT^ **Δ**log(*E*_max_/EC_50_) shown; *n* = 5. (**N**) LgBiT–β-arrestin 2 minus LgBiT–mini-G_s_ (βarr2 − mini-G_s_) recruitment to *hGLP1R*^A316T^ minus ^WT^-SmBiT, shown as **Δ**log(*E*_max_/EC_50_). Data are mean ± SEM except for **Δ**responses which are mean ± 95% confidence interval; *P < 0.05 and **P < 0.01; by paired *t* tests, one-way ANOVA with Sidak’s, or two-way ANOVA with Tukey’s post hoc tests.

Having characterized the functional effects of the A316T variant in vivo and ex vivo, we next explored the molecular mechanisms underlying the observed changes in A316T GLP-1R cell surface expression and signaling. To do so, we used an in vitro INS-1 832/3 rat β cell model where endogenous GLP-1R expression is fully deleted by CRISPR-Cas9 (*55*), to generate stable multiclonal cell lines expressing SNAP/FLAG-tagged WT or A316T hGLP-1R. Similarly to our previous findings, SNAP/FLAG-*hGLP1R*^A316T^ cells showed reduced receptor surface expression versus its WT counterpart (Fig. 7A). Assessment of SNAP/FLAG-*hGLP1R*^WT^ versus ^A316T^ expression by Western blotting, or with the cell-permeable SNAP-tag probe BG-OG (*2*), also revealed significantly reduced total levels of the A316T variant compared to WT receptor (Fig. 7, B and C), suggesting increased receptor degradation or reduced protein synthesis. To clarify the mechanism underlying this phenotype, we used bafilomycin A1, an established vacuolar-type H^+^-dependent adenosine triphosphatase (H^+^-ATPase) inhibitor that prevents lysosomal degradation (*56*). Bafilomycin A1 exposure resulted in complete recovery of surface expression levels in INS-1 832/3 *Glp1r*^−/−^ SNAP/FLAG-*hGLP1R*^A316T^ cells, now comparable to WT (Fig. 7D). The same effect was observed using *hGLP1R*^−/−^ mouse islets transduced with SNAP/FLAG-*hGLP1R*^WT^ versus ^A316T^ adenoviruses (Fig. 7E) or islets purified from *hGLP1R*^+/+^ versus ^A316T/A316T^ mice (Fig. 7F), indicating increased basal turnover resulting in enhanced lysosomal degradation as the cause for the decrease in surface and total GLP-1R A316T. Next, we assessed GLP-1R trafficking behaviors in response to GLP-1RA stimulation in INS-1 832/3 *Glp1r*^−/−^ SNAP/FLAG-*hGLP1R*^WT^ versus ^A316T^ cells by high-content microscopy. A316T GLP-1R internalization was similar to WT in response to exendin-4 and exendin-F1 but was significantly increased with the endogenous agonist GLP-1, reflecting potentially faster kinetics at earlier time points (Fig. 7G and fig. S8, A and B). A316T receptor recycling to the plasma membrane was again not different from WT with exendin-4 or exendin-F1 but was significantly reduced for GLP-1 (Fig. 7H and fig. S8, C and D). Last, receptor degradation in response to agonist stimulation was not significantly different between WT and A316T for any of the agonists tested (Fig. 7I and fig. S8, E and F).

Overall, the A316T variant receptor exhibited increased basal turnover leading to enhanced lysosomal degradation under vehicle conditions, as well as a specific trafficking pattern in response to GLP-1 which might potentially account for the improved oral glucose tolerance (which depends on endogenous GLP-1 action) observed in these mice.

### A316T-associated changes on GLP-1R coupling to Gα_s_, β-arrestin 2, and downstream signaling effectors

We next explored changes in the GLP-1R A316T capacity for Gα_s_ and β-arrestin 2 coupling using NanoBiT complementation assays in INS-1 832/3 *Glp1r*^−/−^ SNAP/FLAG-*hGLP1R*^WT^ versus ^A316T^ cells. We first analyzed mini-G_s_ and β-arrestin 2 recruitment to WT versus A316T receptors under vehicle conditions to evaluate changes in baseline coupling activity. The A316T GLP-1R demonstrated significantly increased basal mini-G_s_, with no change in basal β-arrestin 2 recruitment compared to WT (Fig. 7, J and K). No significant changes versus WT were found for A316T mini-G_s_ recruitment in response to GLP-1 or exendin-4, but we measured increased potency for A316T mini-G_s_ recruitment in response to exendin-F1 (Fig. 7L and fig. S8, G and H). Agonist-induced recruitment of β-arrestin 2 was not significantly different for A316T versus WT besides a near-significant reduction with exendin-F1 (Fig. 7M and fig. S8, I and J). When both effects were combined to calculate β-arrestin 2 over Gα_s_ signaling bias, we found a significant shift toward Gα_s_ recruitment for A316T versus WT only with exendin-F1 (Fig. 7N). As changes in GLP-1R cAMP are often the result of combined trafficking and Gα_s_/β-arrestin 2 coupling effects, we next analyzed cAMP responses to the different GLP-1RAs in INS-1 832/3 *Glp1r*^−/−^ SNAP/FLAG-*hGLP1R*^WT^ versus ^A316T^ cells. While we could not detect significant differences, there was a tendency for increased cAMP in response to GLP-1 and exendin-F1 but reduced responses to exendin-4 and no change for semaglutide in A316T versus WT receptors in this in vitro model (fig. S8, K to P).

**Fig. 8.**
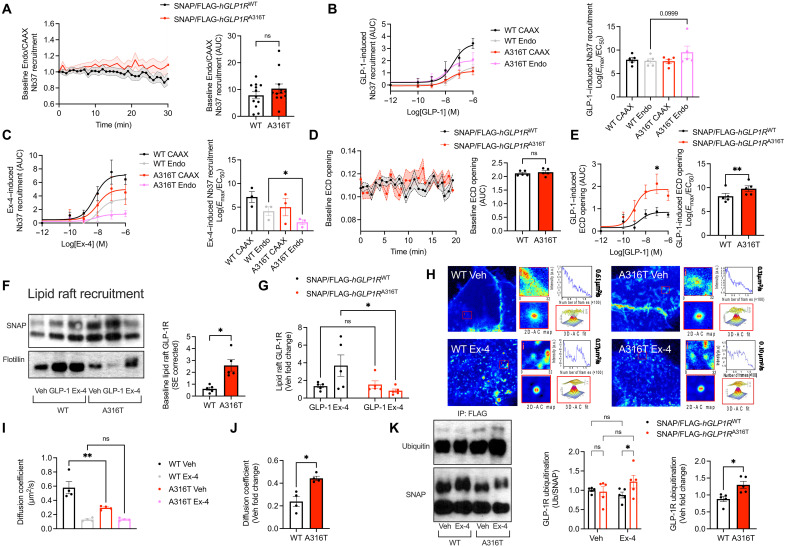
Molecular characterization of INS-1 832/3 *Glp1r*^−/−^ SNAP/FLAG-*hGLP1R*^WT^ versus ^A316T^ sublines. (**A**) Baseline endosomal over plasma membrane GLP-1R activity, measured by SmBiT-Nb37 complementation with Endofin-LgBiT over LgBiT-CAAX under vehicle conditions; AUCs also shown; *n* = 12. (**B**) AUC dose-response curves and log(*E*_max_/EC_50_) of plasma membrane and endosomal GLP-1R activity in response to GLP-1 stimulation, measured by SmBiT-Nb37 complementation with LgBiT-CAAX and Endofin-LgBiT; *n* = 4. (**C**) AUC dose-response curves and log(*E*_max_/EC_50_) of plasma membrane and endosomal GLP-1R activity in response to exendin-4 (Ex-4) stimulation, measured by SmBiT-Nb37 complementation with LgBiT-CAAX and Endofin-LgBiT; *n* = 3. (**D**) Baseline GLP-1R ECD opening, measured by SNAP-Lumi4-Tb–NR12A TR-FRET over time under vehicle conditions; AUCs also shown; *n* = 5. (**E**) AUC dose-response curves and log(*E*_max_/EC_50_) of GLP-1R ECD opening in response to GLP-1 stimulation, measured by SNAP-Lumi4-Tb–NR12A TR-FRET increases over time; *n* = 5. (**F**) Representative blots showing SNAP/FLAG-*hGLP1R* recruitment to detergent-resistant membrane (DRM) fractions (or lipid rafts) following stimulation for 2 min with indicated agonist, with Flotillin used as DRM loading control; quantification of surface expression (SE)–corrected baseline SNAP/FLAG-*hGLP1R*^WT^ versus ^A316T^ DRM levels under vehicle conditions shown; *n* = 5. (**G**) SNAP/FLAG-*hGLP1R*^WT^ versus ^A316T^ DRM recruitment fold changes to vehicle (Veh), quantified from (F); *n* = 5. (**H**) Representative images from RICS analysis of GLP-1R WT and A316T plasma membrane lateral diffusion in cells labeled with SNAP-Surface Alexa Fluor 647 and subsequently stimulated with vehicle (Veh) or 100 nM Ex-4. (**I** and **J**) GLP-1R WT versus A316T average RICS diffusion coefficients (I) and vehicle (Veh) fold changes (J); *n* = 4. (**K**) Representative blots showing SNAP/FLAG-*hGLP1R*^WT^ versus ^A316T^ ubiquitination following anti-FLAG immunoprecipitation in cells under vehicle (Veh) or 100 nM Ex-4 stimulation for 10 min; quantification of GLP-1R ubiquitination and vehicle (Veh) fold changes shown; *n* = 5. a.u., arbitrary units.

We next used a previously described bystander NanoBiT assay, based on the recruitment of active Gα_s_-binding nanobody 37 (Nb37) to plasma membrane versus endosomal locations (*57*) to assess the effect of the A316T variant on subcellular receptor activity. Results showed a nonsignificant tendency for increased basal endosomal over plasma membrane activity of A316T compared to WT (Fig. 8A). In addition, while agonist-induced endosomal A316T activity showed a tendency to be increased versus WT with GLP-1, this was significantly reduced in response to exendin-4, without changes in plasma membrane activity versus WT with either agonist (Fig. 8, B and C).

We also tested potential changes in receptor conformational dynamics, indicative of receptor-agonist binding and activation, using a time-resolved fluorescence resonance energy transfer (TR-FRET) assay that measures the proximity between the receptor extracellular domain (ECD) and the plasma membrane as a surrogate for receptor ECD opening. While no changes were detected under basal conditions (Fig. 8D), significantly increased TR-FRET was measured for A316T versus WT in response to GLP-1 stimulation (Fig. 8E), again highlighting increased engagement of the A316T receptor with the endogenous agonist.

We next determined the impact of the A316T mutation on GLP-1R recruitment to plasma membrane lipid nanodomains, or “rafts,” a preferential localization for active GLP-1Rs before being internalized (*58*). In line with our previous findings of a higher level of constitutive receptor activity, the A316T variant presented with increased basal levels at lipid rafts (Fig. 8F). While there were no changes in raft segregation in response to GLP-1 for A316T versus WT, raft localization was significantly reduced for A316T in response to exendin-4 (Fig. 8G). Given the close association between receptor activation, lipid raft segregation, plasma membrane diffusion, and clustering (*58*, *59*), we next investigated changes in these parameters by Raster image correlation spectroscopy (RICS) analysis of INS-1 832/3 *Glp1r*^−/−^ SNAP/FLAG-*hGLP1R*^WT^ versus ^A316T^ cells. Results showed significantly reduced plasma membrane diffusion for A316T versus WT GLP-1R under vehicle conditions, an effect normally observed only following agonist stimulation in WT receptors (*60*), as also seen here (Fig. 8, H to J). Last, we analyzed changes in GLP-1R ubiquitination, a key posttranslational modification linked to changes in GLP-1R signaling and postendocytic trafficking (*61*). As previously seen (*61*), GLP-1R was basally ubiquitinated under vehicle conditions for both WT and A316T receptors. While exendin-4 stimulation did not affect the WT ubiquitination level, it significantly increased ubiquitination of the A316T receptor (Fig. 8K), a feature typically associated with lysosomal targeting for degradation and signal termination (*62*).

Overall, these results unveiled a constitutively increased signaling profile for the A316T receptor, with parameters such as increased Gα_s_ coupling and lipid raft recruitment but reduced plasma membrane diffusion under vehicle conditions. Results also indicated enhanced exendin-4–induced A316T desensitization, with reduced lipid raft recruitment and endosomal signaling but increased receptor ubiquitination linked to degradation with this GLP-1RA.

Next, to assess the global signaling effect of the GLP-1R A316T variant at a cellular level, we performed a mass spectrometry–based interactome analysis of vehicle versus agonist (GLP-1 or exendin-4)–stimulated A316T GLP-1R using INS-1 832/3 *Glp1r*^−/−^ SNAP/FLAG-*hGLP1R*^A316T^ in parallel with SNAP/FLAG-*hGLP1R*^WT^ cells. Results normalized to isolated receptor amounts per immunoprecipitate revealed increased GLP-1R–interactor binding across the whole interactome for A316T compared to WT under vehicle conditions, indicative of the level of basal activation inherent to each receptor type (fig. S9). Stimulation of A316T with either GLP-1 or exendin-4 led to further increases in GLP-1R binding for most interactors but with some factors exhibiting reduced binding to active versus inactive A316T receptors, reversing the normal pattern of increased binding following GLP-1RA stimulation of WT receptors. These included, notably, the endoplasmic reticulum membrane contact site tether VAPB and the A-kinase anchoring protein SPHKAP, two GLP-1R binding partners for which we have recently identified a critical role in the control of protein kinase A signaling and mitochondrial function (*63*).

### Structural determination of the GLP-1R A316T variant

**Fig. 9.**
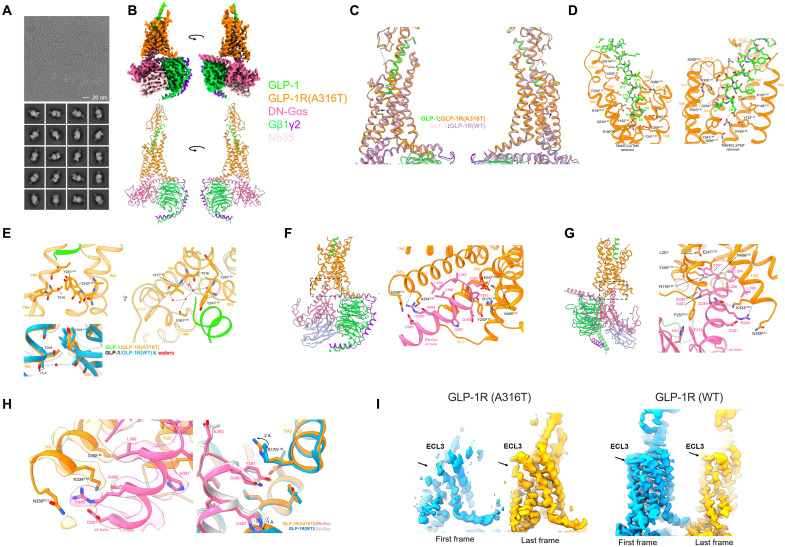
GLP-1:GLP-1R A316T:DN-Gα_s_:Nb35 cryo-EM structure. (**A**) Cryo-EM complex (top); 2D class averages from consensus refinements (bottom). (**B**) Nonuniform refined density map (top), with atomic model including ECD backbone (bottom). (**C**) GLP-1R A316T (orange) and WT (lavender) TMDs overlaid, left; 140° rotation, right; black arrows indicate residue 316. (**D**) Binding arrangement of GLP-1 residues 7 to 31 (green) with GLP-1R A316T (orange), facing inward from TM5 (left), and from TM6 (right); dashed lines, H-bonds. (**E**) Top left: Positioning of T316^5.46^ and Y241^3.44^ below GLP-1 (green); bottom left: comparison between T/A316^5.46^ and Y241^3.44^ local arrangement in A316T (orange) and WT GLP-1R (blue; PDB 6X18) (bottom left); a structural water (red) from 2.1-Å WT structure has H-bond interactions (dashed lines) with these residues; black arrow indicates movement of T316 carbonyl away from A316^5.46^; 2-Å zoned density map (3.3-Å resolution) of GLP-1R A316T structure, transparent gray surface; right: intracellular-facing receptor core, showing TM3-TM5-TM6 structural water network (red) from PDB 6X18. Red cross, potential H-bond lost for T316. TM1, TM2, and TM7 removed for clarity. (**F**) Key interactions between GLP-1R A316T intracellular cavity (orange) and α5 helix of DN-Gα_s_ (pink), with “forward” view indicated by left cartoon; H-bonds, dashed lines. (**G**) Alternative view of GLP-1R A316T–DN-Gα_s_ α5 helix interactions, as indicated by left cartoon. (**H**) Left: Cryo-EM density map used to model interactions (colored transparent silhouettes). There is no density for N338 ICL3, with nearby side chains well-resolved; right: comparison between GLP-1R A316T–α5 helix (orange and pink) and GLP-1R WT–α5 helix (blue and white) interactions. (**I**) Left: Close-up of first and last frames from principal component 3 after 3DVA of GLP-1R A316T, with distant density clipped for clarity; right: principal component 3 from 3DVA for GLP-1R WT; raw data from (*64*); ECL3, bold arrows.

To elucidate the structural changes associated with the A316T variant receptor, a cryo-EM structure of the GLP-1–bound GLP-1R A316T in complex with dominant negative (DN)–Gα_s_ was determined at 3.3-Å resolution (Fig. 9, A and B). In general, the complex was well resolved, and amino acid side chains were modeled for almost the entire structure. The α-helical domain of DN-Gα_s_, the flexible ECD, the extracellular loop 3 (ECL3), and the intracellular loop 3 (ICL3) of GLP-1R A316T, as well as the final two C-terminal residues of GLP-1, were less well resolved in the cryo-EM map, in line with the intrinsic flexibility of these regions. The overall positioning of the A316T transmembrane domains (TMDs) was highly similar to that of the WT receptor [Protein Data Bank (PDB) 6X18; Fig. 9C] and the binding arrangement of GLP-1 at the backbone level essentially identical to that previously reported for WT GLP-1R by high-resolution cryo-EM (Fig. 9D) (*64*). In the WT GLP-1R structure, the C terminus of GLP-1 interacts with the ECD and ECL1/2 of GLP-1R, forming extensive contacts with transmembrane (TM) helices 1, 2, 3, and 5, enabling the peptide N-terminal residues to engage deep into the orthosteric site with a conserved class B1 GPCR central polar network, including a salt bridge with R190^2.60^. A network of structural waters connects the central parts of TM3, TM5, and TM6 below the peptide binding site, forming hydrogen bonds (H-bonds) with the receptor backbone, including with the highly conserved G361^6.50^ at the P^6.47^xxG^6.50^ motif (*64*), crucial for receptor activation and transducer coupling. In addition, the backbone carbonyl of Y241^3.44^, part of the conserved central polar network, directly interfaces through a H-bond with a structural water located between TM3 and TM5, within the H-bond distance of the backbone carbonyl of A316^5.46^, contributing to increase the interhelical packing and stiffness. In the T316 variant, however, this backbone carbonyl sits ~1.5 Å away from the structural water and has a shallower angle compared to A316^5.46^, making it poorly favorable for H-bonding (Fig. 9E). When modeled as the “upward” rotamer, the hydroxyl group of T316^5.46^ can H-bond with the oxygen of this structural water, likely precluding the known interactions discussed above. The interface between the GLP-1R and Gα_s_, highly conserved with other class B1 GPCRs (*65*), is preserved in the A316T GLP-1R, with interactions between the variant receptor and the α5 helix of Gα_s_ very similar to those found in WT GLP-1RA–bound structures (Fig. 9, F and G) (*64*). Higher-resolution structures of agonist-bound GLP-1R in complex with Gα_s_ have uncovered important structural waters in this region, including one acting as a key bridge between E247^3.50^, in the highly conserved HETX motif, and Y391^Gαs^. At this resolution, these waters were not present in the density map of GLP-1R A316T, with E247^3.50^ poorly defined compared to neighboring residues of similar size, which is not unusual for an acidic residue in single-particle cryo-EM at 3.3-Å resolution. N338^ICL3^ was also poorly resolved, indicating that this residue is comparatively mobile (Fig. 9H, left graph). The position of R176^2.46^ is rotated toward the α5 helix of Gα_s_ by 2 Å in GLP-1R A316T compared to WT, and, independently, H387 is rotated slightly from its position in the WT GLP-1R structure (Fig. 9H, right graph). Overall, we observed subtle differences that may contribute to the altered Gα_s_ recruitment sometimes observed with this mutant (*25*, *28*). In summary, the static model built into the consensus density map of the GLP-1:GLP-1R(A316T):DN-Gα_s_:Nb35 complex, while largely similar to WT, suggests changes to key water-mediated interactions between TM5 and its neighboring helices that could alter the dynamics of the TMD. In addition, subtle changes to interactions between the intracellular face of the receptor and the α5 helix of DN-Gα_s_ may correlate with the pharmacological profile of the A316T variant.

To explore these observations further, three-dimensional variability analysis (3DVA) was performed in cryoSPARC 3.2, a powerful tool for examining conformational heterogeneity in cryo-EM datasets (*66*). These data were analyzed by a principal components analysis model searching for three principal components with a tight mask around the receptor and G proteins to exclude variability from the detergent micelle. Previous analyses of GLP-1R cryo-EM structures with peptide ligands have demonstrated three major modes of motion: a coordinated twisting movement of the receptor TMD, a large oscillation of the peptide C terminus and ECD, and a twisting/rocking of the receptor and G protein (*64*). A notable increase in variability for ECL3 in GLP-1R A316T compared to the WT receptor was observed in all three principal components and was preserved at low contour. This motion was particularly evident in principal component 3 and was accompanied by more extreme movements in TM6 and the N-terminal half of the peptide (Fig. 9I). Compared to the 3DVA of GLP-1R WT, GLP-1R (A316T) TM6-ECL3-TM7 underwent a larger motion down and away from the receptor core (Fig. 9I).

### MD simulations of WT versus A316T GLP-1R: Impact of the A316T SNP on receptor dynamics, structural water molecules, and H-bond interactions

**Fig. 10.**
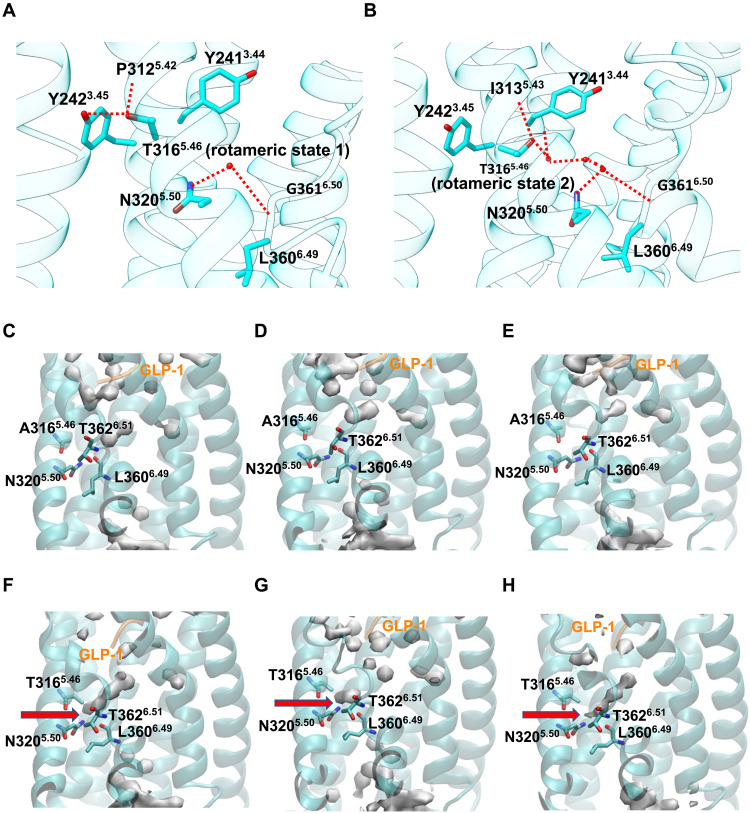
Divergent H-bond and solvation patterns in WT versus A316T GLP-1R. (**A**) T316^5.46^ rotameric state 1 forms a stable H-bond with Y242^3.45^. (**B**) T316^5.46^ rotameric state 2 is involved in water-mediated interactions with the polar core of the receptor. N320^5.50^ forms stable water bridges with TM6 kink (L360^6.49^ and G261^6.50^) in A316T. (**C** to **E**) Volumetric map (isovalue, 0.3) of water molecules within the TMD of WT GLP-1R (cyan ribbon) in complex only with the endogenous agonist GLP-1 (orange ribbon) from three independent MD simulation replicas. (**F** to **H**) Volumetric map (isovalue, 0.3) of water molecules within the TMD of A316T (cyan ribbon) in complex only with the endogenous agonist GLP-1 (orange ribbon) from three independent MD simulation replicas. The red arrows indicate the position of stable water molecules interacting with N320^5.50^ and the TM6 kink.

To retrieve atomistic insights about the effect of the A316T substitution on the structure and dynamics of the GLP-1R, A316T and WT GLP-1Rs were simulated in three different configurations: (i) the isolated TMD in the absence of any agonist; (ii) in complex with the endogenous agonist GLP-1 [i.e., the new A316T cryo-EM structure presented here compared to PDB 6X18 (fig. S10A)]; and (iii) in complex with exendin-F1 and Gα_s_ (i.e., the WT cryo-EM structure presented in PDB 9C0K and a modeled A316T structure based on the former). In the TMD, the side chain of T316^5.46^ (A316T) formed a H-bond with Y242^3.45^, an interaction which was not present in WT GLP-1R, where Y242^3.45^ interacted with the P312^5.42^ backbone instead (Fig. 10A and fig. S10B). This A316T-specific Y242^3.45^-T316^5.46^ H-bond (table S1) was very persistent in the isolated TMD (85%), while it was less persistent but still present for more than one-third of simulations in A316T GLP-1R in complex with either GLP-1 or with exendin-F1 and Gα_s_ (41.8 and 37.7%, respectively). This suggests that the engagement of Y242^3.45^ with the hydrophilic side chain of T316^5.46^ instead of the P312^5.42^ backbone occurs in a ligand-dependent manner. The reason for the Y242^3.45^-T316^5.46^ H-bond strength in the isolated TMD of A316T lies in the rotameric state of T316^5.46^ adopting a configuration prone to accept the H-bond from Y242^3.45^ and donating the hydrogen to the P312^5.42^ backbone (state 1 in Fig. 10A, table S1, and movie S1). In A316T GLP-1R in complex with exendin-F1 and Gα_s_, the persistence of the Y242^3.45^-T316^5.46^ H-bond was similar to that in A316T GLP-1R in complex with GLP-1, but the Y242^3.45^-P312^5.42^ and T316^5.46^-P312^5.42^ interactions were similar to those found in the isolated A316T GLP-1R TMD, suggesting different local GLP-1R A316T dynamics with exendin-F1 or with the presence of bound Gα_s_. For the A316T GLP-1R in complex with GLP-1, the T316^5.46^ side chain flipped between rotameric state 1 and another conformation able to form a H-bond with the backbone of I313^5.43^ and a water bridge with the Y241^3.44^ backbone (state 2 in Fig. 10B and tables S1 and S2). This H-bond reshuffling is consistent with our previous MD simulations of oxyntomodulin and Gα_s_-complexed WT versus A316T GLP-1R (*25*), where the Y242^3.45^-P312^5.42^ H-bond in WT (88.9% occupancy) was replaced by the Y242^3.45^-T316^5.46^ H-bond in A316T (62.3% occupancy) due to a consistent rotameric state 1 throughout the simulations, corroborating an allosteric effect of the agonist in the T316^5.46^ dynamics and, in turn, Y242^3.45^-T316^5.46^ H-bond. Two clear rotameric states for T316^5.46^ were also observed in our consensus cryo-EM maps, able to accommodate both an upward and a “downward” side-chain orientation (Fig. 9), supporting the existence of both rotameric states of A316T occupied with similar frequencies in the presence of GLP-1.

T316^5.46^ (A316T) in rotameric state 2 weakly interacted with the conserved N320^5.50^ through a water bridge (Fig. 10B, table S2, and movie S1). This was also the case for GLP-1R (WT) bound to GLP-1, but here, rotameric state 2 favored a higher persistence of H-bond interactions with a water network lining T316^5.46^ and N320^5,50^. A detailed analysis of the interaction involving N320^5.50^ (table S3 and movie S1) reveals a stable direct H-bond with the backbone of L360^6.49^ at the TM6 kink level in the 3 WT GLP-1R simulations [occupancy 68.5% (isolated TMD), 79.6% (with GLP-1), and 65.4% (with exendin-F1 and Gα_s_)]. This N320^5.50^-L360^6.49^ H-bond is weaker in A316T when in complex with the endogenous agonist GLP-1 (35.6%) and for the isolated TMD (30.6%) but only slightly weaker in the case of A316T in complex with exendin-F1 and Gα_s_ (54.8%) (movie S1), in line with different local dynamics imprinted by exendin-F1. The diminished H-bond strength is counterbalanced by an increase in water-mediated interactions between N320^5.50^ and L360^6.49^ and between N320^5.50^ and G361^6.50^, in A316T in complex with GLP-1 (12.2% in WT versus 26.8% in A316T) and the isolated TMD (19.5% in WT versus 49.3% in A316T). Such water-mediated interactions involving N320^5.50^ in A316T were due to a deeper water molecule permeation in the polar core of the receptor compared to WT GLP-1R (Fig. 10, C to H), stabilized by a concerted effect of the polar side chain of T316^5.46^ and the agonist presence. A significant increase of up to 15% in the water molecule residence time was observed in A316T in complex with GLP-1 or with exendin-F1 and Gα_s_ (table S4) but not in the isolated TMD, where the water was exchanged with bulk solvent instead of being stabilized into structural water molecules, as occurring in the agonist-complexed A316T configurations.

To retrieve further insights into the allosteric effect of the persistent Y242^3.45^-T316^5.46^ H-bond formed by T316^5.46^ in rotameric state 1, we further analyzed the MD simulations involving the isolated TMD of GLP-1R. We reasoned that the GLP-1R structure other than the TMD (i.e., ECD and ECL1), or the binders GLP-1 and Gα_s_, can either directly (GLP-1, Gα_s_) or allosterically (ECD, ECL1) influence the possible conformations that the TMD adopts because of the A316T mutation, de facto shielding any subtle allosteric effects. The Y242^3.45^-T316^5.46^ H-bond in A316T moved TM3 and TM5 away from each other, as shown by the increased backbone distance between the closely downstream residues L245^3.48^-V319^5.49^ (fig. S10, B and C). This is consistent with our cryo-EM maps of A316T in complex with GLP-1 and Gα_s_, where the bulkier T316^5.46^ side chain sterically displaces the T316^5.46^ backbone carbonyl ~1.5 Å away from the interhelical space compared to WT A316^5.46^ (Fig. 9E). Structural contractions or expansions at the extracellular or intracellular side of a GPCR can allosterically influence the receptor dynamics at the opposite side (*67*). Consistent with this GPCR structural effect, in A316T GLP-1R, the TM3-TM5 intracellular distance, measured at the L251^3.54^-V327^5.57^ level, was ~1 Å shorter compared to that of WT GLP-1R (fig. S10, B and C), favored by a more persistent H-bond between Y252^3.55^ and R326^5.56^ (35.7% occupancy in A316T versus 11.4% in WT; fig. S10B). In concert with the TM3-TM5 intracellular contraction, the TM6 outward opening, a structural hallmark of GPCR activation to accommodate the Gα_s_ protein, was about 2.5 Å wider in A316T than in WT (27.65 ± 2.02 Å versus 25.12 ± 1.80 Å, measured between the Cα atoms of C347^6.36^ and E408^7.63^, fig. S10D). As a reference, in the static cryo-EM structure of A316T, and in PDB 6X18, the same distance is 22.94 and 22.08 Å, respectively. The wider TM6 opening in the isolated TMD of A316T was however not observed when A316T was in complex with GLP-1, suggesting that the agonist can modulate the TM3-TM5 distance by influencing the rotameric state of T316 and in turn the formation of the Y242^3.45^-T316^5.46^ H-bond.

## DISCUSSION

GPCRs are well known to harbor SNPs, leading to phenotypic variability among individuals and potentially affecting pharmacological responses and disease traits (*68*). Despite the importance of GLP-1R as a major T2D and obesity drug target, and the known variability of responses to GLP-1RAs among individuals taking this drug class (*69*), there is unexpectedly little research on the functional consequences of *GLP1R* genetic variants associated with glycemic traits and human metabolic diseases, particularly in vivo. Although most *GLP1R* variants are low-frequency mutations, understanding their phenotypes and associated pathophysiological changes in humans is valuable as a basis for the development of personalized therapeutic strategies, especially when considering the large number of individuals affected by diseases than can be treated with pharmacological GLP-1RAs, as well as to model conformational effects to be mimicked or avoided when developing novel GLP-1RAs. Our study has attempted to bridge this gap by using a CRISPR-Cas9–based approach to generating a humanized knock-in mouse line bearing the A316T mutation in the human *GLP1R* locus in conjunction with in silico, ex vivo, and in vitro approaches to understanding the association of the rs10305492 G>A variant with T2D protection. Although this missense variant has been previously studied in vitro, analyses using HEK293T, Chinese hamster ovary, or INS-1 cell lines (*25*, *28*, *30*, *70*) have not provided a comprehensive mechanistic explanation for its functional effects, and no data are available in primary tissues or in vivo. Phenotypes such as potential changes in islet cytoarchitecture, as unveiled here, or specific variant effects under metabolic stress or T2D conditions cannot be modeled in vitro, and GWAS studies might not reflect genetic variant effects in individuals with prevalent disease as this is often tested in individuals of “normal” physiology (*71*).

Here, our in vivo A316T model exhibits improved oral glucose tolerance under both chow and HFHS diet conditions, suggesting enhanced sensitivity of the mutant receptor to endogenous GLP-1 despite a tendency for reduced plasma GLP-1 levels in these mice, a phenotype not previously identified in GWAS studies that correlates with a higher degree of ECD opening in response to this agonist for the A316T compared to the WT receptor, and a specific trafficking profile of GLP-1–stimulated A316T not present in response to exendin-4–based GLP-1RAs, involving increased internalization and reduced recycling of the variant receptor when stimulated with the physiological agonist.

A316T-expressing mice also present with significantly lower fasting glucose levels in the chow-fed state, this time in agreement with GWAS reported effects (*23*–*25*). The variant exhibits slower weight gain in diet-induced obese mice, suggesting a protective effect of the A316T mutation for the development of obesity, detectable here despite the reduced level of GLP-1R expression in our humanized *GLP1R* mouse model versus WT mice expressing the endogenous mouse *Glp1r* gene, which is likely to reduce the overall effect of GLP-1R signaling in appetite control in human *GLP1R* mice. We therefore anticipate that the effect of the A316T variant in weight and appetite regulation will be even greater when examined in organisms expressing normal levels of GLP-1R, with the brain action of this GLP-1R mutant warranting additional investigations outside the scope of the present study, potentially extending into the study of neuropsychiatric effects of the GLP-1R (*72*, *73*), including in eating disorders (*74*). Further investigations using the A316T model should also include the examination of the effect of this variant in cardioprotection (*31*), which might help pinpoint the site and mode of action of GLP-1RAs in restoring cardiovascular health.

Despite the positive effect of the A316T variant on glucose homeostasis and endogenous GLP-1 responses, it also presented with blunted responses to several pharmacological GLP-1RAs, indicative of pharmacological incretin resistance. Not all agonists were equally affected, however, with exendin-4 and semaglutide responses being particularly hampered, to the point of causing an apparent increase in blood glucose levels versus vehicle exposure at prolonged time points, suggesting a potentially detrimental impact on glucoregulation under those conditions, while exendin-F1, a Gα_s_-biased analog of exendin-4, retained some beneficial effect 6 hours postagonist administration despite an initial acute loss in chow-fed A316T mice. The in vivo reduction in blood glucose levels under vehicle administration correlates with increases in plasma insulin in both lean and obese A316T mice under unstimulated conditions, likely due to the increased constitutive activity of GLP-1R A316T, which mimics agonist-induced effects in the pancreas and either precludes further insulin secretion increases or triggers excessive desensitization following GLP-1RA exposure. We have also established the clinical relevance of our observations, as heterozygous *hGLP1R*^A316T/+^ mice, the most likely presentation of this variant due to its low frequency in human populations, presented with blunted responses to semaglutide in HFHS-fed diabetic conditions.

In addition to the in vivo study, we have also performed ex vivo analyses of the effects of the A316T variant on islet gene expression, morphology, and α versus β cell mass. While effects were relatively mild in islets from chow-fed mice, we observed increased islet diameter and a notable accumulation of α cells in *hGLP1R*^A316T/A316T^ islets following HFHS feeding. Under this metabolic stress condition, α cells were unusually distributed across the islet core instead of being restricted to the islet mantle. Furthermore, we observed the existence of bihormonal cells expressing both insulin and glucagon, the presence of which has been sometimes associated with human islet exposure to GLP-1RA treatment (*75*, *76*). This observation has led to the unveiling of a liver phenotype following HFHS feeding of the A316T variant–expressing mice. In this context, α cell hyperplasia is often the result of hyperaminoacidemia due to failure of the liver to catabolize amino acids secondary to glucagon resistance (*45*). Accordingly, we have measured a significant increase in plasma amino acid levels as well as a decrease in glucagon secretion at low glucose concentrations in HFHS-fed mice expressing the GLP-1R A316T variant. This evidence indicates that continuous activity from the GLP-1R leads to a glucagon resistance phenotype due to sustained loss of glucagon secretion, a well-known effect of GLP-1R action (*44*), potentially also contributing to the low fasting glucose phenotype and episodes of hypoglycemia reported in carriers of the A316T variant (*23*).

Given the close relationship between endocrine dysregulation and hepatic steatosis (*77*), we anticipated changes in liver fat accumulation in A316T versus WT mice under prolonged HFHS diet. Glucagon deficiency has previously been linked to increased hepatosteatosis (*78*), and both GLP-1RAs (*79*) and GLP-1R/glucagon receptor (GCGR) dual agonists (*80*) have shown promise in the treatment of MASLD. The mechanism of action of each receptor in reducing liver fat is likely to be specific, with GCGR predominantly expressed in hepatocytes and directly improving mitochondrial function and energy expenditure in these cells (*81*), while GLP-1R signaling is linked to reduced glucose levels and improved insulin sensitivity via increased glucose-dependent insulin secretion and weight control, as also seen in this study. In our case, constitutive expression of the GLP-1R A316T variant leads to a shift in liver lipid droplet morphology from a predominantly macrovesicular to a microvesicular phenotype, suggesting hepatocyte mitochondrial dysfunction despite the improved glucose tolerance and tendency for improved insulin tolerance of these mice, a phenotype likely associated to a chronic reduction in α cell glucagon outputs. Our results predict the likely improved profiles of dual GLP-1R/GCGR agonists versus GLP-1R mono-agonists for MASLD treatment, with the former overriding potential loses in glucagon secretion associated with GLP-1R action.

Despite the changes in islet morphology and endocrine cell type observed in this study, we could only detect increased expression of the β cell–enriched gene *MafA*, with a tendency for increased *Ins1* and *Gcg* expression in HFHS-fed *hGLP1R*^A316T/A316T^ mouse islets. Our limited profiling however does not allow the determination of cell type–specific transcriptional profiles and might potentially have been underpowered. Further determination of transcriptional signatures would be required to clarify the endocrine cell identify changes observed in HFHS-fed A316T mouse islets.

To further understand the mechanisms underlying the changes in glucoregulation observed with the GLP-1R A316T variant, we have assessed receptor cell surface expression, cAMP production, and insulin secretion levels in *hGLP1R*^+/+^ versus ^A316T/A316T^ islets. In agreement with previous in vitro observations (*28*, *30*), we detected a significant reduction in cell surface expression of the A316T variant, both ex vivo and in vivo by injecting the LUXendin645 probe into living mice. This reduced surface expression of the A316T receptor has been corroborated throughout this study in all systems tested, including human EndoC-βH3 *GLP1R*^−/−^ cells, human islets, and islets from *hGLP1R*^−/−^ mice transduced with adenoviral vectors expressing pAV-SNAP/FLAG-*hGLP1R*^WT^ or ^A316T^, as well as in INS-1 832/3 *Glp1r*^−/−^ cells stably expressing the WT or A316T human GLP-1R variant, despite receptor overexpression in some of these models. We have also established that this effect is due to an increased rate of receptor turnover, involving increased rates of endocytosis and lysosomal targeting of the GLP-1R A316T variant under basal (vehicle) conditions, in keeping with a higher degree of constitutive activity conferred by the A316T mutation, rather than involving changes in transcription or biosynthetic delivery to the plasma membrane. Further indications of the increased basal activity of GLP-1R A316T obtained in this study include increased levels of basal cAMP, basal intracellular Ca^2+^ rises, and mini-Gs recruitment under vehicle conditions, as well as increased constitutive receptor recruitment to lipid rafts, reduced basal levels of receptor plasma membrane diffusion, and an overall increase in binding to protein interactors under unstimulated conditions, as shown in the mass spectrometry interactome analysis.

In this study, we have also measured enhanced levels of glucose-stimulated insulin secretion for the A316T variant in various ex vivo and in vitro human and mouse β cell models tested, which, in agreement with our in vivo results, did not translate to further increases in insulin secretion in response to GLP-1RAs, blunting the effect of these compounds over vehicle alone responses. Prolonged exendin-4 responses were particularly affected, a result that correlates with the in vivo loss of glucoregulatory benefit for this agonist 6 hours postadministration. This effect further correlates with other indicators of poor signaling responses or increased desensitization in response to exendin-4 for the A316T variant, including reduced receptor segregation to lipid rafts, reduced endosomal activity, increased receptor ubiquitination, and a tendency for reduced exendin-4–induced cAMP generation versus WT GLP-1Rs.

Conversely, cAMP generation or intracellular Ca^2+^ mobilization in response to the Gα_s_-biased analog exendin-F1 was improved in A316T compared to WT GLP-1R–expressing islets, which correlates with a milder impairment in glucoregulation, particularly at prolonged time points, with this GLP-1RA. We hypothesize that this effect might be linked to the favorable signaling profile of this Gα_s_-biased GLP-1RA, which presents with an extra bias toward Gα_s_ versus β-arrestin 2 at the A316T compared to the WT receptor, as shown in the present study, further reducing its propensity to trigger increased desensitization of the variant receptor. This characteristic would enable exendin-F1 to partly counteract the accelerated desensitization of the A316T variant receptor, a feature that instead would be exacerbated by a fast-desensitizing agonist such as exendin-4 (*33*). Exendin-F1 might also enable the A316T receptor to adopt a more favorable conformation for signal transduction, as highlighted here by the different local dynamics imprinted by this agonist in our MD simulations.

Further insights are provided by the cryo-EM structure of the GLP-1R A316T variant in complex with Gα_s_ and GLP-1, showing a consensus atomic model similar to that of the WT GLP-1R but with subtle changes to interactions between TM3, TM5, and TM6. Of note, we were only able to determine the cryo-EM structure of the A316T receptor with a relatively modest (3.3 Å) resolution, a result that we attribute to potentially higher flexibility or dynamics of the A316T compared to the WT receptor. It has previously been proposed, using the x-ray crystal structure of exendin-4–bound GLP-1R as a model, that the A316T variant might disrupt H-bonding between N320^5.50^ and E364^6.53^ by the formation of a direct interaction between E364^6.53^ and T316 (*23*). However, the cryo-EM structure of the GLP-1–bound A316T receptor obtained in this study does not exhibit these structural changes, instead suggesting, as supported by our MD simulations, the potential disruption of central polar network residues via several subtle alterations in the interhelical water network bridging TM5 and TM6. In addition, consistently with the mechanism suggested by the MD simulations, the GLP-1–bound GLP-1R A316T cryo-EM structure offers support for the disruption of interactions between Y242^3.45^ and P312^5.42^ in favor of the Y242^3.45^-T316^5.46^ H-bond, selective for a specific T316^5.46^ rotameric state. 3DVA of the GLP-1R A316T structure also suggests greater mobility in the extracellular loops of the variant receptor, particularly in ECL3, compared to the WT receptor bound to the same agonist (*82*, *83*). If this region is truly more dynamic in the A316T variant, then it is conceivable that GLP-1 peptide cycling (disengagement and reengagement) with the receptor could be faster, leading to more signaling events per unit time than for the WT GLP-1R. These observations are subject to certain limitations related to the abovementioned modest resolution of the A316T variant structure, which allows for confident placement of the receptor TMD side chains but is below the resolution required to place structural waters implicated in the proposed mechanism of constitutive activity for this variant. To overcome this limitation, we have used MD simulations which are a useful tool to study water molecules within biological systems with good reliability (*84*). Here, they aided in predicting a water network involved in the communication between TM5 and TM6 of A316T, possibly contributing to the unique pharmacology of A316T. Furthermore, the simulations also suggested an allosteric mechanism triggered by Y242^3.45^-T316^5.46^ H-bond, transmitted to the intracellular side of the receptor by altering the mobility of TM6 in the apo-state A316T. However, as there are no other published structures of known constitutively active GLP-1R mutants, it is difficult to conclude whether the differences in water network interactions and extracellular loop dynamics unveiled in the present structure are sufficient to fully account for the constitutively increased activity of A316T. Our findings additionally suggest that the main source of this enhanced activity is the receptor itself rather than being triggered by ligand binding, despite the different modes of engagement of the variant receptor with specific GLP-1RAs such as exendin-F1. Apo-state structures for both the WT and the A316T receptors would need to be obtained to fully elucidate structural changes in the absence of bound agonist.

Despite these limitations, when taken together, our MD simulations aligned well with the cryo-EM data and are consistent with a concerted mechanism behind the enhanced constitutive A316T activity which is triggered by the bulkier, more polar T316 side chain. The structural model informed by the cryo-EM and MD simulations data for the effect of the Ala to Thr substitution in position 316 (fig. S10E) suggests changes to interactions between TM3 and TM5 triggered at position 316 that are propagated to the intracellular side of GLP-1R. In particular, a stabilizing effect of water molecules on the TM6 kink, in concert with the tighter helical packing between TM3 and TM5, aids to the intracellular opening movement of TM6, pivotal for Gα_s_ recruitment and activation. In physiological conditions, this allosteric effect would move the inactive-active A316T equilibrium toward the active state, favoring the constitutive activation of the receptor and the increased basal Gα_s_ coupling observed in our functional experiments.

To conclude, we present here a comprehensive assessment of the effects of the A316T missense mutation in human and murine pancreatic β cells, primary islets, liver, and in vivo using a genetically engineered humanized knock-in mouse model, including assessment of both agonist-dependent and agonist-independent responses. We demonstrate in vivo increased basal but reduced GLP-1RA–dependent glucoregulatory effects, with parallel changes present for islet insulin secretion, correlating with changes in ex vivo and in vitro signaling responses. Our study analyzes in detail the agonist-dependent and agonist-independent trafficking and signaling profiles of this variant in physiologically relevant β cell systems, confirming some results from previous in vitro and GWAS studies, including a marked GoF phenotype for the A316T missense mutation leading to increased basal desensitization and turnover and resulting in reduced cell surface expression of the mutant receptor, which can be rescued by lysosomal inhibition. We also observe varying responses to stimulation with different GLP-1RAs, with Gα_s_-biased agonists such as exendin-F1 associated with a more favorable signaling profile compared to balanced agonists such as exendin-4 and semaglutide (see table S5 for a summary of the main results of the study). These data have important implications for the design of future GLP-1RAs and for precision medicine approaches, whereby structural residue-residue interactions could be emulated to achieve allosteric effects similar to those present in the A316T variant, or individuals could be prescribed selected GLP-1RAs based on their specific *GLP1R* genetic profile. Our study also serves as a model for future studies to be carried out with other gene variants of the *GLP1R* and related receptors which are the target of antidiabetic and anti-obesity treatments and highlights the utility of the A316T model for the identification and analysis of extra-pancreatic effects of the GLP-1R.

## MATERIALS AND METHODS

### Peptides, plasmids, and reagents

GLP-1(7-36)NH_2_ and exendin (*9*–*39*) (exendin-9) were purchased from Bachem; semaglutide was obtained from Imperial College London Healthcare NHS Trust pharmacy; exendin-4 and exendin-F1 were custom synthesized by Wuxi AppTec/Insight Biotechnology and were at least 95% pure. Tetramethylrhodamine (TMR)–labeled exendin-9 and exendin-4 (exendin-9–TMR and exendin-4–TMR) have been described and validated before (*85*, *86*). Exendin-9–Cy5 (LUXendin645) has been described before (*87*) and is a gift from D. Hodson (Oxford Centre for Diabetes, Endocrinology and Metabolism, UK). SNAP-Surface fluorescent probes were purchased from New England Biolabs. Surface cleavable BG-S-S-649 (*59*, *88*, *89*) was a gift of I. Corrêa Jr, New England Biolabs. LgBiT-mini-G_s_ (*90*) was a gift from N. Lambert (Augusta University, USA). LgBiT–β-arrestin 2 (*61*, *91*) was purchased from Promega. pSNAP/FLAG-tagged human *GLP1R* (*hGLP1R*)^A316T^ and *hGLP1R*^A316T^-SmBiT constructs were generated in house by site-directed mutagenesis from pSNAP/FLAG-*hGLP1R*^WT^ (Revvity) and *hGLP1R*^WT^-SmBiT (*61*) with the following primers: 5′-CCGGCTGCCCATTCTCTTTACCATTGGGG-3′ (forward) and 5′-ACCCCAATGGTAAAGAGAATGGGCAGCCG-3′ (reverse) for pSNAP/FLAG-*hGLP1R*^A316T^; 5′-CCGGC-TGCCCATTCTCTTTACCATTGGGG-3′ (forward) and 5′-ACCCCAATGGTAAAGAGAATGGGCAGCCG-3′ (reverse) for *hGLP1R*^A316T^-SmBiT, using the QuikChange Site-Directed Mutagenesis Kit (Agilent) following the manufacturer’s instructions.

### Animal studies

All animal procedures were approved by the British Home Office under the UK animals (Scientific Procedures) Act 1986 (Project License number PP7151519 to A. Martinez-Sanchez, Imperial College London, UK) and from the local ethical committee (Animal Welfare and Ethics Review Board) at the Central Biological Services unit of Imperial College London. Animals were housed in groups of up to four adult mice in individually ventilated cages under controlled conditions at 21° to 23°C with 12-hour:12-hour light-to-dark cycles (lights on at 07:00). Ad libitum access to water and feed was provided unless otherwise stated. Lean mice were fed a standard chow diet [RM1 (E); Special Diet Services]. For HFHS diet studies, animals were put on a 58 kcal % fat and sucrose diet (D12331, Research Diets Inc.) ad libitum for 10 to 14 weeks before experiments.

### Generation of *hGLP1R*^A316T/A316T^ and *hGLP1R*^−/−^ mice

Transgenic mice were generated on a C57BL/6 *hGLP-1R^+/+^* mouse background from Taconic Artemis GmbH that has been described before (*32*). To generate mice with the *hGLP1R* A316T mutation, one-cell stage *hGLP1R*^+/+^ embryos were electroporated with 100 μl of Opti-MEM (Thermo Fisher Scientific) containing ribonucleoprotein complexes of *Streptococcus pyogenes* Cas9 (1.2 μM Alt-R *S.p.* Cas9 nuclease 3NLS) with 6 μM single guide RNA (sgRNA) and donor single-stranded repair oligo DNA nucleotides (ssODNs; 300 ng/μl) using the NEPA21 electroporator (NEPA GENE Co. Ltd.). DNA nucleotides, *S.p.* Cas9, nuclease and sgRNA were purchased from Integrated DNA Technologies. The sgRNA used to introduce the Ala^316^ to Thr mutation was 5′-GCCCATTCTCTTTGCCATTG-3′, designed using RGEN tool (http://rgenome.net/cas-designer/), targeting a sequence in exon 9 of *hGLP1R* flanking the rs10305492 SNP. The donor ssODNs in this study are as follows: 5′-CATGAACTACTGGCTCATTATCCGGCTGCCCATTCTCTTTACCATTGGGGTGAACTTCCTCATCTTTGTTCGGGTCATCTGCAT-3′ (forward) and 3′-ATGCAGATGACCCGAACAAAGATGA-GGAAGTTCACCCCAATGGTAAAGAGAATGGGCAGCCGGATAATGAGCCAGTAGTTCATG-5′ (reverse), designed using the Alt-R CRISPR HDR design tool (https://eu.idtdna.com/pages/tools/alt-r-crispr-hdr-design-tool/) with the same target sequence as for the sgRNA (fig. S1A). Following electroporation, two-cell stage embryos were placed into oviducts of pseudo-pregnant *hGLP1R*^+/+^ foster mothers to generate the F0 founder mice, which were subsequently screened for the presence of the A316T substitution as described below (fig. S1B). Mice with frameshift disruptions of *hGLP1R* were also selected from F0 to establish a *hGLP1R*^−/−^ mouse colony.

### Genomic DNA extraction and genotyping

To genotype the transgenic mice, ear samples were collected from weaned animals and genomic DNA extracted in alkaline lysis buffer [25 mM NaOH and 0.2 mM EDTA (pH 8.0)] for 1 hour at 95°C and subsequently neutralized by addition of 13 mM tris-HCl (pH 7.4) (*61*). The purified genomic DNA was used as a template for PCR with appropriate primers before Sanger sequencing to identify the A316T mutation. The genotyping primers are described in table S6.

### OGTTs, IPITTs, and IPGTTs

For oral glucose tolerance tests (OGTTs), chow- or HFHS-fed *hGLP1R*^A316T/A316T^ mice and littermate controls were fasted for 5 hours, and blood glucose levels were serially assessed at 0, 10, 30, and 60 min via tail venipuncture using a handheld glucometer (Accu-Chek) after administration of a glucose challenge (2 g/kg of body weight) into the gut by oral gavage.

For IPITTs, chow- or HFHS-fed *hGLP1R*^+/+^ and *hGLP1R*^A316T/A316T^ mice were fasted for 5 hours, and basal glucose was measured as above before intraperitoneal injection of insulin (0.75 IU/kg of body weight) and blood glucose level assessment at 15, 30, and 60 min postinsulin injection.

To examine acute and prolonged glycemic responses to GLP-1RAs, animals were fasted for 2 hours, starting at 8 a.m. on the morning of the experiments, before IPGTTs. GLP-1RAs were then coadministered at the indicated concentrations with 20% (w/v) d-glucose via intraperitoneal injection at weight-adjusted volumes. Baseline glucose readings were taken via tail venipuncture, and subsequent readings were taken at various time points after glucose ± GLP-1RA administration as for the OGTTs (0-hour acute IPGTTs). For GLP-1RAs with shorter half-lives (exendin-4 and exendin-F1), IPGTTs were repeated 6 hours after the start of the initial IPGTT, with continued fasting. For the extended half-life GLP-1RA semaglutide, IPGTTs were performed 24 and 72 hours after GLP-1RA administration, with access to food restored at the end of each IPGTT. The in vivo IPGTT studies were performed with a crossover design, with each mouse receiving vehicle and each GLP-1RA treatment with washout periods of at least 1 week between the different GLP-1RA treatments.

### Measurement of plasma insulin, glucagon, amino acid, and GLP-1 levels

To measure insulin release during IPGTTs, 10 μl of blood samples were collected via tail venipuncture 10 min after a glucose challenge. Plasma glucagon and GLP-1 samples (20 μl) were similarly collected from mice following 5 hours fasting (glucagon) or 10 min after oral glucose administration during OGTTs (GLP-1) and stored in the presence of DPP-4 inhibitors to avoid degradation (GLP-1).

Plasma was collected into potassium EDTA cuvettes (Microvette CB 300, 16.444.100, Starstedt) and stored on ice before centrifugation at 8,000*g* for 10 min at 4°C to collect plasma supernatants in fresh Eppendorf tubes kept at −80°C until further analysis. Plasma insulin was measured using the Mouse Plasma Insulin Homogeneous Time-Resolved Fluorescence (HTRF) kit (#62IN3PEF; Revvity); plasma glucagon was measured using the Mouse Glucagon ELISA Kit (#81518, Crystal Chem); plasma GLP-1 was measured using an enzyme-linked immunosorbent assay (ELISA) kit (#A145866, antibodies.com); and total amino acid levels were assessed from plasma insulin samples using an amino acid assay kit (#A319661, antibodies.com), following the manufacturers’ instructions.

### Pancreas and liver section preparation and labeling

Whole pancreata and livers from *hGLP1R*^A316T/A316T^ mice and *hGLP1R*^+/+^ control littermates were dissected, placed in ice-cold phosphate-buffered saline (PBS), cleared from surrounding tissues, weighed, and fixed for 24 hours in 4% paraformaldehyde (PFA) at 4°C and then cryoprotected in 30% sucrose, followed by embedding in optical cutting temperature and storing at −80°C. Frozen 8- to 10-μm sections were prepared using a cryostat.

Pancreas sections were immunostained with guinea pig anti-insulin polyclonal antibody (1:100; Dako IR002, Agilent Technologies; Alexa Fluor 488 secondary antibody, Thermo Fisher Scientific) for β cell labeling, mouse anti-glucagon antibody (1:500; G2654, Sigma-Aldrich; Alexa Fluor 568 secondary antibody, Thermo Fisher Scientific) for α cell labeling, and rabbit anti-Ki67 antibody (1:2000; A104333, antibodies.com; Alexa Fluor 647 secondary antibody, Thermo Fisher Scientific) as a marker of proliferation. Samples were mounted with Prolong Diamond Antifade Mountant with 4′,6-diamidino-2-phenylindole (DAPI; P36962, Thermo Fisher Scientific) for nuclei visualization. Stained slides were imaged at 4× to quantify total pancreatic area and at 40× to image pancreatic islet insulin and glucagon-positive areas using a wide-field Zeiss Axio Observer inverted microscope from Imperial College Facility for Imaging by Light Microscopy (FILM). Total pancreatic, insulin, and glucagon-positive areas were measured per slide using the Fiji software, and β and α cell mass was calculated by normalizing to total pancreatic section area and pancreas weight as described before (*92*, *93*). Islet size and cytoarchitecture analyses were performed on the same images used for β and α cell mass assessments. All non-islet regions were erased using the ImageJ outline and clear outside functions. Cells were assigned to be in the islet periphery/mantle if they were within the two outermost cell layers of the islet or in the islet core if they fell within any layers deeper than these.

Liver sections were stained with 2 μM BODIPY 493/503 (4,4-difluoro-1,3,5,7,8-pentamethyl-4-bora-3a,4a-diaza-s-indacene) (Thermo Fisher Scientific) in PBS for 15 min, washed three times with room temperature PBS, and mounted with Prolong Diamond Antifade Mountant with DAPI as above. Sections were imaged with a 20× objective in a Nikon Eclipse Ti microscope with an ORCA-Flash 4.0 camera (Hamamatsu) and Metamorph software (Molecular Devices). The number of lipid droplets per cell and the average size of lipid droplets per section were quantified using the 3D Objects Counter function in Fiji.

### In vivo pancreatic hGLP-1R labeling

The *hGLP1R*^A316T/A316T^ mice and *hGLP1R*^+/+^ control littermates were injected subcutaneously with exendin-9–Cy5 (100 pmol/g) 2 hours before terminal anesthesia (1.5% sodium pentobarbital) and transcardial perfusion fixation with PBS at pH 7.2, supplemented with 4% fresh PFA (*2*). Pancreata were dissected, and frozen sections were prepared and mounted as above. Images of sections were captured using a Nikon Eclipse Ti spinning disk confocal microscope and 60× oil immersion objective with an ORCA-Flash 4.0 camera (Hamamatsu) and Metamorph software (Molecular Devices), with λ_ex_ = 660 nm and fluorescent intensity in islet areas normalized by the number of islet cell nuclei calculated in Fiji.

### Isolation and maintenance of islets

The appropriate animals were humanely euthanized before pancreas inflation via injection of RPMI 1640 medium (S1745602, Nordmark Biochemicals) supplemented with collagenase (1 mg/ml) from *Clostridium histolyticum* (S1745602, Nordmark Biochemicals) into the common bile duct. The pancreases were then dissected and incubated in a water bath at 37°C for 10 min. Digestion was quenched with cold mouse islet culture medium: RPMI 1640 supplemented with 10% (v/v) fetal bovine serum (FBS) (F7524, Sigma-Aldrich) and 1% (v/v) penicillin/streptomycin (P/S) (15070-063, Invitrogen). Islets were subsequently washed, purified on a Histopaque gradient (Histopaque-1119 and Histopaque-1083, Sigma-Aldrich), and allowed to recover overnight at 37°C in 5% CO_2_ in mouse islet culture medium before any experiments. Human donor islets from deceased heart-beating donors of both sexes were provided by the European Diabetes Study Centre, Strasbourg, France. Human pancreatic tissue was collected with consent from next of kin where required, following the French bioethics legislation and INSERM guidelines (French bylaw, published on 29 December 1998), and national and ethical approvals in place. Human islets were cultured in RPMI 1640 supplemented with l-glutamine, 10% (v/v) FBS, 1% (v/v) P/S, amphotericin B (0.25 μg/ml), and 5.5 mM glucose at 37°C in 5% CO_2_. Human donor characteristics are listed in table S7.

### Cell line generation and culture

INS-1 832/3 cells stably expressing pSNAP/FLAG-*hGLP1R*^WT^ or ^A316T^ were derived in house from the parental INS-1 832/3 *Glp1r*^−/−^ cell line, where endogenous rat GLP-1R has been eliminated by CRISPR-Cas9 (*55*), a gift from J. Naylor, AstraZeneca, by transfecting the corresponding plasmid followed by selection in G418 (1 mg/ml) and fluorescence-activated cell sorting (FACS) of the SNAP-positive cell population, and maintained in RPMI 1640 with 11 mM glucose, 10 mM Hepes, 2 mM glutamine, 1 mM sodium pyruvate, 50 μM β-mercaptoethanol, 10% FBS, 1% P/S, and G418 (1 mg/ml). Human EndoC-βH3 cells (Human Cell Design) were cultured on fibronectin (2 μg/ml) and 1% extracellular matrix (E1270, Sigma-Aldrich)–coated plates in Advance Dulbecco’s modified Eagle’s medium/F-12 (Thermo Fisher Scientific), sodium selenite (6.7 ng/ml), 10 mM nicotinamide, human transferrin (5.5 μg/ml), 2 mM l-glutamine, 2% BSA Fraction V (Roche), 1% P/S solution, and 50 μM β-mercaptoethanol. All cells were incubated in a humidified air incubator containing 5% CO_2_ at 37°C.

#### *Generation and characterization of EndoC-*β*H3 GLP1R^−/−^-enriched cells*

*CRISPR-Cas9 dgRNA design*. To generate EndoC-βH3 *GLP1R*^−/−^ cells, a lentiviral CRISPR-Cas9 approach was used as previously described (*94*). Cas-Designer (http://rgenome.net/cas-designer/) was used to design two 17 nucleotide-long (truncated) guide RNAs (gRNAs), 5′-GCTGCTCGGGATGGTGGGCA-3′ and 5′-GTTGCAGAACAAGTCTGTGG-3′ (reverse complement), flanking the target DNA sequences. Designed gRNAs were cloned into a pSpCas9(BB)-2A-Blast backbone (plasmid #118055, Addgene), which allows for blasticidin selection of cells after transfection, following a previously described dual gRNA cloning strategy (*94*) as follows: Oligonucleotides corresponding to deletion-yielding pairs of gRNAs were assembled in a reaction mix containing 1× Q5 reaction buffer, 200 μM deoxynucleotide triphosphates, 0.5 μM primer, Q5 high-fidelity DNA polymerase (0.02 U/μl), and pScaffold-H1 vector (0.25 ng/μl; plasmid #118152, Addgene), with annealing temperature (Ta) = 58°C, 15-s extension, for 30 amplification cycles. Polymerase chain reaction (PCR) products were digested and ligated to pSpCas9(BB)-2A-Blast in a one-step digestion ligation reaction with 1× Tango buffer, 1 μl of FastDigest BbsI (Thermo Fisher Scientific), 1 μl of 0.1 M dithiothreitol (DTT), 1 μl of 10 mM adenosine triphosphate, 0.5 μl of T7 ligase, 100 ng of vector, and 1 μl of PCR product (diluted 1:20). Digestion-ligation reactions underwent 6 cycles of 5 min at 37°C and 5 min at 23°C, and a final 5-min incubation at 37°C, after which 1 μl of ligation product was transformed into NEB Stable competent *Escherichia coli* (New England Biolabs). Successful double gRNA (dgRNA) insertion was confirmed by Sanger sequencing.

*Lentiviral production and transduction of EndoC-*β*H3 cells.* To produce CRISPR-Cas9 lentiviral particles, HEK293T cells were cotransfected with dgRNA vector from above as well as packaging (psPAX2) and envelope (pMD2.G) vectors using a calcium phosphate protocol (*95*). Viral supernatants were harvested 48 and 72 hours after transfection, cleared using a 0.45-μm Millex-HV filter, and concentrated by adding PEG 8000 (Sigma-Aldrich) and NaCl until final concentrations of 10% and 0.15 M, respectively. The mixture was then incubated for 16 to 20 hours on a shaker at 4°C, followed by centrifugation for 30 min at 10,000*g* at 4°C. Virus pellets were resuspended in 100 μl of PBS and stored at −80°C. Forty-eight hours before transduction, EndoC-βH3 cells were seeded at 1 million cells per well in six-well plates, and 50 μl of virus was used to infect each well. After 72 hours, cells were selected for vector integration using blasticidin (20 μg/ml) for 10 days, with selection medium changed every ~3 days (fig. S1C). After selection, cells were labeled with fluorescent exendin-9–TMR for 10 min and reverse FACS sorted to recover the population enriched in nonlabeled *GLP1R*^−/−^ cells.

*EndoC-βH3 GLP1R^−/−^ validation by HTRF cAMP assay.* EndoC-βH3 WT and *GLP1R*^−/−^ cells were pretreated for 3 weeks with 1 μM 4-hydroxytamoxifen to induce β cell differentiation (*54*). On the day of the experiment, cells were incubated in 0.5 mM glucose containing EndoC-βH3 medium ± 100 nM exendin-4 and the phosphodiesterase inhibitor isobutyl methylxanthine (IBMX). Cells were then lysed in Krebs-Ringer bicarbonate-Hepes (KRBH) [140 mM NaCl, 3.6 mM KCl, 1.5 mM CaCl_2_, 0.5 mM MgSO_4_, 0.5 mM NaH_2_PO_4_, 2 mM NaHCO_3_, and 10 mM Hepes, saturated with 95% O_2_/5% CO_2_; pH 7.4] buffer + 1% Triton X-100, and lysates were used to determine cAMP production using a cAMP Dynamic 2 HTRF-based assay kit (Revvity) in a PHERAstar reader (BMG Labtech), following the manufacturer’s instructions. Results show decreased cAMP responses in EndoC-βH3 *GLP1R^−/−^* versus WT cells (fig. S1D), suggesting a partial enrichment in *GLP1R^−/−^* within the CRISPR-Cas9–transduced cell population.

### Insulin secretion assays

Purified mouse islets used for insulin secretion assays were treated in 24-well nonadherent plates. Eight islets were used per replicate with three technical replicates per condition. For acute studies, islets were preincubated for 1 hour in KRBH buffer containing 0.1% (w/v) bovine serum albumin (BSA) (10775835001, Roche) and 3 mM glucose before incubation with 11 mM glucose ± 100 nM GLP-1RAs or the GLP-1R antagonist exendin-9 in KRBH buffer in a shaking 37°C water bath (80 rpm) for 1 hour. For overnight studies, preincubation was carried out in RPMI 1640 medium containing FBS, P/S, and 3 mM glucose, followed by treatment with medium supplemented with 11 mM glucose ± GLP-1RAs for 16 hours. Supernatants containing secreted insulin were collected, centrifuged at 1000 rpm for 1 min, and transferred to fresh tubes. For total insulin content, islets were lysed using acidic ethanol (75% ethanol, 1.5 mM HCl). Lysates were sonicated 3× 10 s each in a water bath and centrifuged at 10,000*g* for 10 min, and supernatants were collected. For human islets, the same experiment was performed with islets previously transduced with pAV-SNAP/FLAG-*hGLP1R*^WT^ or ^A316T^ adenoviruses (generated by VectorBuilder) at a multiplicity of infection of 1, 24 hours before the start of experiments. Insulin secretion assays with the human EndoC-βH3 cell line were performed following 3 weeks of treatment with 4-hydroxytamoxifen as above, followed by transduction with pAV-SNAP/FLAG-*hGLP1R*^WT^ or ^A316T^ adenoviruses, with insulin secretion assessed as previously described (*54*), with the following modifications: After preincubation with 0.5 mM glucose KRBH buffer, cells were stimulated with 15 mM glucose ± exendin-4 for 60 min. Total and secreted insulin samples from islets and cells were stored at −20°C until quantification using an Insulin Ultra-Sensitive HTRF kit (#62IN2PEG, Revvity) in a PHERAstar plate reader (BMG Labtech), according to the manufacturer’s instructions.

### Glucagon secretion assays

Islets were preincubated as above for 1 hour in KRBH buffer containing 0.1% (w/v) BSA supplemented with 11 mM glucose before incubation with 0.5 mM glucose in KRBH in a shaking 37°C water bath (80 rpm) for 2 hours. Supernatants and islet extracts were collected and processed as above, and secreted and total glucagon levels were assessed as above using the HTRF Glucagon Detection Kit (#62CGLPEG, Revvity) according to the manufacturer’s instructions.

### Real-time cAMP imaging

To measure cAMP, *hGLP1R*^A316T/A316T^ versus ^+/+^ control mouse islets, or INS-1 832/3 SNAP/FLAG-*hGLP1R*^WT^ versus ^A316T^ cells cultured on glass-bottom MatTek dishes, were transduced with cADDis (Green Gs cADDis cAMP Kit, Montana Molecular), a baculovirus genetically encoding a fluorescent cAMP in situ biosensor, following the manufacturer’s instructions. Transduced islets were subsequently encased into Matrigel on glass-bottom MatTek dishes 24 hours after transduction, and both islets and cells were imaged at λ_ex_ = 488 nm in a Nikon Eclipse Ti spinning disk confocal microscope and 20× objective with an ORCA-Flash 4.0 camera (Hamamatsu) for time-lapse recordings with image acquisitions every 6 s in KRBH buffer containing 0.1% (w/v) BSA and 6 mM glucose for 2 min to record the baseline, with 100 nM GLP-1RA subsequently added by manual pipetting, and islets imaged for a further 5 min before addition of 100 μM IBMX + 10 μM forskolin for the final 2 min of the acquisition. Raw green fluorescence intensity traces were extracted from whole islet regions of interest (ROIs) using Fiji, and mean intensities were calculated for each ROI and time point. Responses were plotted relative to average fluorescence intensity per islet during the baseline period before GLP-1RA addition.

### Islet immunofluorescence

For immunostaining, *hGLP1R*^A316T/A316T^ or ^+/+^ control mouse islets were fixed in 4% PFA and stored in PBS. After 15 min of permeabilization with 0.5% (v/v) Triton X-100 in PBS, the islets were washed once with PBS and incubated in blocking buffer (PBS + 1% BSA) for 1 hour. Primary antibodies against insulin (1:50; Dako IR002, Agilent Technologies) and glucagon (1:500; G2654, Sigma-Aldrich) were added overnight in blocking buffer, followed by 3× washes in PBS and 1-hour incubation at room temperature with secondary anti-guinea pig Alexa Fluor 488 and anti-mouse Alexa Fluor 647 (both at 1:500; Thermo Fisher Scientific). Islets were then loaded onto glass-bottom MatTek dishes in PBS, and three images were acquired at different z positions by confocal microscopy in a Zeiss LSM-780 inverted confocal laser scanning microscope and a 63×/1.4 numerical aperture oil immersion objective from Imperial College FILM Facility. Images were analyzed using Fiji.

### Measurement of mini-G_s_ and β-arrestin 2 recruitment by NanoBiT complementation

For mini-G_s_ recruitment, INS-1 832/3 *Glp1r*^−/−^ cells were seeded in 6-cm dishes and transiently cotransfected with 1.7 μg each of *hGLP1R*^WT^ or ^A316T^-SmBiT and LgBiT-mini-G_s_ constructs for 24 hours. Cells were then detached, resuspended in Hanks’ balanced salt solution (HBSS) containing NanoGlo Live Cell Reagent (1:20; Promega) with furimazine, and seeded onto white 96-well half-area plates. Baseline luminescence was immediately recorded over 8 min at 37°C in a Flexstation 3 plate reader (Molecular Devices) and for a further 30 min after GLP-1RA addition at serial doses up to 100 nM. Readings were taken every 30 s and normalized to average well baseline, and average vehicle-induced signal was subtracted to establish the agonist-induced effect. To measure β-arrestin 2 recruitment, cells were processed as above with slight modifications: INS-1 832/3 *Glp1r*^−/−^ cells were cotransfected with 1.77 μg each of *hGLP1R*^WT^ or ^A316T^-SmBiT and LgBiT–β-arrestin 2 plasmids for 24 hours, and GLP-1RAs were added at serial doses up to 1 μM. AUCs were calculated for each agonist concentration and fitted to log(agonist) versus response four-parameter curves using GraphPad Prism 10.1.2, with errors propagated for mini-G_s_ over β-arrestin 2 recruitment bias calculations.

### High content microscopy GLP-1R trafficking assays

#### Internalization assay

INS-1 832/3 *Glp1r*^−/−^ SNAP/FLAG-*hGLP1R*^WT^ or ^A316T^ cells were seeded onto poly-d-lysine–coated black 96-well plates for 24 hours before the assay. Cells were labeled for 30 min with 1 μM SNAP surface cleavable BG-SS-649 probe at 37°C and washed once with HBSS before treatment with GLP-1RA or vehicle for 60, 30, 15, or 5 min at 37°C in complete medium. Cells were then washed with cold HBSS, and subsequent steps were carried out at 4°C. The cell-impermeable reducing agent Mesna {100 mM in alkaline tris-NaCl-EDTA (TNE) buffer [20 mM tris-HCl, 1 mM EDTA, and 150 mM NaCl (pH 8.6)]} or alkaline TNE buffer without Mesna was applied for 5 min to cleave BG-SS-649 probe left at the cell surface and then washed with HBSS. Plates were imaged immediately without fixation using a modular microscope platform from Cairn Research incorporating a Nikon Ti2E, LED light source (CoolLED), fitted with a 20× phase contrast objective, assisted by custom-written high-content analysis software implemented in Micro-Manager. A minimum of nine images per well were acquired for both epifluorescence and transmitted phase contrast. Internalized GLP-1R, indicated by cell-associated fluorescence, was analyzed after flat-field correction of fluorescent images using the BaSiC plugin (*96*), segmentation of cell-containing regions from phase contrast images using Phantast (*97*), and fluorescence intensity quantification. The percentage of internalized receptor was calculated as follows

$$\frac{\left[F_{+\text{Me}}\left(t_{x}\right)/F_{-\text{Me}}\left(t_{x}\right)\right]-\left[F_{+\text{Me}}\left(t_{0}\right)/F_{-\text{Me}}\left(t_{0}\right)\right]}{1-\left[\frac{F_{+\text{Me}}\left(t_{0}\right)}{F_{-\text{Me}}\left(t_{0}\right)}\right]}$$

where *F*_*+*Me_ and *F*_*−*Me_ are mean fluorescence intensities ± Mesna at times *t_x_* (5, 15, 30, or 60 min) or *t*_0_ (0 min). Percentages of internalization were plotted to calculate the AUC using GraphPad Prism 10.1.2.

#### Recycling assay

INS-1 832/3 *Glp1r*^−/−^ SNAP/FLAG-*hGLP1R*^WT^ or ^A316T^ cells were seeded as above for 24 hours, washed with HBSS, pretreated with vehicle or GLP-1RA for 1 hour at 37°C in complete medium, washed again, and treated with 100 nM exendin-4–TMR in reverse time order for 3 hours and 1 hour at 37°C in complete medium. Plates were then promptly imaged after washing as above. In this assay, total exendin-4–TMR uptake is indicative of residual surface receptor at the end of the GLP-1RA treatment and cumulative reappearance of the surface receptor during the recycling period.

#### Degradation assay

INS-1 832/3 *Glp1r*^−/−^ SNAP/FLAG-*hGLP1R*^WT^ or ^A316T^ cells were seeded as above. Cells were then washed with HBSS before addition of cycloheximide (50 μg/ml) in serum-free medium to halt protein translation. After 1 hour, a time course was performed with the GLP-1RA added in reverse time order for 8, 4, 2, 1, 0.5, and 0 hours. For the final 30 min of the experiment, the cell medium was replaced with 1 μM of total SNAP-labeling fluorescent probe BG-OG in complete medium, to label total residual GLP-1R. Cells were then washed three times with HBSS before plate imaging as above using a green fluorescence filter set. Total GLP-1R was quantified as the average cellular fluorescence labeling per well (as defined above), with further subtraction of nonspecific fluorescence (i.e., cells without BG-OG labeling). Results were expressed as a percentage relative to vehicle-treated BG-OG–labeled cells and AUCs recorded as above.

### Lysosomal inhibition assay

INS-1 832/3 *Glp1r*^−/−^ SNAP/FLAG-*hGLP1R*^WT^ or ^A316T^ cells on coverslips, mouse *hGLP1R*^−/−^ islets transduced with pAV-SNAP/FLAG-*hGLP1R*^WT^ or ^A316T^ adenoviruses, or *hGLP1R*^A316T/A316T^ and ^+/+^ control islets were incubated for 2 hours ± 400 nM bafilomycin-A1 (Sigma-Aldrich), an established vacuolar H^+^-ATPase and lysosomal trafficking inhibitor (*98*), in complete medium. Cells and transduced *hGLP1R*^−/−^ islets were then labeled with 1 μM SNAP-Surface 549, and *hGLP1R*^A316T/A316T^ versus ^+/+^ islets were treated with exendin-9–TMR in complete medium at 37°C for 10 min. Cells were washed and fixed in 4% PFA, mounted in Prolong Diamond antifade reagent with DAPI, while islets were loaded onto glass-bottom MatTek dishes in PBS, and images were acquired by confocal microscopy with a Zeiss LSM-780 inverted confocal laser scanning microscope and a 63×/1.4 numerical aperture oil immersion objective from the Imperial College FILM Facility. Images were analyzed using Fiji.

### Surface GLP-1R expression analysis

INS-1 832/3 *Glp1r*^−/−^ SNAP/FLAG-*hGLP1R*^WT^ or ^A316T^ cells or EndoC-βH3 cells transduced with pAV-SNAP/FLAG-*hGLP1R*^WT^ or ^A316T^ adenoviruses were labeled with 1 μM SNAP-Surface 549 at 37°C in 5% CO_2_, washed twice in PBS, and fixed with 4% PFA at 4°C for 20 min, mounted onto slides in Diamond Prolong anti-fade Mountant with DAPI (Thermo Fisher Scientific), and images were acquired by confocal microscopy with the Zeiss LSM-780 inverted confocal laser scanning microscope from above using a 20× objective. Human islets and *hGLP1R*^−/−^ mouse islets transduced with pAV-SNAP/FLAG-*hGLP1R*^WT^ or ^A316T^ adenoviruses were labeled with 1 μM SNAP-Surface 649 for 10 min, washed with PBS, and imaged as above with a 63×/1.4 numerical aperture oil immersion objective. *hGLP1R*^A316T/A316T^ versus ^+/+^ transgenic mouse islets were labeled with a saturating concentration (1 μM) of exendin-9–TMR in complete medium at 37°C for 10 min and imaged as above. Images were analyzed using Fiji.

### RNA extraction and qPCR

Total RNA was extracted from islets (*2*, *28*, *50*–*97*) isolated from *hGLP1R*^A316T/A316T^ versus ^+/+^ mice, using TRIzol reagent (Invitrogen) and briefly vortexed for homogenization. Chloroform was added to achieve phase separation, and the upper aqueous phase was collected. RNA was recovered overnight by precipitation with isopropanol (Thermo Fisher Scientific), and 100 to 500 ng was reverse transcribed using the MultiScribe Reverse Transcriptase (Thermo Fisher Scientific) according to the manufacturer’s instructions. Quantitative PCR (qPCR) using SYBR Green Technology was carried out on an Applied Biosystems 7500 real-time PCR system. Data were analyzed using the 2-ΔΔCt method (*99*). A list of qPCR primer sequences is provided in table S8.

### Protein extraction and Western blotting

INS-1 832/3 *Glp1r*^−/−^ SNAP/FLAG-*hGLP1R* ^WT^ or ^A316T^ cells were treated with vehicle or exendin-4 for 5 min, washed once in cold PBS, and lysed in 1× TNE lysis buffer (20 mM tris, 150 mM NaCl, 1 mM EDTA, 1% NP-40, and protease and phosphatase inhibitors) for 10 min at 4°C. Samples were then sonicated in a water bath sonicator 3× 10 s and centrifuged at 13,000 rpm for 5 min. Supernatants were collected and resolved by SDS–polyacrylamide gel electrophoresis (10% acrylamide gels) in 2× urea loading buffer [200 mM tris-HCl, 5% (w/v) SDS, 8 M urea,100 mM DTT, and 0.02% (w/v) bromophenol blue (pH 6.8)]. A Bio-Rad wet transfer system was used for immunoblotting onto polyvinylidene difluoride membranes (Immobilon-P, 0.45-μm pore size, IPVH00010, Merck) before incubation with appropriate primary and secondary antibodies in 5% skimmed milk. SNAP-tagged GLP-1R was detected with an anti-SNAP tag rabbit polyclonal antibody (1:500; P9310S, New England Biolabs) followed by goat anti-rabbit immunoglobulin G horseradish peroxidase (HRP; 1:2000; Abcam). Poststripping, tubulin was labeled with anti–α-tubulin mouse monoclonal antibody (1:5000; T5168, Sigma-Aldrich) followed by sheep anti-mouse secondary antibody HRP (1:5000; Abcam). Blots were developed using Clarity Western ECL Substrate System (Bio-Rad) in a Xograph Compact X5 processor, and specific band densities were quantified in Fiji.

### Cryo-EM determination of GLP-1:GLP-1R (A316T):Gα_s_ complex structure

#### Construct design

A human GLP-1R construct with residue Ala^316^ substituted for Thr (c.946G>A) was generated from the WT sequence by Q5 enzyme site-directed mutagenesis (New England Biolabs) and cloned into the pFastBac vector. The native signal peptide was replaced with hemagglutinin to enhance expression, and an N-terminal FLAG epitope and a C-terminal 6xHis epitope were added as affinity tags with 3C protease cleavage sites. These modifications do not significantly alter the function of WT GLP-1R (*100*).

#### Purification of GLP-1:GLP-1R (A316T):DN-Gα_s_:Nb35

A-FLAG-GLP-1R (A316T)-His, DN-Gα_S_, and Gβ_1_γ_2_-His P2 baculoviruses were coinfected in a 500-ml culture of *Trichoplusia ni* Hi5 insect cells in suspension (density of 3 to 4 × 10^6^ cells/ml), at a ratio of 2:1:1. After 48 hours, the cell preparations were harvested by centrifugation at 8000g for 10 min, and the supernatant was discarded. The pelleted cell mass was stored at −80°C until use. For purification, cell preparations were rapidly thawed and resuspended in 50 ml of buffer. Receptor–G protein complex was formed in the presence of 10 μM GLP-1 for 30 min at room temperature with gentle stirring. After this period, nanobody 35 (Nb35, 1 mg) and apyrase (5 μl/liter of starting culture, equivalent to 2.5 U) were added to the resuspension and incubated at room temperature for a further 30 min. Purified Nb35 was a gift from M. Baruah.

#### Vitrification and data collection of purified samples

Two microliters of sample was applied to glow-discharged R1.1/1.2 gold foil grids, manufactured by Quantifoil at the Melbourne Centre for Nanofabrication immediately followed by plunge-freezing in liquid ethane using a Vitrobot Mark IV (Thermo Fisher Scientific) set at 4°C, 100% humidity. At least three grids were prepared for each sample, and blot force and blot time varied between 16 to 18 and 6 to 8 s, respectively. Vitrification conditions for grids used in data collection were as follows: blot force 16 and blot time 7 s. Cryo-EM data were collected on a Glacios cryo–transmission electron microscopy (cryo-TEM; Thermo Fisher Scientific) operating at 200-kV accelerating voltage in cryo-TEM nanoprobe mode with a 100-μm objective aperture inserted. Data were collected using aberration-free image shift in fast acquisition mode in the EPU software (Thermo Fisher Scientific).

#### Data processing for GLP-1:GLP-1R (A316T):DN-Gα_s_:Nb35

Three thousand one hundred seventy micrographs were assigned to 69 optics groups based on their beam-image shift coordinates, motion corrected in RELION 3.1.2, and contrast transfer function (CTF) parameters were estimated using CTFFIND 4.1 within RELION (*101*). For quality control, micrographs with an estimated maximum resolution of >20 Å were excluded, leaving 2940 micrographs in the dataset. A total of 1,667,111 particles were picked using a pretrained general model in crYOLO 1.7.6 (*102*) and extracted with 4.5-fold binning. These particles were imported into cryoSPARC 3.2 (*103*) and subjected to rounds of 2D classification to homogenize the particle stack, with 583,410 particles remaining. Particles were reextracted at the full box size (288 px2) and subjected to Bayesian polishing in RELION 3.1.2. The polished particles were reimported into cryoSPARC, filtered for polishing artifacts by 2D classification, and a final set of 330,000 was subjected to nonuniform refinement to obtain a 3.3-Å global resolution consensus density map. Atomic model refinement used an existing model of WT GLP-1R bound to GLP-1 and DN-Gα_s_ proteins (PDB: 6X18) where Ala316 of GLP-1R was mutated to Thr. The mutated model was fitted into the experimental density map and subjected to molecular dynamics flexible fitting (MDFF) refinement using Isolde (*104*) within ChimeraX 1.5 (*105*). Iterative rounds of real-space refinement in PHENIX 1.20 (*106*), followed by MDFF in Isolde, were used to obtain the final model for analysis. Data collection, processing, and refinement statistics for the cryo-EM of GLP-1:GLP-1R (A316T):DN-Gα_s_:Nb35 complex are summarized in table S9.

### Computational methods and MD simulations

#### MD simulations of the A316T and WT GLP-1R structures

The cryo-EM GLP-1:GLP-1R (A316T):Gα_s_ complex structure and the corresponding GLP-1:GLP-1R(WT):Gα_s_ [PDB 6X18 (*64*)] were subjected to the same preparation for MD simulations. The missing stalk region (residues 130 to 135) and ICL3 (residues 339 to 343) of 6X18 were added by superimposition from the GLP-1:GLP-1R:Gα_s_ structure simulated in (*9*), and Gα_s_ was removed. The resulting systems (i.e., WT and A316T) were superimposed with the PDB 6X18 OPM reference (*107*) to opportunely orient the receptor and parameterized with the CHARMM36 force field (*108*), using in-house Python HTMD (*109*) and Tool Command Language scripts. Hydrogen atoms were added at a simulated pH of 7.0 using the pdb2pqr (*110*) and propka (*111*) software, and the protonation of titratable side chains was checked by visual inspection to correctly orient the receptor before inserting (*112*) in a rectangular 100 Å–by–101 Å 1-palmitoyl-2-oleyl-sn-glycerol-3-phosphocholine (POPC) bilayer (previously built by using the VMD Membrane Builder plugin 1.1 at http://www.ks.uiuc.edu/Research/vmd/plugins/membrane/), removing the lipid molecules overlapping the receptor TMD bundle. TIP3P water molecules (*113*) were added to the simulation box (100 Å by 101 Å by 136 Å) using the VMD Solvate plugin 1.5 at http://www.ks.uiuc.edu/Research/vmd/plugins/solvate/. Overall charge neutrality was lastly reached by adding Na^+^/Cl^−^ counter ions (final ionic concentration of 0.150 M), using the VMD Autoionize plugin 1.3 at http://www.ks.uiuc.edu/Research/vmd/plugins/autoionize/. For WT and A316T GLP-1Rs, equilibration and production replicas were computed using ACEMD (*114*). For the equilibration, isothermal-isobaric conditions [Berendsen barostat (*115*) with a target pressure of 1 atm; Langevin thermostat (*116*) with a target temperature of 300 K and damping of 1 ps^−1^] were used to equilibrate the systems through a multistage procedure (integration time step of 2 fs). First, clashes between lipid atoms were reduced through 1500 conjugate-gradient minimization steps. A positional constraint of 1 kcal/mol Å^−2^ lipid phosphorus atoms, protein Ca carbon, and all the other protein-heavy atoms was applied and gradually released over 4, 100 and 80 ns, respectively, before further 50 ns without any restraints for a total of 150 ns. Productive trajectories (2.5 Å ms for each replica, 7.5 ms for each system) were computed with an integration time step of 4 fs in the canonical ensemble (NVT) at 300 K, using a thermostat damping of 0.1 ps^−1^ and the M-SHAKE algorithm (*117*) to constrain the bond lengths involving hydrogen atoms. The cutoff distance for electrostatic interactions was set at 9 Å, with a switching function applied beyond 7.5 Å. Long-range Coulomb interactions were handled using the particle mesh Ewald summation method (*118*) by setting the mesh spacing to 1.0 Å.

#### GLP-1R WT and A316T TMD simulations

Using the GLP-1:GLP-1R:Gα_s_ cryo-EM complex [PDB 6X18 (*64*)] as the starting structure, we removed Gα_s_, GLP-1, ECD, and ECL1 to minimize the possible interference with the GLP-1R TMD dynamics (fig. S10A). Position 316 of the resulting TMD WT was mutated from Ala to Thr to model the A316T TMD and after membrane insertion and solvation, the two systems (i.e., TMD WT and TMD A316T) underwent 12 ms of MD each; ICL3 residues 339I to 343 were modeled using Modeller 9.18 (*119*) through Chimera (*105*) (v1.14) user interface, considering the best DOPE score. A316T was modeled using Chimera. The resulting GLP-1R TMD systems (i.e., WT and A316T) were prepared for MD simulations as reported above, after superimposition with the PDB 6X18 OPM reference (*107*), with the CHARMM36 force field (*108*) by inserting (*112*) them in a rectangular 96 Å–by–96 Å POPC bilayer. For both WT and A316T, three independent equilibrations and production replicas were computed using ACEMD (*114*), and the settings reported above productive trajectories (4 ms for each replica, 12 ms for each system) were computed with an integration time step of 4 fs in the canonical ensemble (NVT) at 300 K.

#### MD simulations of exendin-F1 (Ex-F1) in complex with A316T and WT GLP-1R

The Ex-F1:GLP-1R (A316T):Gα_s_ was modeled from the Ex-F1:GLP-1R(WT):Gα_s_ complex structure (PDB 9C0K) by mutating in silico A316^5.46^ to T316^5.46^. Ex-F1:GLP-1R(WT):Gα_s_ and Ex-F1:GLP-1R (A316T):Gα_s_ were prepared for MD simulations by superimposing the structures on GLP-1R coordinates retrieved from the OPM database to opportunely orient the receptor before being inserted in a rectangular 125 Å–by–116 Å POPC bilayer, removing the lipid molecules overlapping the receptor TMD bundle. TIP3P water molecules (*113*) were added to the simulation box (125 Å by 116 Å by 190 Å), and overall charge neutrality was lastly reached by adding Na^+^/Cl^−^ counter ions (final ionic concentration of 0.150 M). Equilibration and MD productive simulations computed using ACEMD in isothermal-isobaric conditions (target pressure 1 atm; target temperature 310 K) were used to equilibrate the systems through 125 ns of simulation (integration time step of 2 fs). First, clashes between lipid atoms were reduced through 2500 conjugate-gradient minimization steps, and then a positional constraint of 1 kcal mol^−1^ Å^−2^ on protein and lipid phosphorus atoms was applied. Lipids restraints were gradually released over a time window of 6 ns, protein atoms other than alpha carbon atoms were gradually released from the restraints over 100 ns, while α carbon atoms were gradually released over 120 ns. No restraint was applied in the last 5 ns of equilibration. Productive trajectories (four 500 nanosecond-long replicas for each GLP-1R complex) were computed with an integration time step of 4 fs in the canonical ensemble (NVT) at 310 K as reported above.

#### MD analysis

Atom distances were computed at the Cα level using MDTraj (*120*). H-bonds were quantified using the GetContacts analysis tool (https://getcontacts.github.io/) as the percentage of frames over all the frames obtained by merging the different replicas in which they were present. Water molecule occupancy was computed using the VMD (*121*) Volmap plugin (https://www.ks.uiuc.edu/Research/vmd/plugins/volmapgui/) with 1-Å resolution.

#### Numbering system

Throughout the manuscript, the Wootten residues numbering system for class B GPCRs (*122*) was adopted.

### Statistical analyses

All data analyses and graph generation were performed with GraphPad Prism 10.1.2. The *n* numbers refer to the number of independent biological replicates, representing the number of mice per genotype for in vivo experiments or the number of biologically independent experiments performed from islets isolated from separate mice or from a separate batch of cells, for ex vivo or in vitro experiments, respectively. Technical replicates within the same assay were averaged to determine the mean value for each biological replicate, before statistical tests as indicated in the corresponding figure legends. Statistical significance was assessed with the indicated test, with matched analyses performed for matched design experiments. Statistical significance was inferred when *P* < 0.05. Unless indicated, values represented are mean ± SEM.

## Supplementary Materials

### The PDF file includes:

Supplementary Methods Figs. S1 to S10 Tables S1 to S9 Legend for movie S1 References
