# Supplementary material for: In vivo functional profiling and structural characterization of the human GLP1R A316T variant
Source: Sci Adv. 2026 Feb 4;12(6):eadw0899. doi: 10.1126/sciadv.adw0899 (PMC13267291; doi:10.1126/sciadv.adw0899)
Supplement: Supplementary file 1 — Supplementary Methods Figs. S1 to S10 Tables S1 to S9 Legend for movie S1 References [file sciadv.adw0899_sm.pdf]

Science Advances
AAAS

Supplementary Materials for

In vivo functional profiling and structural characterization of the human *GLP1R* A316T variant

Liliane El Eid *et al.*

Corresponding author: Alejandra Tomas, a.tomas-catala@imperial.ac.uk

Sci. Adv. **12**, eadw0899 (2026)
DOI: 10.1126/sciadv.adw0899

The PDF file includes:

Supplementary Methods
Figs. S1 to S10
Tables S1 to S9
Legend for movie S1
References

Other Supplementary Material for this manuscript includes the following:

Movie S1

Supplementary Methods

Nb37 bystander *NanoBiT assays*

INS-1 832/3 *Glp1r*$^{-/-}$ cells were seeded in 6-well plates and co-transfected with the following plasmids: 200 ng pSNAP/FLAG-*hGLP1R*^{WT} or ^{A316T}, 500 ng Gα_s (human, short isoform), 500 ng Gβ1 (human), 500 ng Gγ2 (human), 100 ng RIC8B (human, isoform 2), and either 100 ng LgBiT-CAAX (KRAS CAAX motif N-terminally fused with LgBiT) or 500 ng Endofin-LgBiT (Endofin FYVE domain C-terminally fused with LgBiT), plus 100 ng (plasma membrane assay) or 500 ng (endosomal assay) SmBiT-Nb37 [Nb37 cDNA synthesised by GenScript C-terminally fused to SmBiT with a 15 amino acid flexible linker (*123*)] and adjusted for total DNA with pcDNA3.1 for the LgBiT-CAAX transfection. 24 hours after transfection, cells were exposed to NanoGlo Live Cell Reagent (Promega) with furimazine (1:20 dilution) and bioluminescence read in a Flexstation 3 plate reader for 8 min before and 30 min after stimulation by serial doses of up to 1 µM GLP-1. AUCs were calculated for each agonist concentration and fitted to log(agonist) *versus* response four-parameter curves using GraphPad Prism 10.1.2, with $logEC_{50}$ (potency) and E_{max} (efficacy) calculated for each replicate dose response curve. Baseline endosomal over plasma membrane bioluminescence ratios were also calculated.

GLP-1R conformational ECD opening time resolved-fluorescence resonance energy transfer (TR-FRET) assay

INS-1 832/3 *Glp1r*$^{-/-}$ SNAP/FLAG-*hGLP1R*^{WT} or ^{A316T} cells were labelled with SNAP-Lumi4-Tb (Revvity; 40 nM, 60 min at 37°C in complete medium). After washing to remove unbound probe, cells were resuspended in HBSS ± 100 nM plasma membrane dye NR12A (a gift from Prof Andrey Klymchenko, University of Strasbourg, France) for 5 min before washing again. Labelled cells were transferred to 96-well half-area white plates and baseline TR-FRET signal from Lumi-4Tb (donor) and NR12A (acceptor) recorded for 5 min at 37°C using a Flexstation 3 plate reader with the following settings: λ_{ex} = 335 nm, λ_{em} = 490 and 590 nm, delay 50 µs, integration time 300 µs. GLP-1 was then added at serial doses, and

signal serially monitored for 30 min. The TR-FRET ratio, i.e. the ratio of fluorescence intensities at 590 and 490 nm, was considered indicative of the proximity of the GLP-1R ECD to the plasma membrane. Baseline-normalised AUCs were used to generate concentration–response curves fitted using GraphPad Prism 10.1.2, with $logEC_{50}$ and E_{max} calculated for each replicate dose response curve.

Real-time cytosolic calcium assay

hGLP1R$^{-/-}$ mouse islets were transduced with pAV-SNAP/FLAG-*hGLP1R*^{WT} or ^{A316T} adenoviruses for 24 hours, labelled with the Ca^{2+} dye Cal-520, AM (4.5 µM; Stratech) diluted in KBRH buffer containing 6 mM glucose for 1 hour and encased in Matrigel onto 35-cm glass bottom MatTek dishes. Islets were then imaged every 6 s at 488 nm using a Nikon Eclipse Ti microscope with an ORCA-Flash 4.0 camera (Hamamatsu) and Metamorph software (Molecular Devices) at 37°C on a heated stage before and after addition of the indicated GLP-1RA followed by 11 mM glucose and then 20 mM KCl at the indicated time points. Raw fluorescence intensity traces from islet ROIs were extracted using Fiji. Responses were plotted relative to the average fluorescence intensity during the 6 mM glucose baseline period, before agonist addition, and AUCs separately calculated for the GLP-1RA + 6 mM and 11 mM glucose stimulation periods.

Isolation of detergent-resistant membrane (DRM) fractions

To purify lipid rafts, DMR fractions were isolated from INS-1 832/3 *Glp1r*$^{-/-}$ SNAP/FLAG-*hGLP1R*^{WT} or ^{A316T} cells by ultracentrifugation based on their insolubility in non-ionic detergents at 4°C and their low density because of their high lipid-to-protein ratio. Cells seeded onto 6-cm dishes were stimulated ± 100 nM exendin-4 or GLP-1 for 2 min, washed with ice-cold PBS and osmotically lysed in cold low salt 20 mM tris-HCl, pH 7.0 buffer supplemented with cOmplete EDTA-free protease inhibitor cocktail (Roche) and phosphatase inhibitor cocktail 2 (Sigma-Aldrich) for 10-15 minutes on a rocking platform at 4°C. The cell suspension was collected and homogenised by passing through a 21-gauge needle, followed by ultracentrifugation at 63,000 g for 1 hour at 4°C with a Sorvall Discovery M120 ultracentrifuge. Pellets

were resuspended in 200 µL ice-cold PBS supplemented with cOmplete EDTA-free protease inhibitor cocktail (Roche) and phosphatase inhibitor cocktail 2 and 1% Triton X-100, followed by rotating incubation for 30 min at 4°C. Detergent-treated membrane fractions were re-centrifuged at 63,000 g for 1 hour at 4°C. The supernatants, containing the 'disordered' detergent-soluble membrane (DSM) fractions, were retained for analysis, while the 'ordered' DRM fraction pellets were resuspended in 1% SDS supplemented with protease inhibitor and phosphatase inhibitor cocktails as above, sonicated with the Ultrasonic cell disruptor XL-2000 probe sonicator (Misonix, Inc) and centrifuged at 14,000 g for 5 min at 4 °C before Western blot analysis with anti-SNAP rabbit polyclonal antibody (1:500; P9310S, New England Biolabs) and goat anti-rabbit HRP secondary (1:2,000; ab6721, Abcam), stripping and reblotting with anti-flotillin-1 mouse monoclonal antibody (1:200; sc-74566, Santa Cruz Biotechnology) and sheep anti-mouse HRP secondary (1:5,000; ab6808, Abcam) as a DRM loading control. GLP-1R enrichment into DRMs was calculated from SNAP over flotillin band intensity levels under the different conditions tested using Fiji.

Raster Image Correlation Spectroscopy (RICS) image capture and analysis

INS-1 832/3 *Glp1r*$^{-/-}$ SNAP/FLAG-*hGLP1R*^{WT} or ^{A316T} cells were seeded onto 35-cm glass bottom MatTek dishes and labelled with SNAP-Surface Alexa Fluor 647 for 10 min at 37°C in full media, washed and imaged in RMPI-1640 media without phenol red using a 100x oil objective on a Leica Stellaris 8 STED FALCON microscope from the Imperial College FILM Facility, using confocal settings. Cells were imaged and analysed as previously described (*60*), at the basal plasma membrane, both under vehicle conditions and following stimulation with 100 nM exendin-4 with a format size of 256 x 256 pixels and 80 nm pixel size for 200 consecutive frames. RICS analysis was carried out using the SimFCS 4 Software (Global Software, G-SOFT Inc) to determine the diffusion coefficient of WT versus A316T GLP-1R in vehicle and exendin-4-stimulated conditions.

GLP-1R ubiquitination assays

INS-1 832/3 *Glp1r*$^{-/-}$ SNAP/FLAG-*hGLP1R*^{WT} or ^{A316T} cells plated onto 6-cm dishes were stimulated ± 100 nM exendin-4 for 10 min, washed with PBS and osmotically lysed in lysis buffer (50 mM tris-HCl, pH 7.4, 150 mM NaCl, 1% Triton X-100, 1 mM EDTA) with 1% protease inhibitor (Roche) and 1% phosphatase inhibitor cocktail (Sigma-Aldrich) at 4°C. Cell lysates were centrifuged at 12,000 g and 4°C for 10 min prior to the addition of 40 µL anti-FLAG M2 beads (A2220, Sigma-Aldrich) and overnight rotating incubation at 4°C to increase FLAG-tag binding efficiency. Samples were then centrifuged at 5,000 g for 30 s, bead pellets washed 3x in 500 µL wash buffer (50 mM Tris-HCl, 150 mM NaCl, pH 7.4), and proteins eluted with 2x urea buffer at 37°C for 10 min before Western blot analysis with anti-ubiquitin antibody (1:1,000; sc-8017, Santa Cruz Biotechnology) and sheep anti-mouse HRP secondary (1:5,000; ab6808, Abcam) followed by stripping and reblotting with anti-SNAP antibody as above to assess immunoprecipitated receptor per sample. GLP-1R ubiquitination was calculated from ubiquitin over SNAP band intensity levels under the different conditions tested using Fiji.

LC-MS/MS GLP-1R interactome analysis

INS-1 832/3 *Glp1r*$^{-/-}$ SNAP/FLAG-*hGLP1R*^{WT} or ^{A316T} cells were plated onto 6-cm dishes and stimulated with vehicle or 100 nM GLP-1RA for 5 min as previously described (*63*). Following stimulation, GLP-1R was immunoprecipitated with anti-FLAG M2 beads as above and bead pellets washed 5x in wash buffer (50 mM tris-HCl, 150 mM NaCl, pH 7.4), before storage at -80°C until further analysis. Beads were resuspended in 150 µL urea 1 M, 20 mM HEPES, 1 mM DTT, pH 8.0 followed by addition of 1.5 µg trypsin gold (Promega) and overnight digestion at 37°C. Supernatants were subsequently acidified with 0.5% trifluoroacetic acid (TFA), followed by desalting using reversed-phase spin tips (Glygen Corp) and dried using vacuum centrifugation. Dried peptides were resuspended in 0.1% TFA and Liquid Chromatography Tandem Mass Spectrometry (LC-MS/MS) performed as previously described (*124*) using an Ultimate 3000 nano-HPLC coupled to a Q-Exactive mass spectrometer (Thermo Scientific) via an EASY-Spray source. Peptides were loaded onto a trap column (Acclaim PepMap 100 C18, 100 µm x 2 cm) at 8 µL/min in 2% acetonitrile, 0.1% TFA. Peptides were eluted on-line to an analytical column

(Acclaim Pepmap RSLC C18, 75 µm x 75 cm). A 90-min stepped gradient separation was used with 4-25% acetonitrile 80%, formic acid 0.1% for 60 min, followed by 25-45% for 30 min. Eluted peptides were analysed by Q-Exactive operating in positive polarity and data-dependent acquisition mode. Ions for fragmentation were determined from an initial MS1 survey scan at 70,000 resolution (at m/z 200), followed by higher-energy collisional dissociation of the top 12 most abundant ions at a resolution of 17,500. MS1 and MS2 scan AGC targets were set to 3e6 and 5e4 for a maximum injection time of 50 and 75 ms, respectively. A survey scan m/z range of 350 – 1800 m/z was used, with a normalised collision energy set to 27%, minimum AGC of 1e3, charge state exclusion enabled for unassigned and +1 ions and a dynamic exclusion of 45 seconds. Data was processed using the MaxQuant software platform (v1.6.10.43) (*125*), with database searches carried out by the in-built Andromeda search engine against the Swissprot Rattus Norvegicus database (downloaded – 21st May 2022, entries: 8,132) concatenated with the human GLP-1R protein sequence. A reverse decoy database approach was used at a 1% FDR for peptide spectrum matches and protein identifications. Search parameters included: maximum missed cleavages set to 2, variable modifications of methionine oxidation, protein N-terminal acetylation, asparagine deamidation and cyclisation of glutamine to pyroglutamate. Label-free quantification was enabled with a label-free quantitation (LFQ) minimum ratio count of 1. Proteins were filtered for those containing at least one unique peptide and identified in all biological replicates. For normalisation, proteins were ranked by fold change increase in abundance compared with control (vehicle-treated) immunoprecipitates. The resulting data was pre-processed by elimination of proteins that are non-specifically bound and residual background proteins, such as common contaminants and reverse database proteins. To further facilitate the selection of GLP-1R interactors, the following criteria were established; i) identified at least twice from four runs; ii) not nuclear proteins; and (iii) previously identified in pancreatic β-cells. Untargeted LFQ intensity values, normalised to hGLP-1R levels in each immunoprecipitated sample, were analysed by LFQ Analyst (*126*) to identify differences in levels of individual protein - GLP-1R interactions between vehicle and GLP-1RA treated conditions. Paired t-tests

adjusted for multiple comparisons using Benjamin-Hochberg false discovery rate correction and Perseus type imputations were used for statistical analysis.

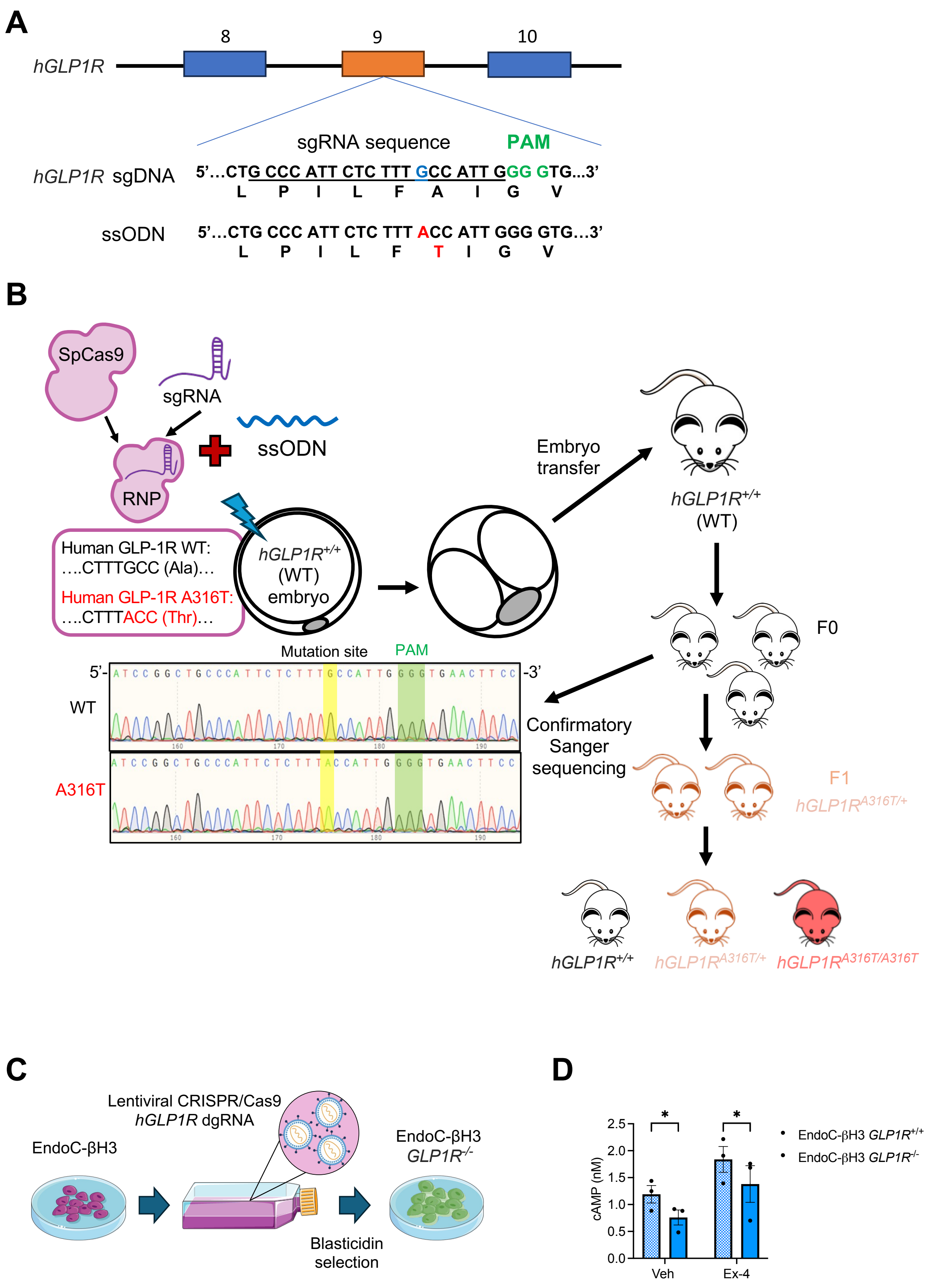

A
8
9
10
hGLP1R
sgRNA sequence
PAM
hGLP1R sgDNA
5'…CTG CCC ATT CTC TTT GCC ATT GGG GTG…3'
L P I L F A I G V
ssODN
5'…CTG CCC ATT CTC TTT ACC ATT GGG GTG…3'
L P I L F T I G V
B
SpCas9
sgRNA
ssODN
RNP
Human GLP-1R WT:
….CTTTGCC (Ala)…
Human GLP-1R A316T:
….CTTTACC (Thr)…
hGLP1R+/+ (WT) embryo
Embryo transfer
hGLP1R+/+ (WT)
F0
Mutation site
PAM
5'-
-3'
WT
A316T
Confirmatory Sanger sequencing
F1
hGLP1RA316T/+
hGLP1R+/+
hGLP1RA316T/+
hGLP1RA316T/A316T
C
Lentiviral CRISPR/Cas9
hGLP1R dgRNA
EndoC-βH3
EndoC-βH3 GLP1R-/-
Blasticidin selection
D
cAMP (nM)
2.5
2.0
1.5
1.0
0.5
0.0
Veh
Ex-4
*
*
EndoC-βH3 GLP1R+/+
EndoC-βH3 GLP1R-/-

Fig. S1. Model generation. (**A**) Targeting strategy for *hGLP1R*$^{A316T/A316T}$ knock-in mouse generation; CRISPR single guide RNA (sgRNA) targeting the Cas9 nuclease to the indicated PAM region of exon 9 in the human *GLP1R* gene and corresponding ssODN homology directed repair template sequences are indicated. (**B**) Schematic diagram of the method to generate *hGLP1R*$^{A316T/A316T}$ knock-in mice: electroporation of Cas9, sgRNA and ssODN templates into *hGLP1R*$^{+/+}$ pronucleus, followed by IVF transfer of two cell embryo into *hGLP1R*$^{+/+}$ female mice resulting in F0 generation and screening via genomic PCR and Sanger sequencing, with sequences from *hGLP1R*$^{+/+}$ and $^{A316T/A316T}$ mice included. (**C**) Schematic diagram of the method to generate EndoC-βH3 *GLP1R*$^{-/-}$ cells: transduction with engineered lentiviral CRISPR/Cas9 double guide RNA (dgRNA) vector targeting *hGLP1R* followed by blasticidin selection. (**D**) Static cAMP levels from EndoC-βH3 *GLP1R*$^{+/+}$ versus $^{-/-}$ cells under vehicle (Veh) and 30 min exendin-4 (Ex-4) stimulation.

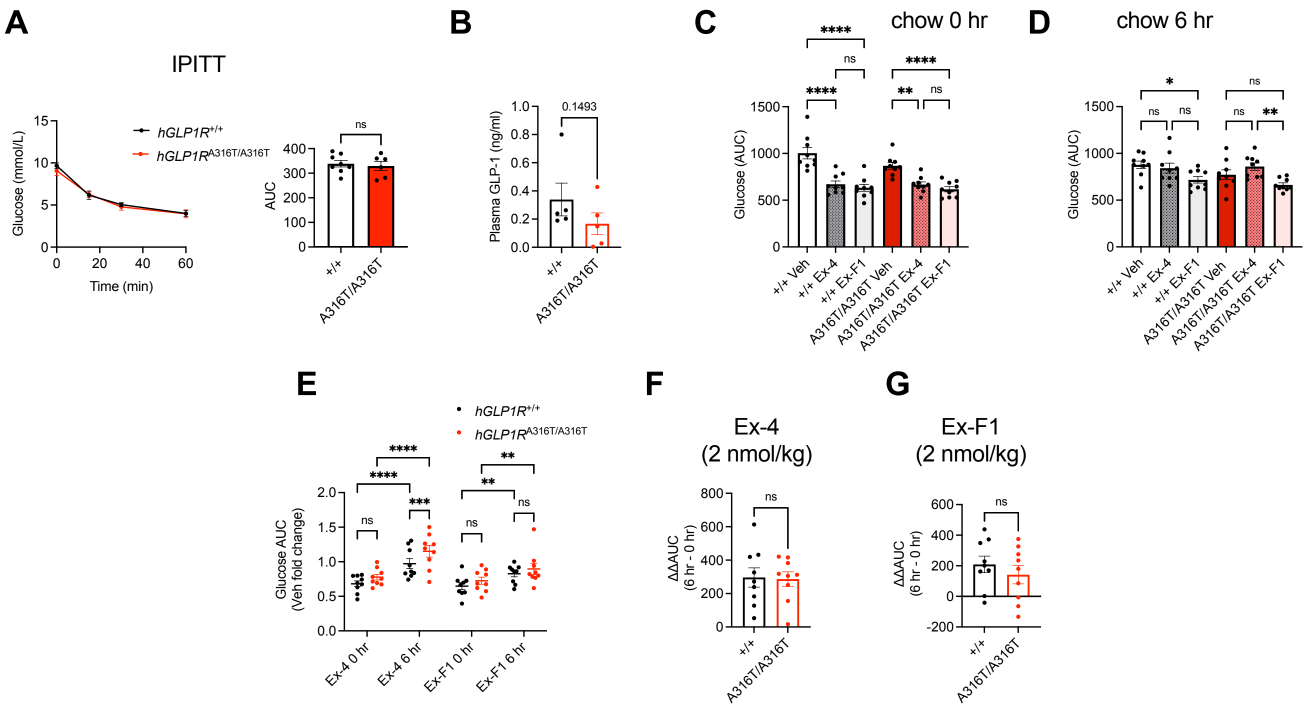

Fig. S2. In vivo glucose responses of adult *hGLP1R*$^{A316T/A316T}$ versus *hGLP1R*$^{+/+}$ mice on a chow diet – extra data. (**A**) IPITT responses and corresponding AUCs following 0.75 IU/kg body weight insulin ip administration after 5 hours of fasting in a mixed sex cohort of lean, adult *hGLP1R*$^{+/+}$ versus $^{A316T/A316T}$ mice on a chow diet; n = 8 for *hGLP1R*$^{+/+}$; n = 6 for *hGLP1R*$^{A316T/A316T}$. (**B**) Plasma GLP-1 levels 10 minutes post-oral administration of glucose (2 g/kg body weight) in chow-fed *hGLP1R*$^{+/+}$ versus $^{A316T/A316T}$ mixed sex adult mice; n = 8 per genotype. (**C**) AUCs from acute IPGTTs taken from **Fig. 1E**, **G** and **I**. (**D**) As in (**C**) for IPGTTs 6 hours post-vehicle or agonist administration. (**E**) Vehicle fold AUC responses calculated from **Fig. 1E**, **G** and **I**. (**F**) Exendin-4 (Ex-4) ΔΔAUC responses (6 hours minus acute) calculated from **Fig. 1J**. (**G**) Exendin-F1 (Ex-F1) ΔΔAUC responses (6 hours minus acute) calculated from **Fig. 1K**. Data are mean ± SEM; *p<0.05; **p<0.01; ***p< 0.001; ****p<0.0001; ns, not significant by paired or unpaired t-tests, or one- or two-way ANOVA with Sidak's post-hoc tests.

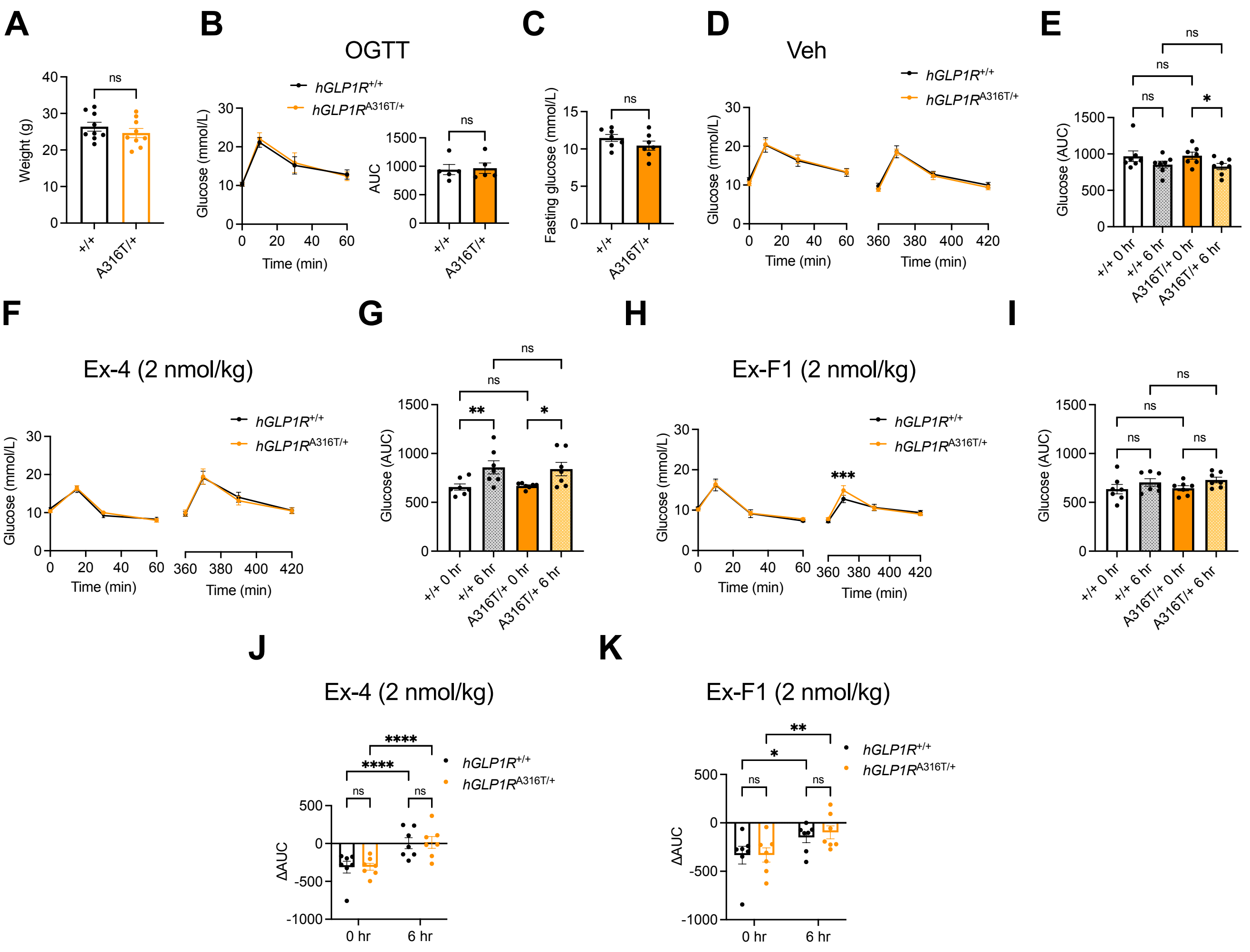

Fig. S3. In vivo glucose responses of adult *hGLP1R*$^{A316T/+}$ versus *hGLP1R*$^{+/+}$ mice on a chow diet. (**A**) Age-matched body weight of *hGLP1R*$^{+/+}$ versus $^{A316T/+}$ mixed sex littermate adult mice (n = 9 per genotype) on a chow diet. (**B**) OGTT responses and corresponding AUCs following 2 g/kg body weight glucose administration via oral gavage after 5 hours of fasting in a mixed sex cohort of lean, adult *hGLP1R*$^{+/+}$ versus $^{A316T/+}$ mice on a chow diet (n = 5 per genotype). (**C**) Glucose levels after 2 hours of fasting in chow fed *hGLP1R*$^{+/+}$ versus $^{A316T/+}$ mixed sex adult mice (n = 7 per genotype). (**D** and **E**) IPGTTs (2 g/kg body weight glucose) following acute (0 hours), or 6 hours post-ip administration of saline vehicle (Veh) in chow-fed *hGLP1R*$^{+/+}$ versus $^{A316T/+}$ mixed sex adult mice. Glucose curves (**D**) and corresponding AUCs (**E**) shown; n = 7 per genotype. (**F** to **I**) As in (**D** and **E**) following administration of 2 nmol/kg

exendin-4 (Ex-4) (**F** and **G**) or exendin-F1 (Ex-F1) (**H** and **I**). (**J** and **K**) Vehicle-corrected ΔAUC responses from (**G**) and (**I**), respectively. Data are mean ± SEM; *p<0.05; **p<0.01; ***p< 0.001; ****p<0.0001; ns, not significant by paired t-tests, one- or two-way ANOVA with Sidak's post-hoc tests.

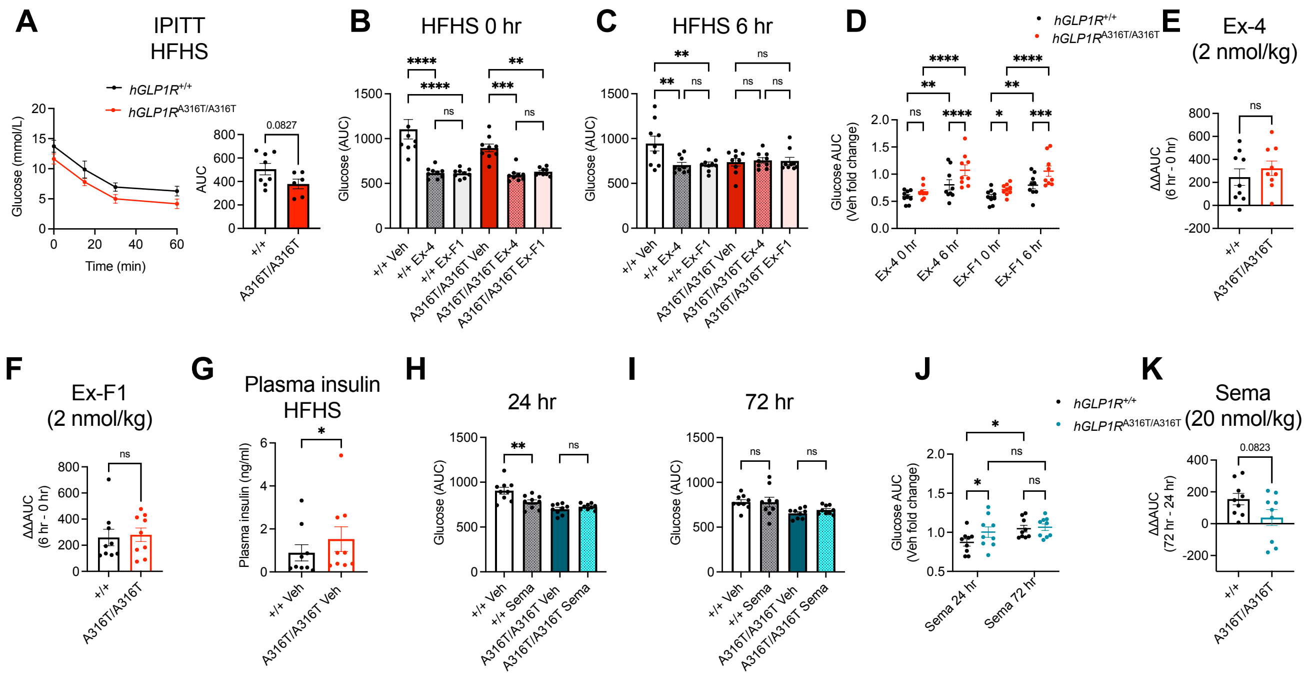

Fig. S4. In vivo glucose responses of adult *hGLP1R*$^{A316T/A316T}$ versus *hGLP1R*$^{+/+}$ mice on a HFHS diet – extra data. (**A**) IPITT responses and corresponding AUCs following 0.75 IU/kg body weight insulin ip administration after 5 hours of fasting in a mixed sex cohort of HFHS-fed adult *hGLP1R*$^{+/+}$ versus $^{A316T/A316T}$ mice; *n* = 8 for *hGLP1R*$^{+/+}$; *n* = 6 for *hGLP1R*$^{A316T/A316T}$. (**B**) AUCs from acute IPGTTs taken from **Fig. 2F**, **H** and **J**. (**C**) As in (**B**) for IPGTTs at 6 hours post-vehicle or agonist administration. (**D**) Vehicle fold AUC responses calculated from **Fig. 2F**, **H** and **J**. (**E**) Exendin-4 (Ex-4) ΔΔAUC responses (6 hours minus acute) calculated from **Fig. 2K**. (**F**) Exendin-F1 (Ex-F1) ΔΔAUC responses (6 hours minus acute) calculated from **Fig. 2L**. (**G**) Acute plasma insulin levels post-vehicle (Veh) administration in HFHS-fed *hGLP1R*$^{+/+}$ versus $^{A316T/A316T}$ mixed sex adult mice. (**H**) AUCs from IPGTTs at 24 hours post-vehicle (Veh) or agonist administration taken from **Fig. 2N** and **P**. (**I**) As in (**H**) for IPGTTs at 72 hours post-vehicle (Veh) or agonist administration. (**J**) Vehicle (Veh) fold AUC responses calculated from **Fig. 2N** and **P**. (**K**) Semaglutide (Sema) ΔΔAUC responses (72 hours minus 24 hours) calculated from **Fig. 2Q**. Data are mean ± SEM; *p<0.05; **p<0.01; ***p< 0.001; ****p<0.0001; ns, not significant by paired, unpaired or ratio paired t-tests, or one- or two-way ANOVA with Sidak's post-hoc tests.

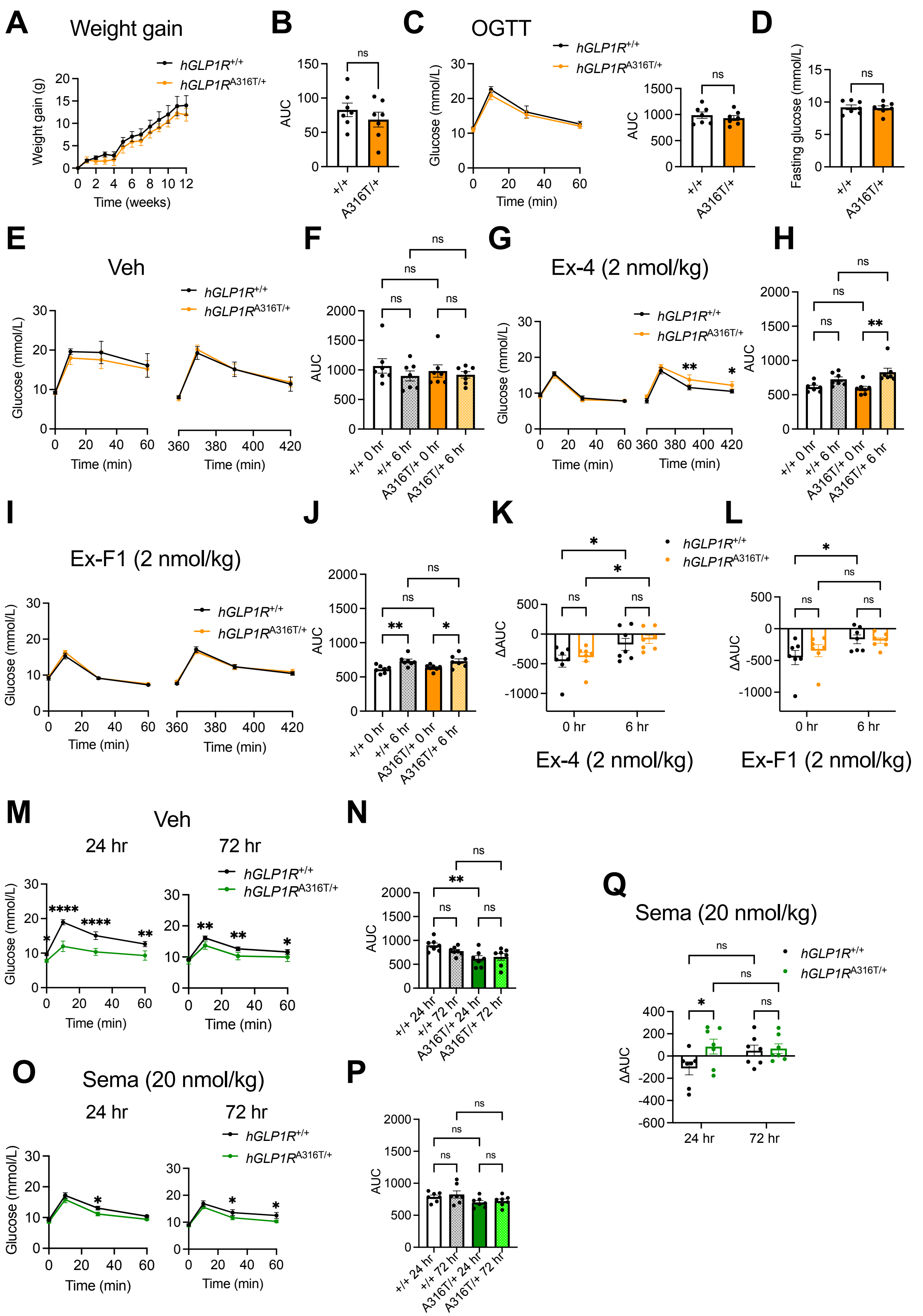

A
Weight gain
hGLP1R+/+
hGLP1RA316T/+
Weight gain (g)
Time (weeks)
B
AUC
ns
+/+
A316T/+
C
OGTT
Glucose (mmol/L)
Time (min)
D
Fasting glucose (mmol/L)
E
Veh
F
+/+ 0 hr
+/+ 6 hr
A316T/+ 0 hr
A316T/+ 6 hr
G
Ex-4 (2 nmol/kg)
H
I
Ex-F1 (2 nmol/kg)
J
K
ΔAUC
0 hr
6 hr
Ex-4 (2 nmol/kg)
L
Ex-F1 (2 nmol/kg)
M
Veh
24 hr
72 hr
N
+/+ 24 hr
+/+ 72 hr
A316T/+ 24 hr
A316T/+ 72 hr
O
Sema (20 nmol/kg)
P
Q
Sema (20 nmol/kg)

Fig. S5. In vivo glucose responses of adult *hGLP1R*$^{A316T/+}$ versus *hGLP1R*$^{+/+}$ mice on a HFHS diet. (**A**) Body weight gain for *hGLP1R*$^{+/+}$ versus $^{A316T/+}$ mixed sex littermate adult mice (*n* = 7 per genotype) maintained on a HFHS diet for the indicated times. (**B**) AUC from (**A**). (**C**) OGTT responses and corresponding AUC following 2 g/kg body weight glucose administration via oral gavage after 5 hours of fasting in a mixed sex cohort of adult *hGLP1R*$^{+/+}$ versus $^{A316T/+}$ mice on a HFHS diet (*n* = 7 per genotype). (**D**) Glucose levels after 2 hours of fasting from mice in (**A**). (**E** and **F**) IPGTTs (2 g/kg body weight glucose) following acute (0 hours), or 6 hours post-ip administration of saline vehicle (Veh) in HFHS-fed *hGLP1R*$^{+/+}$ versus $^{A316T/+}$ mixed sex adult mice. Glucose curves (**E**) and corresponding AUCs (**F**) shown; *n* = 7 mice per genotype. (**G** to **J**) As in (**E** and **F**) following administration of 2 nmol/kg exendin-4 (Ex-4) (**G** and **H**) or exendin-F1 (Ex-F1) (**I** and **J**). (**K** and **L**) Vehicle-corrected ΔAUC responses from (**H**) and (**J**), respectively. (**M** to **R**) Glucose curves and corresponding AUCs following vehicle (Veh) (**M** and **N**) or 20 nmol/kg semaglutide (Sema) (**O** and **P**) treatment of HFHS-fed *hGLP1R*$^{+/+}$ versus $^{A316T/+}$ mice; IPGTTs performed 24- and 72-hours post-agonist administration; *n* = 7 mice per genotype. (**Q**) Vehicle-corrected ΔAUC responses from (**P**). Data are mean ± SEM; *p<0.05; **p<0.01; ****p<0.0001; ns, not significant by paired t-tests, one- or two-way ANOVA with Sidak's post-hoc tests.

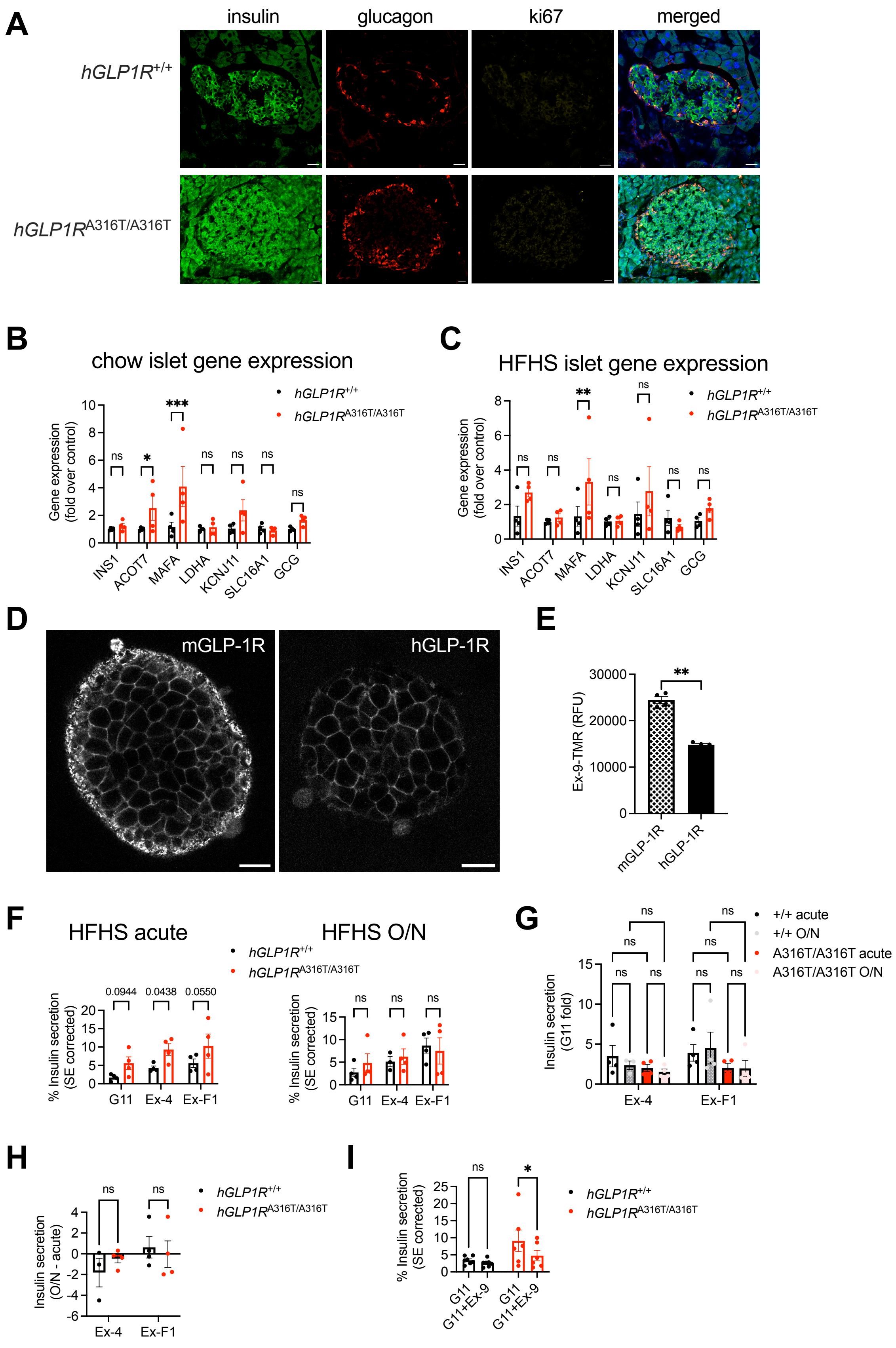
A
insulin
glucagon
ki67
merged
hGLP1R+/+
hGLP1RA316T/A316T
B
chow islet gene expression
Gene expression (fold over control)
hGLP1R+/+
hGLP1RA316T/A316T
INS1
ACOT7
MAFA
LDHA
KCNJ11
SLC16A1
GCG
C
HFHS islet gene expression
D
mGLP-1R
hGLP-1R
E
Ex-9-TMR (RFU)
mGLP-1R
hGLP-1R
F
HFHS acute
HFHS O/N
% Insulin secretion (SE corrected)
0.0944
0.0438
0.0550
G11
Ex-4
Ex-F1
G
Insulin secretion (G11 fold)
+/+ acute
+/+ O/N
A316T/A316T acute
A316T/A316T O/N
H
Insulin secretion (O/N - acute)
I
G11+Ex-9

Fig. S6. GLP-1R-induced downstream signalling in *hGLP1R*$^{A316T/A316T}$ versus *hGLP1R*$^{+/+}$ mouse islets – extra data. (**A**) Immunofluorescence analysis of insulin (green), glucagon (red) and ki67 (yellow) localisation in pancreas sections from HFHS-fed *hGLP1R*$^{+/+}$ versus $^{A316T/A316T}$ mice; nuclei (DAPI), blue; scale bars, 20 µm. (**B** and **C**) Relative gene expression of β-cell enriched and disallowed genes in islets purified from *hGLP1R*$^{A316T/A316T}$ versus *hGLP1R*$^{+/+}$ mice fed a chow (**B**) or HFHS (**C**) diet; *n* = 4. (**D**) Representative confocal microscopy images of islets from *hGLP1R* versus *mGlp1r* mice treated for 10 min with 100 nM exendin-9-TMR; scale bars, 20 µm. (**E**) Quantification of exendin-9-TMR (Ex-9-TMR) fluorescence intensity in islets from (**D**); *n* = 4. (**F**) Percentage of acute and overnight (O/N) surface expression (SE)-corrected insulin secretion in HFHS-fed *hGLP1R*$^{+/+}$ versus $^{A316T/A316T}$ mouse islets stimulated with 11 mM glucose (G11) alone or supplemented with 100 nM exendin-4 (Ex-4) or exendin-F1 (Ex-F1); *n* = 4. (**G**) Insulin secretion fold changes to G11 calculated from data in (**F**). (**H**) Overnight over acute insulin secretion responses calculated from data in (**G**). (**I**) Percentage of acute surface expression (SE)-corrected insulin secretion in HFHS-fed *hGLP1R*$^{+/+}$ versus $^{A316T/A316T}$ mouse islets stimulated with 11 mM glucose (G11) alone or supplemented with 100 nM exendin-9 (Ex-9); *n* = 7 for *hGLP1R*$^{+/+}$; *n* = 6 for *hGLP1R*$^{A316T/A316T}$. Data are mean ± SEM; *p<0.05; **p<0.01; ***p<0.001; ns, not significant by paired t-tests, or two-way ANOVA with Sidak's post-hoc tests.

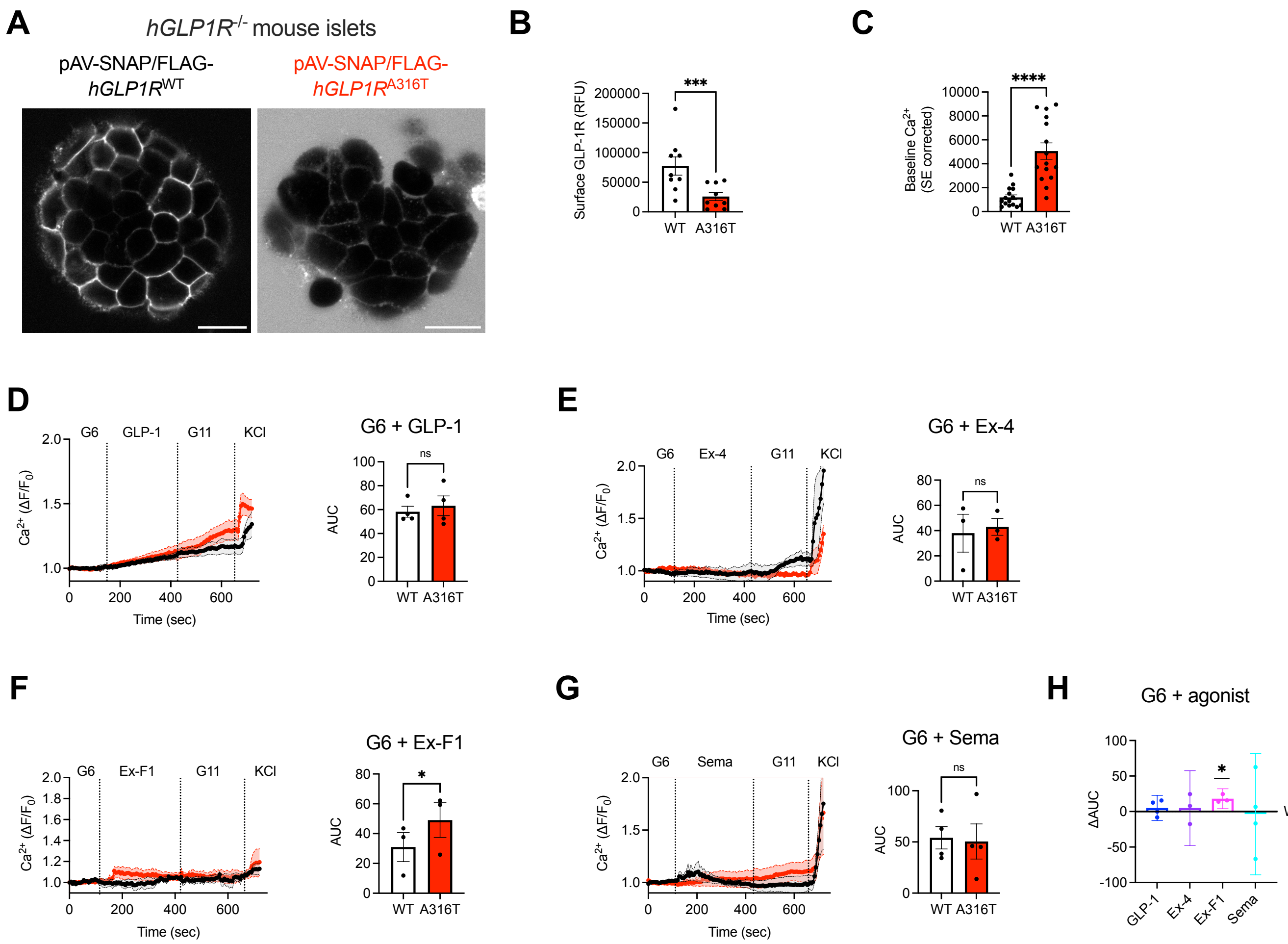

Fig. S7. GLP-1R surface expression and calcium signalling in *hGLP1R*$^{-/-}$ mouse islets transduced with SNAP/FLAG-*hGLP1R*^{WT} versus ^{A316T} adenoviruses. (**A** and **B**) Surface expression in islets from *hGLP1R*$^{-/-}$ mice transduced with pAV-SNAP/FLAG-*hGLP1R*^{WT} or ^{A316T} labelled with SNAP-Surface Alexa Fluor 647. Representative images (**A**) and quantification of surface GLP-1R (**B**); scale bars, 20 μm; *n* = 9. (**C**) Basal Ca^{2+} levels in Cal-520, AM-loaded *hGLP1R*$^{-/-}$ mouse islets transduced with pAV-SNAP/FLAG-*hGLP1R*^{WT} or^{A316T}; *n* = 15. (**D** to **G**) Ca^{2+} responses from islets from (**C**) stimulated with 100 nM GLP-1 (**D**), exendin-4 (Ex-4) (**E**), exendin-F1 (Ex-F1) (**F**), or semaglutide (Sema) (**G**), followed by 20 mM KCl for maximal responses; Ca^{2+} traces and corresponding AUCs for agonist responses at 6 mM glucose (G6) shown; *n* = 3-4. (**H**) ΔAUC (SNAP/FLAG-*hGLP1R*^{A316T} minus ^{WT}) responses calculated from data in (**D** to **G**). Data are mean ± SEM except for ΔAUC *hGLP1R*^{A316T} minus ^{WT} responses which

are mean ± 95% confidence interval (CI); *$p<0.05$; ***$p<0.001$; ns, not significant by paired or ratio paired t-tests.

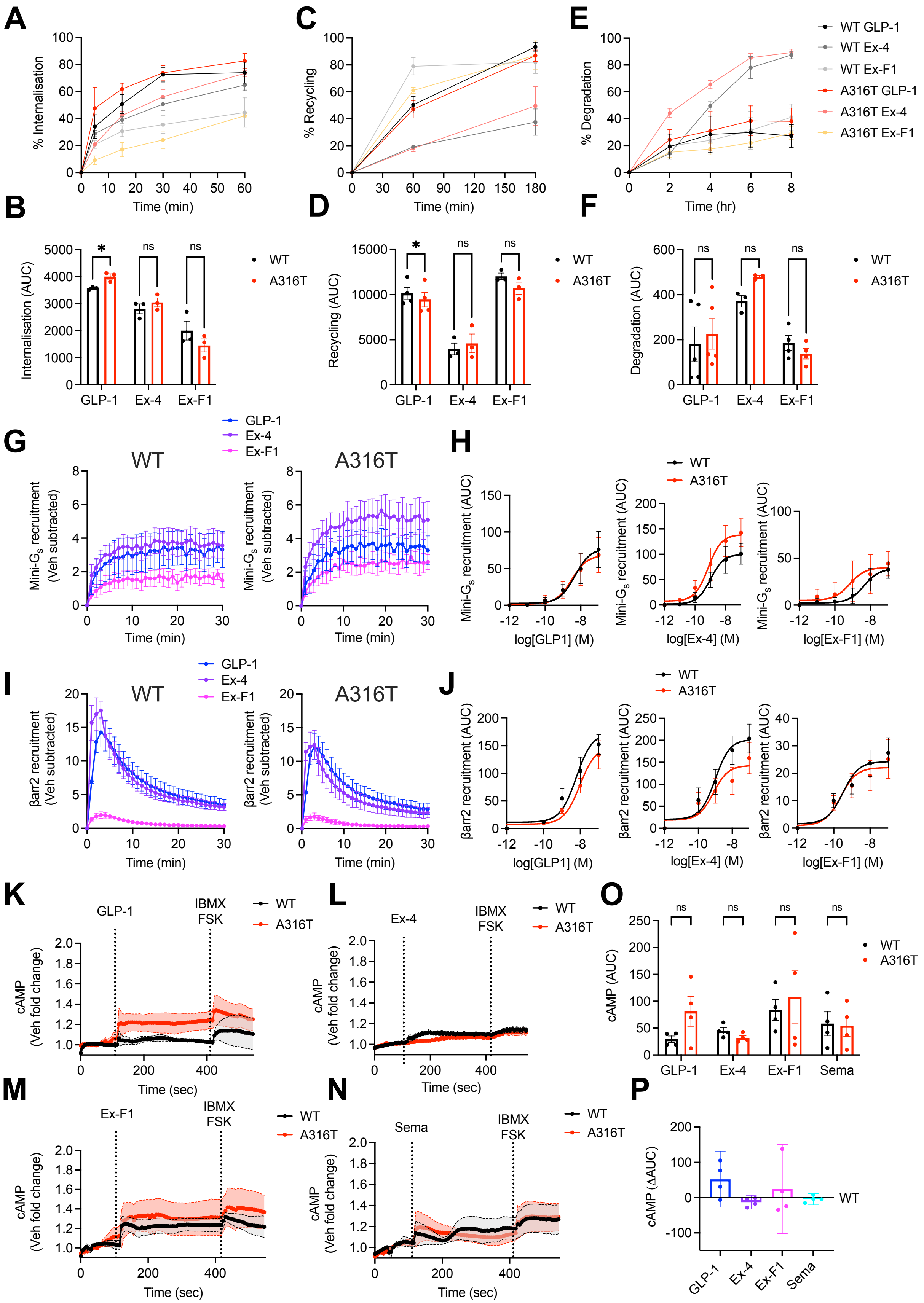
A
% Internalisation
Time (min)
C
% Recycling
Time (min)
E
% Degradation
Time (hr)
WT GLP-1
WT Ex-4
WT Ex-F1
A316T GLP-1
A316T Ex-4
A316T Ex-F1
B
Internalisation (AUC)
GLP-1
Ex-4
Ex-F1
WT
A316T
D
Recycling (AUC)
F
Degradation (AUC)
G
WT
A316T
Mini-Gs recruitment (Veh subtracted)
Time (min)
H
Mini-Gs recruitment (AUC)
log[GLP1] (M)
log[Ex-4] (M)
log[Ex-F1] (M)
I
βarr2 recruitment (Veh subtracted)
J
βarr2 recruitment (AUC)
K
GLP-1
IBMX FSK
cAMP (Veh fold change)
Time (sec)
L
Ex-4
O
cAMP (AUC)
Sema
M
Ex-F1
N
Sema
P
cAMP (ΔAUC)

Fig. S8. Functional characterisation of INS-1 832/3 *Glp1r*$^{-/-}$ SNAP/FLAG-*hGLP1R*^{WT} versus ^{A316T} sublines – extra data. (**A**) Percentage of GLP-1R internalisation over time following stimulation with the indicated agonists in INS-1 832/3 *Glp1r*$^{-/-}$ SNAP/FLAG-*hGLP1R*^{WT} versus ^{A316T} cells; *n* = 3. (**B**) Internalisation AUCs calculated from (**A**). (**C**) Percentage of GLP-1R recycling to the plasma membrane over time following stimulation with the indicated agonists in INS-1 832/3 *Glp1r*$^{-/-}$ SNAP/FLAG-*hGLP1R*^{WT} versus ^{A316T} cells; *n* = 3. (**D**) Recycling AUCs calculated from (**C**). (**E**) Percentage of GLP-1R degradation over time following stimulation with the indicated agonists in INS-1 832/3 *Glp1r*$^{-/-}$ SNAP/FLAG-*hGLP1R*^{WT} versus ^{A316T} cells; *n* = 3. (**F**) Degradation AUCs calculated from (**E**). (**G**) Vehicle-subtracted LgBiT-mini-G_s recruitment to *hGLP1R*^{WT} or ^{A316T}-SmBiT over time in INS-1 832/3 *Glp1r*$^{-/-}$ cells following stimulation with 100 nM of the indicated agonist; *n* = 5. (**H**) LgBiT-mini-G_s recruitment to *hGLP1R*^{WT} or ^{A316T}-SmBiT AUC dose responses to the indicated agonist; *n* = 5. (**I**) Vehicle-subtracted LgBiT-β-arrestin 2 (βarr2) recruitment to *hGLP1R*^{WT} or ^{A316T}-SmBiT over time in INS-1 832/3 *Glp1r*$^{-/-}$ cells following stimulation with 100 nM of the indicated agonist; *n* = 5. (**J**) LgBiT-βarr2 recruitment to *hGLP1R*^{WT} or ^{A316T}-SmBiT AUC dose responses to the indicated agonist; *n* = 5. (**K** to **N**) cAMP responses from cADDis transduced INS-1 832/3 *Glp1r*$^{-/-}$ SNAP/FLAG-*hGLP1R*^{WT} versus ^{A316T} cells in response to 100 nM GLP-1 (**K**), exendin-4 (Ex-4) (**L**), exendin-F1 (Ex-F1) (**M**), or semaglutide (Sema) (**N**), followed by IBMX plus forskolin (FSK) stimulation for maximal responses; *n* = 4. (**O** and **P**) cAMP AUCs (**O**) or ΔAUC (SNAP/FLAG-*hGLP1R*^{A316T} minus ^{WT}) (**P**) calculated from agonist-stimulated periods in (**K** to **N**); *n* = 4. Data are mean ± SEM except for ΔAUC *hGLP1R*^{A316T} minus ^{WT} responses which are mean ± 95% confidence interval (CI); *p<0.05; ns, not significant by two-way ANOVA with Sidak's post-hoc tests.

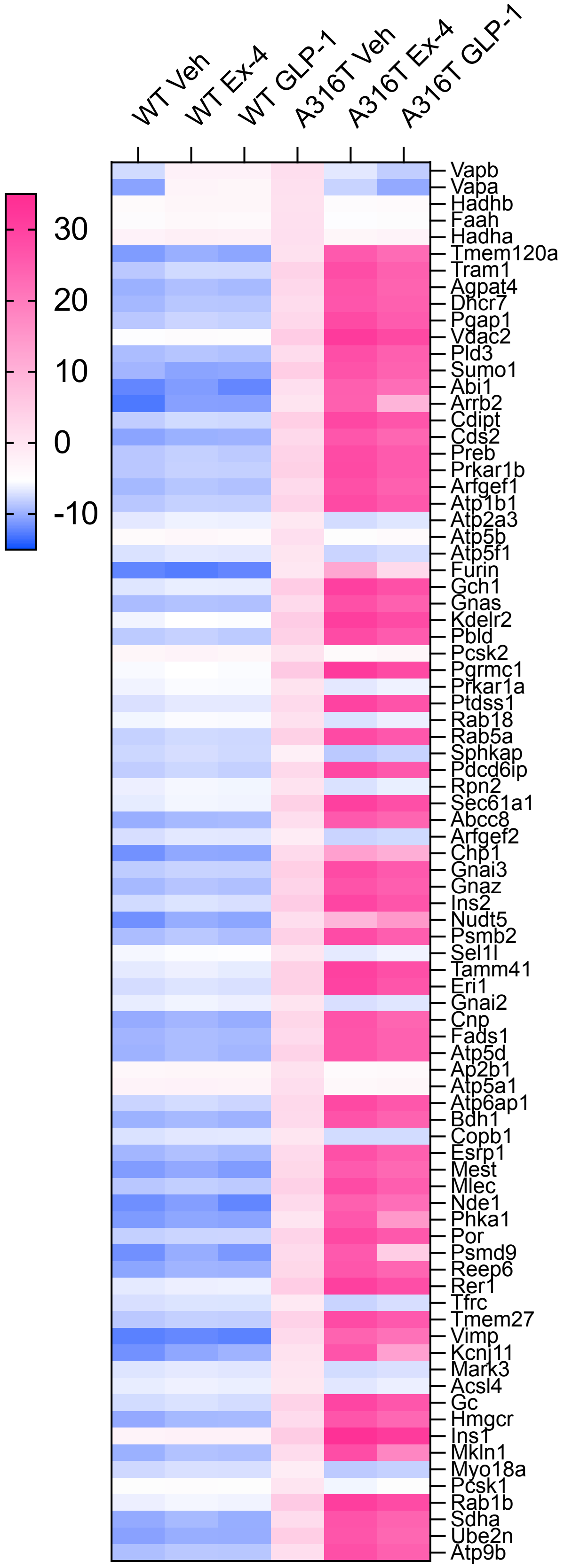

WT Veh
WT Ex-4
WT GLP-1
A316T Veh
A316T Ex-4
A316T GLP-1
30
20
10
0
-10
Vapb
Vapa
Hadhb
Faah
Hadha
Tmem120a
Tram1
Agpat4
Dhcr7
Pgap1
Vdac2
Pld3
Sumo1
Abi1
Arrb2
Cdipt
Cds2
Preb
Prkar1b
Arfgef1
Atp1b1
Atp2a3
Atp5b
Atp5f1
Furin
Gch1
Gnas
Kdelr2
Pbld
Pcsk2
Pgrmc1
Prkar1a
Ptdss1
Rab18
Rab5a
Sphkap
Pdcd6ip
Rpn2
Sec61a1
Abcc8
Arfgef2
Chp1
Gnai3
Gnaz
Ins2
Nudt5
Psmb2
Sel1l
Tamm41
Eri1
Gnai2
Cnp
Fads1
Atp5d
Ap2b1
Atp5a1
Atp6ap1
Bdh1
Copb1
Esrp1
Mest
Mlec
Nde1
Phka1
Por
Psmd9
Reep6
Rer1
Tfrc
Tmem27
Vimp
Kcnj11
Mark3
Acsl4
Gc
Hmgcr
Ins1
Mkln1
Myo18a
Pcsk1
Rab1b
Sdha
Ube2n
Atp9b

Fig. S9. GLP-1R WT versus A316T interactome analysis in INS-1 832/3 *Glp1r*$^{-/-}$ SNAP/FLAG-*hGLP1R*^{WT} versus ^{A316T} cells. Heatmap of GLP-1R interactor enrichment from LC-MS/MS analysis of INS-1 832/3 *Glp1r*$^{-/-}$ SNAP/FLAG-*hGLP1R*^{WT} versus ^{A316T} anti-FLAG co-immunoprecipitates under vehicle (Veh) or 5 min stimulation with 100 nM exendin-4 (Ex-4) or GLP-1; blue, decreased; pink, increased interactor enrichment; LC-MS/MS data analysed in LFQ-Analyst and normalised to hGLP-1R levels for each experimental repeat; colour scale depicting $\log_2$ fold change to WT vehicle centred around overall median value of the experiment; n = 2-4.

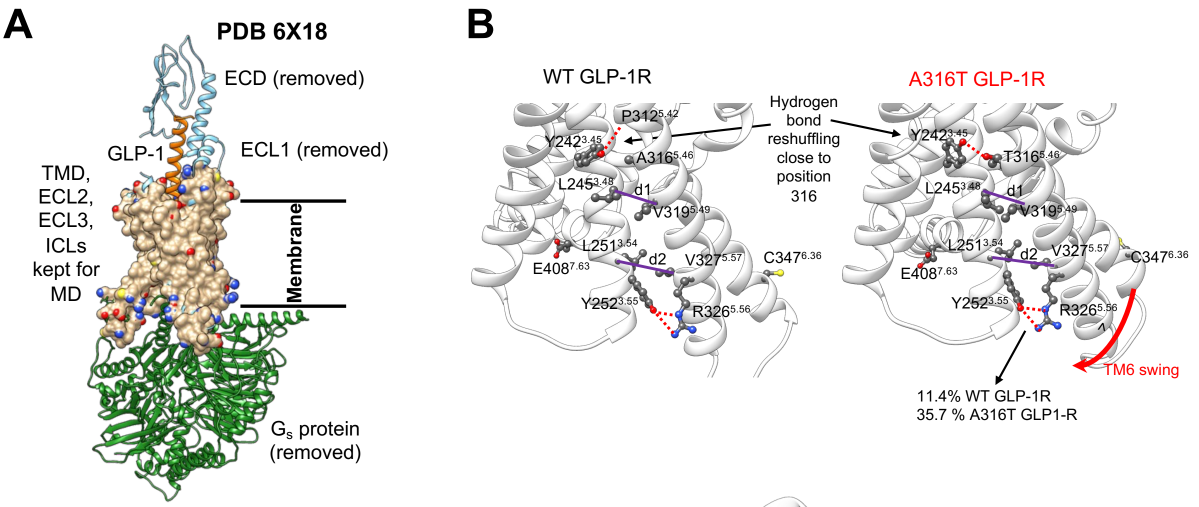

C

Distance	WT GLP-1R	A316T GLP-1R
L245$^{3.48}$-V319$^{5.49}$ (**d1**)	7.58 ± 0.28 Å	8.09 ± 0.34 Å
L251$^{3.54}$-V327$^{5.57}$ (**d2**)	8.91 ± 0.68 Å	7.75 ± 0.48 Å

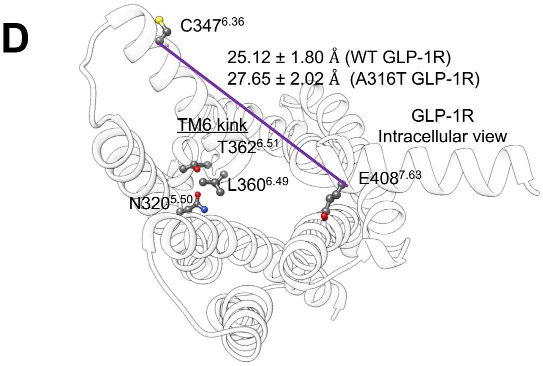

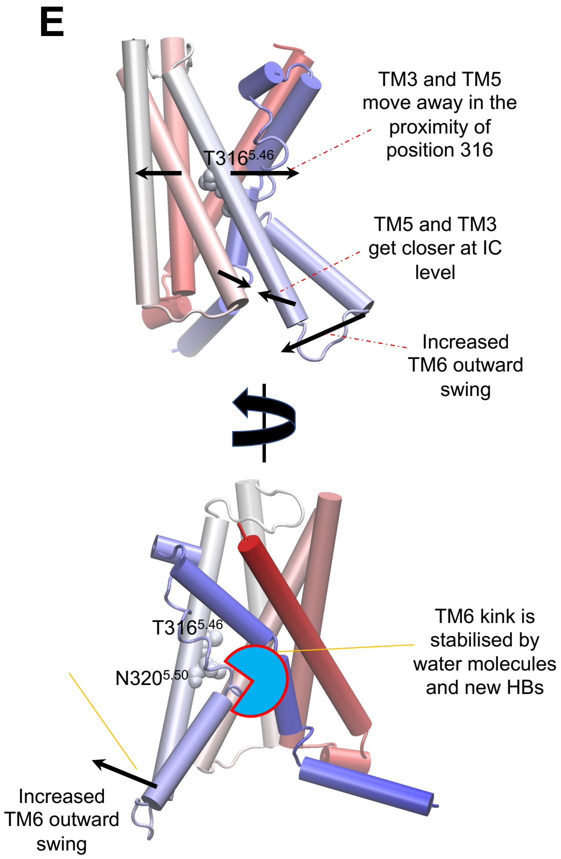

Fig. S10. MD simulations of GLP-1R A316T TMD. (**A**) GLP-1R structure used in MD simulations: only the TMD, ECL2, ECL3, and ICLs were retained to highlight the subtle allosteric effect exerted by A316T. (**B**) Comparison between GLP-1R WT and A316T TMD. The side chain of Y242$^{3.45}$ forms a persistent H-bond with the backbone of P312$^{5.42}$ in WT GLP-1R (left), while in A316T the side chain of T316$^{5.46}$ engages Y242$^{3.45}$ (right); as a result of this H-bond reshuffling, the TM3 - TM5 interhelical distance at the L245$^{3.48}$ - V319$^{5.49}$ level (d1) increases from 7.58 ± 0.28 Å to 8.09 ± 0.34 Å, while the TM3 - TM5 interhelical distance at the L251$^{3.54}$ - V327$^{5.57}$ level (d2) decreases from 8.91 ± 0.68 Å to 7.75 ± 0.48 Å; this is accompanied by a stabilisation of the intracellular side of TM5 due to a more persistent H-bond between Y252$^{3.55}$ and R326$^{5.56}$. (**C**) TM5 - TM6 interhelical distances, d1 and d2. (**D**) In A316T, TM6 dynamics is accentuated and the characteristic, active state opening of TM6 (TM6 swing) increases by ~2.5 Å compared to WT [25.12 ± 1.80 Å (WT GLP-1R) *versus* 27.65 ± 2.02 Å (A316T GLP-1R)]. (**E**) Proposed dynamic model for the enhanced constitutive activity of GLP-1R A316T.

Supplementary Tables

**Residue 1**	**Residue 2**	**WT GLP-1R TMD** (% MD frames)	**A316T GLP-1R TMD** (% MD frames)	**WT GLP-1R: GLP-1** (% MD frames)	**A316T GLP-1R: GLP-1** (% MD frames)	**WT GLP-1R: Ex-F1:Gα_s** (% MD frames)	**A316T GLP-1R: Ex-F1:Gα_s** (% MD frames)
**Y242$^{3.45}$ (sc)**	**A/T316$^{5.46}$ (bb/sc)**	0.0	85.3	0.0	41.8	0.0	37.7
**Y242$^{3.45}$ (sc)**	**P312$^{5.42}$ (bb)**	95.2	2.2	93.3	46.1	87.4	7.9
**A/T316$^{5.46}$ (bb/sc)**	**P312$^{5.42}$ (bb)**	0.0	97.2	0.0	49.6	0.0	86.9
**A/T316$^{5.46}$ (bb/sc)**	**I313$^{5.43}$ (bb)**	0.0	1.6	0.0	47.2	0.0	8.3

sc =side chain; bb=backbone

Table S1: H-bond reshuffling close to position 316. WT GLP-1R and A316T were simulated in 3 different configurations: the isolated TMD in the absence of any agonist, in complex only with the endogenous agonist GLP-1, and in complex with exendin-F1 (Ex-F1) and $G\alpha_s$.

**Residue 1**	**Residue 2**	**WT GLP-1R TMD** (% MD frames)	**A316T GLP-1R TMD** (% MD frames)	**WT GLP-1R: GLP-1** (% MD frames)	**A316T GLP-1R: GLP-1** (% MD frames)	**WT GLP-1R: Ex-F1:$G\alpha_s$** (% MD frames)	**A316T GLP-1R: Ex-F1:$G\alpha_s$** (% MD frames)
**A/T316$^{5.46}$**	**Y241$^{3.44}$**	12.2	2.0	3.1	29.9	11.2	0.8
**A/T316$^{5.46}$**	**N320$^{5.50}$**	3.9	1.1	0.6	5.2	3.4	1.1

Table S2: Water-mediated interactions involving position 316. WT GLP-1R and A316T were simulated in 3 different configurations: the isolated TMD in the absence of any agonist, in complex only with the endogenous agonist GLP-1, and in complex with exendin-F1 (Ex-F1) and $G\alpha_s$.

**Residue 1**	**Residue 2**	**WT GLP-1R TMD** (% MD frames)	**A316T GLP-1R TMD** (% MD frames)	**WT GLP-1R: GLP-1** (% MD frames)	**A316T GLP-1R: GLP-1** (% MD frames)	**WT GLP-1R: Ex-F1:$G\alpha_s$** (% MD frames)	**A316T GLP-1R: Ex-F1:$G\alpha_s$** (% MD frames)
**N320$^{5.50}$ (sc)**	**L360$^{6.49}$ (bb)**	68.5	30.6	79.6	35.6	65.4	54.8
**N320 $^{5.50}$ (wb)**	**L360$^{6.49}$ (wb)**	19.5	49.3	12.2	26.8	24.2	31.7
**N320$^{5.50}$ (wb)**	**G361$^{6.50}$ (wb)**	5.3	19.5	5.9	10.3	10.9	9.8

sc =side chain; bb=backbone; wb=water bridge

Table S3: H-bond and water-mediated interactions involving N320$^{5.50}$ and TM6 kink. WT GLP-1R and A316T were simulated in 3 different configurations: the isolated TMD in the absence of any agonist, in complex only with the endogenous agonist GLP-1, and in complex with exendin-F1 (Ex-F1) and Gα_s.

**Residue 1**	**WT GLP-1R TMD** (% MD frames)	**A316T GLP-1R TMD** (% MD frames)	**WT GLP-1R: GLP-1** (% MD frames)	**A316T GLP-1R: GLP-1** (% MD frames)	**WT GLP-1R: Ex-F1:Gα_s** (% MD frames)	**A316T GLP-1R: Ex-F1:Gα_s** (% MD frames)
**A/T316$^{5.46}$**	1.1	1.8	0.8	6.8	1.5	5.9
**N320$^{5.50}$**	2.6	4.4	3.8	15.3	4.4	11.8
**L360$^{6.49}$**	2.3	3.8	2.7	11.3	3.0	7.3
**G361$^{6.50}$**	2.4	3.1	9.9	13.2	6.7	14.9

Table S4: Water molecules maximum occupancy nearby positions 316, 320, 360 and 361. The occupancy of the water molecule with the highest residence time is reported as % of the total MD frames.

**A316T *versus* WT GLP-1R**				
	**Vehicle**	**GLP-1**	**Exendin-4**	**Exendin-F1**
**Glucose tolerance**	↑↑ (0 hr); ↑↑ (6 hr)	-	↓↓ (0 hr); ↓↓ (6 hr)	↓↓ (0 hr); ≈ (6 hr)
**Plasma insulin**	↑ (0 hr); ↑↑ (6 hr)	-	↓ (0 hr); ↓↓ (6 hr)	-
**Surface level**	↓↓	-	-	-
**cAMP generation**	↑	-	↓	↑
**Insulin secretion**	↑↑ (0hr); ↑↑ (6 hr)	-	↓↓ (0 hr); ↓ (6 hr)	↓↓ (0 hr); ↓ (6 hr)
**Glucagon secretion**	↓↓			
**Internalisation**	↑↑	↑↑	≈	≈
**Recycling**	-	↓↓	≈	≈
**Degradation**	↑↑	≈	≈	≈

**Ca^{2+} mobilisation**	↑↑	≈	≈	↑↑
**Mini-G_s recruitment**	↑↑	≈	≈	↑
**β-arrestin 2 recruitment**	≈	≈	≈	↓
**Endosomal activity**	↑	↑	↓	-
**Plasma membrane activity**	≈	≈	≈	-
**ECD opening**	≈	↑↑	-	-
**Lipid raft Recruitment**	↑↑	≈	↓↓	-
**Membrane diffusion**	↓↓	-	≈	-
**Ubiquitination**	≈	-	↑↑	-

Table S5: Summary of GLP-1R WT *versus* A316T effects. ↓ reduced; ↑ increased; two arrows, significant; one arrow, tendency; ≈ unchanged; - untested.

Arrival date	Donor Age	Sex	BMI (kg/m^2)
24.11.21	73	Female	32
08.12.21	46	Female	31.9
10.12.21	67	Male	29.7
08.02.22	64	Male	26.7

Table S6: Human islet donor characteristics.

Primer Name	Sequence
PCR forward primer	5'- GCAGTACTGTGTGTGGCGGCCAATTACTACT- 3'
PCR reverse primer	3'- GTCCATCACAAAGGCAAAGATGACCTCA-5'
A316T sequencing primer	5'- GTACCTGTACACACTGCTGG-3'

Table S7: *hG*LP1R$^{A316T/A36T}$ mouse genotyping primers.

Gene	Forward (5'→ 3')	Reverse (5'→ 3')
Actb	CACTGTCGAGTCGCGTCC	TCATCCATGGCGAACTGGTG
Mafa	CTTCAGCAAGGAGGAGGTCATC	CGTAGCCGCGGTTCTTGA
Acot7	AGATGATTGAGGAGGCCGG	ACAGCGCTCCCCATTCTG
LdhA	ATGAAGGACTTGGCGGATGA	ATCTCGCCCTTGAGTTTGTCTT
Slc16a1	GCTTGGTGACCATTGTGGAAT	CCCAGTACGTGTATTTGTAGTCTCCAT
Gcg	CCAAGAGGAACCGGAACAAC	CCTTCAGCATGCCTCTCAAAT
Ins1	GCTGGTGGGCATCCAGTAA	AATGACCTGCTTGCTGATGGT
Kcnj11	CACGGCGGGATAAGTCTACCT	AATCATTTGCCCCCTTCTTGT

Table S8: Primers used for qPCR.

Imaging	GLP-1:GLP-1R(A316T):DN-Gα_s:nb35
Magnification (*x*)	120,000
Accelerating voltage (kV)	200
Total electron dose ($e^-/Å^2$)	50
Exposure time (sec)	7.66
Frames	N/A (EER)
Pixel size (Å)	0.86
Detector	Falcon 4
Acquisition routine	Aberration-free image shift (AFIS) fast acquisition
**Processing**	
Symmetry imposed	C1
Initial particle images	1,667,111
Final particle images	330,000
Resolution (Å) @ FSC = 0.143	3.4
**Molecular refinement**	
Initial model	PDB 6X18
Chains	6
Non-hydrogen atoms	18487
Protein residues	1172
Waters	0
Ligands	0

Bonds (RMSD)	
Bond length (Å)	0.009
Bond angles (°)	1.28
**Validation**	
MolProbity score	1.23
Clashscore	2.71
Rotamer outliers (%)	0.80
CaBLAM outliers (%)	0.09
**Ramachandran statistics**	
Favoured (%)	97.05
Allowed (%)	2.95
Disallowed (%)	0

Table S9: Data collection, processing and refinement statistics for cryo-EM of GLP1-bound GLP-1R (A316T):DN-Gα_s:Nb35 complex.

Movie S1: GLP-1R environment surrounding position 316$^{5.46}$ in GLP-1R (WT):GLP-1, GLP-1R (A316T):GLP-1, and GLP-1R (A316T):exendin-F1. T316$^{5.46}$ rotameric states 1 and 2 and a side-by-side comparison of the GLP-1R environment in GLP-1R (WT):GLP-1, GLP-1R (A316T):GLP-1, and GLP-1R (A316T):exendin-F1 (Ex-F1):Gα_s near positions 316$^{5.46}$ (A/T), N320$^{5.50}$ and TM6 kink. Y242$^{3.45}$, N320$^{5.50}$, L360$^{6.49}$, and G361$^{6.60}$ are shown as sticks, with position 316$^{5.46}$ (A/T) shown as thicker sticks. Water molecules within 3.5 Å of position 316$^{5.46}$ and N320$^{5.50}$ are shown as red spheres; H-bonds are represented by dashed lines; GLP-1R backbone is shown as transparent white ribbon; GLP-1 is in orange and Ex-F1 in green ribbons.

REFERENCES

1. D. Tornehave, P. Kristensen, J. Rømer, L. B. Knudsen, R. S. Heller, Expression of the GLP-1 receptor in mouse, rat, and human pancreas. *J. Histochem. Cytochem.* **56**, 841–851 (2008).

2. J. Ast, D. Nasteska, N. H. F. Fine, D. J. Nieves, Z. Koszegi, Y. Lanoiselée, F. Cuozzo, K. Viloria, A. Bacon, N. T. Luu, P. N. Newsome, D. Calebiro, D. M. Owen, J. Broichhagen, D. J. Hodson, Revealing the tissue-level complexity of endogenous glucagon-like peptide-1 receptor expression and signaling. *Nat. Commun.* **14**, 301 (2023).

3. C. Pyke, R. S. Heller, R. K. Kirk, C. Orskov, S. Reedtz-Runge, P. Kaastrup, A. Hvelplund, L. Bardram, D. Calatayud, L. B. Knudsen, GLP-1 receptor localization in monkey and human tissue: Novel distribution revealed with extensively validated monoclonal antibody. *Endocrinology* **155**, 1280–1290 (2014).

4. D. Donnelly, The structure and function of the glucagon-like peptide-1 receptor and its ligands. *Br. J. Pharmacol.* **166**, 27–27 (2012).

5. J. J. Holst, C. Ørskov, The incretin approach for diabetes treatment: Modulation of islet hormone release by GLP-1 agonism. *Diabetes* **53**, S197–S204 (2004).

6. W. T. Garvey, R. L. Batterham, M. Bhatta, S. Buscemi, L. N. Christensen, J. P. Frias, E. Jodar, K. Kandler, G. Rigas, T. A. Wadden, S. Wharton, STEP 5 Study Group, Two-year effects of semaglutide in adults with overweight or obesity: The STEP 5 trial. *Nat. Med.* **28**, 2083–2091 (2022).

7. M. Davies, L. Faerch, O. K. Jeppesen, A. Pakseresht, S. D. Pedersen, L. Perreault, J. Rosenstock, I. Shimomura, A. Viljoen, T. A. Wadden, I. Lingvay, STEP 5 Study Group, Semaglutide 2.4 mg once a week in adults with overweight or obesity, and type 2 diabetes (STEP 2): A randomised, double-blind, double-dummy, placebo-controlled, phase 3 trial. *Lancet* **397**, 971–984 (2021).

8. Y. Zhang, B. Sun, D. Feng, H. Hu, M. Chu, Q. Qu, J. T. Tarrasch, S. Li, T. Sun Kobilka, B. K. Kobilka, G. Skiniotis, Cryo-EM structure of the activated GLP-1 receptor in complex with G protein. *Nature* **546**, 248–248 (2017).

9. G. Deganutti, Y. L. Liang, X. Zhang, M. Khoshouei, L. Clydesdale, M. J. Belousoff, H. Venugopal, T. T. Truong, A. Glukhova, A. N. Keller, K. J. Gregory, K. Leach, A. Christopoulos, R. Danev, C. A. Reynolds, P. Zhao, P. M. Sexton, D. Wootten, Dynamics of GLP-1R peptide agonist engagement are correlated with kinetics of G protein activation. *Nat. Commun.* **13**, 92 (2022).

10. G. Kang, J. W. Joseph, O. G. Chepurny, M. Monaco, M. B. Wheeler, J. L. Bos, F. Schwede, H. G. Genieser, G. G. Holz, Epac-selective cAMP analog 8-pCPT-2'-O-Me-cAMP as a stimulus for Ca^{2+}-induced Ca^{2+} release and exocytosis in pancreatic beta-cells. *J. Biol. Chem.* **278**, 8279–8285 (2003).

11. X.-Y. Chen, L. Chen, W. Yang, A.-M. Xie, GLP-1 suppresses feeding behaviors and modulates neuronal electrophysiological properties in multiple brain regions. *Front. Mol. Neurosci.* **14**, 793004 (2021).

12. B. Wang, J. Zhong, H. Lin, Z. Zhao, Z. Yan, H. He, Y. Ni, D. Liu, Z. Zhu, Blood pressure-lowering effects of GLP-1 receptor agonists exenatide and liraglutide: A meta-analysis of clinical trials. *Diabetes Obes. Metab.* **15**, 737–749 (2013).

13. A. Goud, J. Zhong, M. Peters, R. D. Brook, S. Rajagopalan, GLP-1 agonists and blood pressure: A review of the evidence. *Curr. Hypertens. Rep.* **18**, 16 (2016).

14. T. Rieg, M. Gerasimova, F. Murray, T. Masuda, T. Tang, M. Rose, D. J. Drucker, V. Vallon, Natriuretic effect by exendin-4, but not the DPP-4 inhibitor alogliptin, is mediated via the GLP-1 receptor and preserved in obese type 2 diabetic mice. *Am. J. Physiol. Renal. Physiol.* **303**, F963–F971 (2012).

15. R. O. Crajoinas, F. T. Oricchio, T. D. Pessoa, B. P. M. Pacheco, L. M. A. Lessa, G. Malnic, A. C. C. Girardi, Mechanisms mediating the diuretic and natriuretic actions of the incretin hormone glucagon-like peptide-1. *Am. J. Physiol. Renal. Physiol.* **301**, 355–363 (2011).

16. H. H. Hansen, K. Fabricius, P. Barkholt, M. L. Niehoff, J. E. Morley, J. Jelsing, C. Pyke, L. B. Knudsen, S. A. Farr, N. Vrang, The GLP-1 receptor agonist liraglutide improves memory

function and increases hippocampal CA1 neuronal numbers in a senescence-accelerated mouse model of Alzheimer's disease. *J. Alzheimers Dis.* **46**, 877–888 (2015).

17. M. J. During, L. Cao, D. S. Zuzga, J. S. Francis, H. L. Fitzsimons, X. Jiao, R. J. Bland, M. Klugmann, W. A. Banks, D. J. Drucker, C. N. Haile, Glucagon-like peptide-1 receptor is involved in learning and neuroprotection. *Nat. Med.* **9**, 1173–1179 (2003).

18. N. Reich, C. Hölscher, The neuroprotective effects of glucagon-like peptide 1 in Alzheimer's and Parkinson's disease: An in-depth review. *Front. Neurosci.* **16**, 970925 (2022).

19. P. Ghosh, R. A. Fontanella, L. Scisciola, A. Pesapane, F. Taktaz, M. Franzese, A. Puocci, A. Ceriello, F. Prattichizzo, M. R. Rizzo, G. Paolisso, M. Barbieri, Targeting redox imbalance in neurodegeneration: Characterizing the role of GLP-1 receptor agonists. *Theranostics* **13**, 4872–4884 (2023).

20. L. A. Abushamat, P. A. Shah, R. H. Eckel, S. A. Harrison, D. Barb, The emerging role of glucagon-like peptide-1 receptor agonists for the treatment of metabolic dysfunction-associated steatohepatitis. *Clin. Gastroenterol. Hepatol.* **22**, 1565–1574 (2024).

21. A. Y. Dawed, A. Mari, A. Brown, T. J. McDonald, L. Li, S. Wang, M.-G. Hong, S. Sharma, N. R. Robertson, A. Mahajan, X. Wang, M. Walker, S. Gough, L. M.'t Hart, K. Zhou, I. Forgie, H. Ruetten, I. Pavo, P. Bhatnagar, A. G. Jones, E. R. Pearson, DIRECT consortium, Pharmacogenomics of GLP-1 receptor agonists: A genome-wide analysis of observational data and large randomised controlled trials. *Lancet Diabetes Endocrinol.* **11**, 33–41 (2023).

22. A. S. Hauser, S. Chavali, I. Masuho, L. J. Jahn, K. A. Martemyanov, D. E. Gloriam, M. M. Babu, Pharmacogenomics of GPCR drug targets. *Cell* **172**, 41–54.e19 (2018).

23. J. Wessel, A. Y. Chu, S. M. Willems, S. Wang, H. Yaghootkar, J. A. Brody, M. Dauriz, M.-F. Hivert, S. Raghavan, L. Lipovich, B. Hidalgo, K. Fox, J. E. Huffman, P. An, Y. Lu, L. J. Rasmussen-Torvik, N. Grarup, M. G. Ehm, L. Li, A. S. Baldridge, A. Stančáková, R. Abrol, C. Besse, A. Boland, J. Bork-Jensen, M. Fornage, D. F. Freitag, M. E. Garcia, X. Guo, K. Hara, A. Isaacs, J. Jakobsdottir, L. A. Lange, J. C. Layton, M. Li, J. H. Zhao, K. Meidtner, A. C. Morrison, M. A. Nalls, M. J. Peters, M. Sabater-Lleal, C. Schurmann, A. Silveira, A. V.

Smith, L. Southam, M. H. Stoiber, R. J. Strawbridge, K. D. Taylor, T. V. Varga, K. H. Allin, N. Amin, J. L. Aponte, T. Aung, C. Barbieri, N. A. Bihlmeyer, M. Boehnke, C. Bombieri, D. W. Bowden, S. M. Burns, Y. Chen, Y.-D. I. Chen, C.-Y. Cheng, A. Correa, J. Czajkowski, A. Dehghan, G. B. Ehret, G. Eiriksdottir, S. A. Escher, A.-E. Farmaki, M. Frånberg, G. Gambaro, F. Giulianini, W. A. Goddard III, A. Goel, O. Gottesman, M. L. Grove, S. Gustafsson, Y. Hai, G. Hallmans, J. Heo, P. Hoffmann, M. K. Ikram, R. A. Jensen, M. E. Jørgensen, T. Jørgensen, M. Karaleftheri, C. C. Khor, A. Kirkpatrick, A. T. Kraja, J. Kuusisto, E. M. Lange, I. T. Lee, W.-J. Lee, A. Leong, J. Liao, C. Liu, Y. Liu, C. M. Lindgren, A. Linneberg, G. Malerba, V. Mamakou, E. Marouli, N. M. Maruthur, A. Matchan, R. McKean-Cowdin, O. M. Leod, G. A. Metcalf, K. L. Mohlke, D. M. Muzny, I. Ntalla, N. D. Palmer, D. Pasko, A. Peter, N. W. Rayner, F. Renström, K. Rice, C. F. Sala, B. Sennblad, I. Serafetinidis, J. A. Smith, N. Soranzo, E. K. Speliotes, E. A. Stahl, K. Stirrups, N. Tentolouris, A. Thanopoulou, M. Torres, M. Traglia, E. Tsafantakis, S. Javad, L. R. Yanek, E. Zengini, D. M. Becker, J. C. Bis, J. B. Brown, L. A. Cupples, T. Hansen, E. Ingelsson, A. J. Karter, C. Lorenzo, R. A. Mathias, J. M. Norris, G. M. Peloso, W. H.-H. Sheu, D. Toniolo, D. Vaidya, R. Varma, L. E. Wagenknecht, H. Boeing, E. P. Bottinger, G. Dedoussis, P. Deloukas, E. Ferrannini, O. H. Franco, P. W. Franks, R. A. Gibbs, V. Gudnason, A. Hamsten, T. B. Harris, A. T. Hattersley, C. Hayward, A. Hofman, J.-H. Jansson, C. Langenberg, L. J. Launer, D. Levy, B. A. Oostra, C. J. O'Donnell, S. O'Rahilly, S. Padmanabhan, J. S. Pankow, O. Polasek, M. A. Province, S. S. Rich, P. M. Ridker, I. Rudan, M. B. Schulze, B. H. Smith, A. G. Uitterlinden, M. Walker, H. Watkins, T. Y. Wong, E. Zeggini, EPIC-Inter Act Consortium, M. Laakso, I. B. Borecki, D. I. Chasman, O. Pedersen, B. M. Psaty, E. S. Tai, C. M. van Duijn, N. J. Wareham, D. M. Waterworth, E. Boerwinkle, W. H. L. Kao, J. C. Florez, R. J. F. Loos, J. G. Wilson, T. M. Frayling, D. S. Siscovick, J. Dupuis, J. I. Rotter, J. B. Meigs, R. A. Scott, M. O. Goodarzi, Low-frequency and rare exome chip variants associate with fasting glucose and type 2 diabetes susceptibility. *Nat. Commun.* **6**, 5897 (2015).

24. R. A. Scott, D. F. Freitag, L. Li, A. Y. Chu, P. Surendran, R. Young, N. Grarup, A. Stancáková, Y. Chen, T. V. Varga, H. Yaghootkar, J. Luan, J. H. Zhao, S. M. Willems, J. Wessel, S. Wang, N. Maruthur, K. Michailidou, A. Pirie, S. J. van der Lee, C. Gillson, A. A. Al Olama, P. Amouyel, L. Arriola, D. Arveiler, I. Aviles-Olmos, B. Balkau, A. Barricarte, I. Barroso, S. B. Garcia, J. C. Bis, S. Blankenberg, M. Boehnke, H. Boeing, E. Boerwinkle, I. B. Borecki, J. Bork-Jensen, S. Bowden, C. Caldas, M. Caslake, CVD50 consortium, L. A.

Cupples, C. Cruchaga, J. Czajkowski, M. den Hoed, J. A. Dunn, H. M. Earl, G. B. Ehret, E. Ferrannini, J. Ferrieres, T. Foltynie, I. Ford, N. G. Forouhi, F. Gianfagna, C. Gonzalez, S. Grioni, L. Hiller, J.-H. Jansson, M. E. Jørgensen, J. W. Jukema, R. Kaaks, F. Kee, N. D. Kerrison, T. J. Key, J. Kontto, Z. Kote-Jarai, A. T. Kraja, K. Kuulasmaa, J. Kuusisto, A. Linneberg, C. Liu, G. Marenne, K. L. Mohlke, A. P. Morris, K. Muir, M. Müller-Nurasyid, P. B. Munroe, C. Navarro, S. F. Nielsen, P. M. Nilsson, B. G. Nordestgaard, C. J. Packard, D. Palli, S. Panico, G. M. Peloso, M. Perola, A. Peters, C. J. Poole, J. R. Quirós, O. Rolandsson, C. Sacerdote, V. Salomaa, M.-J. Sánchez, N. Sattar, S. J. Sharp, R. Sims, N. Slimani, J. A. Smith, D. J. Thompson, S. Trompet, R. Tumino, D. L. van der A, Y. T. van der Schouw, J. Virtamo, M. Walker, K. Walter, GERAD_EC Consortium, Neurology Working Group of the Cohorts for Heart, Aging Research in Genomic Epidemiology (CHARGE), Alzheimer's Disease Genetics Consortium, Pancreatic Cancer Cohort Consortium, European Prospective Investigation into Cancer and Nutrition–Cardiovascular Disease (EPIC-CVD), EPIC-Inter Act, J. E. Abraham, L. T. Amundadottir, J. L. Aponte, A. S. Butterworth, J. Dupuis, D. F. Easton, R. A. Eeles, J. Erdmann, P. W. Franks, T. M. Frayling, T. Hansen, J. M. M. Howson, T. Jørgensen, J. Kooner, M. Laakso, C. Langenberg, M. I. McCarthy, J. S. Pankow, O. Pedersen, E. Riboli, J. I. Rotter, D. Saleheen, N. J. Samani, H. Schunkert, P. Vollenweider, S. O'Rahilly, CHARGE consortium, CHD Exome+ Consortium, CARDIOGRAM Exome Consortium, P. Deloukas, J. Danesh, M. O. Goodarzi, S. Kathiresan, J. B. Meigs, M. G. Ehm, N. J. Wareham, D. M. Waterworth, Genomic approach to therapeutic target validation identifies a glucose-lowering GLP1R variant protective for coronary heart disease. *Sci. Transl. Med.* **8**, 341ra376 (2016).

25. V. Lagou, L. Jiang, A. Ulrich, L. Zudina, K. S. G. González, Z. Balkhiyarova, A. Faggian, J. G. Maina, S. Chen, P. V. Todorov, S. Sharapov, A. David, L. Marullo, R. Mägi, R.-M. Rujan, E. Ahlqvist, G. Thorleifsson, H. Gao, E. Evangelou, B. Benyamin, R. A. Scott, A. Isaacs, J. H. Zhao, S. M. Willems, T. Johnson, C. Gieger, H. Grallert, C. Meisinger, M. Müller-Nurasyid, R. J. Strawbridge, A. Goel, D. Rybin, E. Albrecht, A. U. Jackson, H. M. Stringham, I. R. Corrêa Jr., E. Farber-Eger, V. Steinthorsdottir, A. G. Uitterlinden, P. B. Munroe, M. J. Brown, J. Schmidberger, O. Holmen, B. Thorand, K. Hveem, T. Wilsgaard, K. L. Mohlke, Z. Wang, G. W. A.-P. A. Consortium, A. Shmeliov, M. den Hoed, R. J. F. Loos, W. Kratzer, M. Haenle, W. Koenig, B. O. Boehm, T. M. Tan, A. Tomas, V. Salem, I. Barroso, J. Tuomilehto, M. Boehnke, J. C. Florez, A. Hamsten, H. Watkins, I. Njølstad,

H.-E. Wichmann, M. J. Caulfield, K.-T. Khaw, C. M. van Duijn, A. Hofman, N. J. Wareham, C. Langenberg, J. B. Whitfield, N. G. Martin, G. Montgomery, C. Scapoli, I. Tzoulaki, P. Elliott, U. Thorsteinsdottir, K. Stefansson, E. L. Brittain, M. I. McCarthy, P. Froguel, P. M. Sexton, D. Wootten, L. Groop, J. Dupuis, J. B. Meigs, G. Deganutti, A. Demirkan, T. H. Pers, C. A. Reynolds, Y. S. Aulchenko, M. A. Kaakinen, B. Jones, I. Prokopenko, Meta-Analysis of Glucose, Insulin-Related Traits Consortium (MAGIC), GWAS of random glucose in 476,326 individuals provide insights into diabetes pathophysiology, complications and treatment stratification. *Nat. Genet.* **55**, 1448–1448 (2023).

26. D. Handley, S. Almansoori, M. S. Sato, H. Amin, S. Alsters, H. Chahal, S. Purkayastha, K. G. Murphy, M. v. Haelst, C. W. l. Roux, T. Tan, R. G. Walters, F. Drenos, A. I. Blakemore, Rare variants of the glucagon-like peptide-1 receptor (GLP1R) gene are overrepresented in a severe obesity cohort and associated with type 2 diabetes in the UK Biobank. medRxiv 23290347 [Preprint] (2023). 2023.05.22.23290347.

27. W. Li, P. Li, R. Li, Z. Yu, X. Sun, G. Ji, X. Yang, L. Zhu, S. Zhu, GLP1R single-nucleotide polymorphisms rs3765467 and rs10305492 affect β cell insulin secretory capacity and apoptosis through GLP-1. *DNA Cell Biol.* **39**, 1700–1710 (2020).

28. C. Koole, D. Wootten, J. Simms, C. Valant, L. J. Miller, A. Christopoulos, P. M. Sexton, Polymorphism and ligand dependent changes in human glucagon-like peptide-1 receptor (GLP-1R) function: Allosteric rescue of loss of function mutation. *Mol. Pharmacol.* **80**, 486–497 (2011).

29. L. El Eid, C. A. Reynolds, A. Tomas, B. Jones, Biased agonism and polymorphic variation at the GLP-1 receptor: Implications for the development of personalised therapeutics. *Pharmacol. Res.* **184**, 106411–106411 (2022).

30. W. Gao, L. Liu, E. Huh, F. Gbahou, E. Cecon, M. Oshima, L. Houzé, P. Katsonis, A. Hegron, Z. Fan, G. Hou, G. Charpentier, M. Boissel, M. Derhourhi, M. Marre, B. Balkau, P. Froguel, R. Scharfmann, O. Lichtarge, J. Dam, A. Bonnefond, J. Liu, R. Jockers, Human GLP1R variants affecting GLP1R cell surface expression are associated with impaired glucose control and increased adiposity. *Nat. Metab.* **5**, 1673–1684 (2023).

31. M. O. Goodarzi, J. I. Rotter, Genetics insights in the relationship between type 2 diabetes and coronary heart disease. *Circ. Res.* **126**, 1526–1548 (2020).

32. L. S. Jun, A. D. Showalter, N. Ali, F. Dai, W. Ma, T. Coskun, J. V. Ficorilli, M. B. Wheeler, M. D. Michael, K. W. Sloop, A novel humanized GLP-1 receptor model enables both affinity purification and Cre-LoxP deletion of the receptor. *PLOS ONE* **9**, e93746 (2014).

33. B. Jones, T. Buenaventura, N. Kanda, P. Chabosseau, B. M. Owen, R. Scott, R. Goldin, N. Angkathunyakul, I. R. Correa Jr., D. Bosco, P. R. Johnson, L. Piemonti, P. Marchetti, A. M. J. Shapiro, B. J. Cochran, A. C. Hanyaloglu, A. Inoue, T. Tan, G. A. Rutter, A. Tomas, S. R. Bloom, Targeting GLP-1 receptor trafficking to improve agonist efficacy. *Nat. Commun.* **9**, 1602 (2018).

34. L. Clark, GLP-1 receptor agonists: A review of glycemic benefits and beyond. *JAAPA* **37**, 1–4 (2024).

35. J. Xie, N. M. El Sayed, C. Qi, X. Zhao, C. E. Moore, T. P. Herbert, Exendin-4 stimulates islet cell replication via the IGF1 receptor activation of mTORC1/S6K1. *J. Mol. Endocrinol.* **53**, 105–115 (2014).

36. H. Hui, A. Nourparvar, X. Zhao, R. Perfetti, Glucagon-like peptide-1 inhibits apoptosis of insulin-secreting cells via a cyclic 5′-adenosine monophosphate-dependent protein kinase A- and a phosphatidylinositol 3-kinase-dependent pathway. *Endocrinology* **144**, 1444–1455 (2003).

37. Z. Ling, D. Wu, Y. Zambre, D. Flamez, D. J. Drucker, D. G. Pipeleers, F. C. Schuit, Glucagon-like peptide 1 receptor signaling influences topography of islet cells in mice. *Virchows Arch.* **438**, 382–387 (2001).

38. B. Portha, C. Tourrel-Cuzin, J. Movassat, Activation of the GLP-1 receptor signalling pathway: A relevant strategy to repair a deficient beta-cell mass. *Exp. Diabetes Res.* **2011**, 376509–376509 (2011).

39. S. Shrestha, G. Erikson, J. Lyon, A. F. Spigelman, A. Bautista, J. M. E. Fox, C. Dos Santos, M. Shokhirev, J. P. Cartailler, M. W. Hetzer, P. E. MacDonald, R. A. E. Drigo, Aging compromises human islet beta cell function and identity by decreasing transcription factor activity and inducing ER stress. *Sci. Adv.* **8**, eabo3932 (2022).

40. A. Bartolomé, N. Suda, J. Yu, C. Zhu, J. Son, H. Ding, A. Califano, D. Accili, U. B. Pajvani, Notch-mediated Ephrin signaling disrupts islet architecture and β cell function. *JCI insight* **7**, e157694 (2022).

41. F. Hu, X. Qiu, S. Bu, Pancreatic islet dysfunction in type 2 diabetes mellitus. *Arch. Physiol. Biochem.* **126**, 235–241 (2020).

42. C.-G. Lv, R.-R. Wang, Z.-X. Meng, Molecular mechanism of islet β-cell functional alternations during type 2 diabetes. *Yi chuan* **44**, 840–852 (2022).

43. A. Martinez-Sanchez, T. J. Pullen, P. Chabosseau, Q. Zhang, E. Haythorne, M. C. Cane, M.-S. Nguyen-Tu, S. R. Sayers, G. A. Rutter, Disallowance of *Acot7* in β-cells is required for normal glucose tolerance and insulin secretion. *Diabetes* **65**, 1268–1282 (2016).

44. Z. Zheng, Y. Zong, Y. Ma, Y. Tian, Y. Pang, C. Zhang, J. Gao, Glucagon-like peptide-1 receptor: Mechanisms and advances in therapy. *Signal Transduct. Target. Ther.* **9**, 234 (2024).

45. E. D. Dean, M. Li, N. Prasad, S. N. Wisniewski, A. Von Deylen, J. Spaeth, L. Maddison, A. Botros, L. R. Sedgeman, N. Bozadjieva, O. Ilkayeva, A. Coldren, G. Poffenberger, A. Shostak, M. C. Semich, K. I. Aamodt, N. Phillips, H. Yan, E. Bernal-Mizrachi, J. D. Corbin, K. C. Vickers, S. E. Levy, C. Dai, C. Newgard, W. Gu, R. Stein, W. Chen, A. C. Powers, Interrupted glucagon signaling reveals hepatic α cell axis and role for L-glutamine in α cell proliferation. *Cell Metab.* **25**, 1362–1373 e5 (2017).

46. A. Hamilton, L. Eliasson, J. G. Knudsen, Amino acids and the changing face of the α-cell. *Peptides* **166**, 171039 (2023).

47. B. Havranek, R. Loh, B. Torre, R. Redfield, D. Halegoua-DeMarzio, Glucagon-like peptide-1 receptor agonists improve metabolic dysfunction-associated steatotic liver disease outcomes. *Sci. Rep.* **10**, 4947 (2025).

48. M. Zafer, F. Tavaglione, M. Romero-Gomez, R. Loomba, Review article: GLP-1 receptor agonists and glucagon/GIP/GLP-1 receptor dual or triple agonists-mechanism of action and emerging therapeutic landscape in MASLD. *Aliment. Pharmacol. Ther.* **61**, 1872–1888 (2025).

49. M. J. Concepción-Zavaleta, J. M. Fuentes-Mendoza, J. G. Gonzáles-Yovera, G. Y. Ruvalcaba-Barbosa, L. D. Cura-Rodríguez, J. S. González-Rodríguez, L. A. Concepción-Urteaga, A. I. Pérez-Reyes, J. E. Quiroz-Aldave, J. Paz-Ibarra, Efficacy and safety of anti-obesity drugs in metabolic dysfunction-associated steatotic liver disease: An updated review. *World J. Gastroenterol.* **31**, 111435 (2025).

50. I. Cattani-Cavalieri, J. Margolis, C. Anicolaesei, F. J. Nunez, R. S. Ostrom, Real-time cAMP dynamics in live cells using the fluorescent cAMP difference detector in situ. *J. Vis. Exp.* **22**, https://doi.org/10.3791/66451 (2024).

51. J. Schirra, K. Sturm, P. Leicht, R. Arnold, B. Goke, M. Katschinski, Exendin(9-39)amide is an antagonist of glucagon-like peptide-1(7-36)amide in humans. *J. Clin. Invest.* **101**, 1421–1430 (1998).

52. V. Serre, W. Dolci, E. Schaerer, L. Scrocchi, D. Drucker, S. Efrat, B. Thorens, Exendin-(9-39) is an inverse agonist of the murine glucagon-like peptide-1 receptor: Implications for basal intracellular cyclic adenosine 3′,5′-monophosphate levels and beta-cell glucose competence. *Endocrinology* **139**, 4448–4454 (1998).

53. C. Greenhill, GWAS gives insights into glucose regulation. *Nat. Rev. Endocrinol.* **19**, 684 (2023).

54. M. Benazra, M.-J. Lecomte, C. Colace, A. Müller, C. Machado, S. Pechberty, E. Bricout-Neveu, M. Grenier-Godard, M. Solimena, R. Scharfmann, P. Czernichow, P. Ravassard, A human beta cell line with drug inducible excision of immortalizing transgenes. *Mol. Metab.* **4**, 916–925 (2015).

55. J. Naylor, A. T. Suckow, A. Seth, D. J. Baker, I. Sermadiras, P. Ravn, R. Howes, J. Li, M. R. Snaith, M. P. Coghlan, D. C. Hornigold, Use of CRISPR/Cas9-engineered INS-1 pancreatic β cells to define the pharmacology of dual GIPR/GLP-1R agonists. *Biochem. J.* **473**, 2881–2891 (2016).

56. A. Yamamoto, Y. Tagawa, T. Yoshimori, Y. Moriyama, R. Masaki, Y. Tashiro, Bafilomycin A1 prevents maturation of autophagic vacuoles by inhibiting fusion between autophagosomes and lysosomes in rat hepatoma cell line, H-4-II-E cells. *Cell Struct. Funct.* **23**, 33–42 (1998).

57. Y. Manchanda, L. ElEid, A. I. Oqua, Z. Ramchunder, J. Choi, M. M. Shchepinova, G. A. Rutter, A. Inoue, E. W. Tate, B. Jones, A. Tomas, Engineered mini-G proteins block the internalization of cognate GPCRs and disrupt downstream intracellular signaling. *Sci. Signal.* **17**, eabq7038 (2024).

58. T. Buenaventura, S. Bitsi, W. E. Laughlin, T. Burgoyne, Z. Lyu, A. I. Oqua, H. Norman, E. R. M. Glone, A. S. Klymchenko, I. R. Corrêa Jr., A. Walker, A. Inoue, A. Hanyaloglu, J. Grimes, Z. Koszegi, D. Calebiro, G. A. Rutter, S. R. Bloom, B. Jones, A. Tomas, Agonist-induced membrane nanodomain clustering drives GLP-1 receptor responses in pancreatic beta cells. *PLoS Biol.* **17**, e3000097 (2019).

59. Y. Manchanda, S. Bitsi, S. Chen, J. Broichhagen, J. Bernardino de la Serna, B. Jones, A. Tomas, Enhanced endosomal signaling and desensitization of GLP-1R vs GIPR in pancreatic beta cells. *Endocrinology* **164**, bqad028 (2023).

60. A. I. Oqua, K. Chao, L. E. Eid, L. Casteller, B. P. Baxter, A. Miguéns-Gómez, S. Barg, B. Jones, J. B. de la Serna, S. L. Rouse, A. Tomas, Molecular mapping and functional validation of GLP-1R cholesterol binding sites in pancreatic beta cells. *eLife* **13**, RP101011 (2025).

61. S. Bitsi, L. El Eid, Y. Manchanda, A. I. Oqua, N. Mohamed, B. Hansen, K. Suba, G. A. Rutter, V. Salem, B. Jones, A. Tomas, Divergent acute versus prolonged pharmacological GLP-1R responses in adult β cell–specific β-arrestin 2 knockout mice. *Sci. Adv.* **9**, eadf7737 (2023).

62. M. R. Dores, J. Trejo, Endo-lysosomal sorting of G-protein-coupled receptors by ubiquitin: Diverse pathways for G-protein-coupled receptor destruction and beyond. *Traffic* **20**, 101–109 (2019).

63. G. Austin, A. I. Oqua, L. E. Eid, M. Zhu, Y. Manchanda, P. Peres, H. Coyle, Y. Poliakova, Z. Balkhiyarova, K. Bouzakri, A. Montoya, D. J. Withers, M. Solimena, B. Jones, S. J. Millership, S. Burgold, D. C. A. Gaboriau, E. Majorovits, E. Garlick, M. A. D. R. B. F. Lima, I. Prokopenko, J. Nixon-Abell, A. Müller, A. Tomas, GLP-1R associates with VAPB and SPHKAP at ERMCSs to regulate beta-cell mitochondrial remodelling and function. *Nat. Commun.* **16**, 11010 (2025).

64. X. Zhang, M. J. Belousoff, P. Zhao, A. J. Kooistra, T. T. Truong, S. Y. Ang, C. R. Underwood, T. Egebjerg, P. Šenel, G. D. Stewart, Y. L. Liang, A. Glukhova, H. Venugopal, A. Christopoulos, S. G. B. Furness, L. J. Miller, S. Reedtz-Runge, C. J. Langmead, D. E. Gloriam, R. Danev, P. M. Sexton, D. Wootten, Differential GLP-1R binding and activation by peptide and non-peptide agonists. *Mol. Cell* **80**, 485–500.e7 (2020).

65. Z. Cong, Y.-L. Liang, Q. Zhou, S. Darbalaei, F. Zhao, W. Feng, L. Zhao, H. E. Xu, D. Yang, M.-W. Wang, Structural perspective of class B1 GPCR signaling. *Trends Pharmacol. Sci.* **43**, 321–334 (2022).

66. A. Punjani, D. J. Fleet, 3D variability analysis: Resolving continuous flexibility and discrete heterogeneity from single particle cryo-EM. *J. Struct. Biol.* **213**, 107702 (2021).

67. W. I. Weis, B. K. Kobilka, The molecular basis of G protein-coupled receptor activation. *Annu. Rev. Biochem.* **87**, 897–919 (2018).

68. S. Balasubramanian, Y. Xia, E. Freinkman, M. Gerstein, Sequence variation in G-protein-coupled receptors: Analysis of single nucleotide polymorphisms. *Nucleic Acids Res.* **33**, 1710–1721 (2005).

69. G. O. Austin, A. Tomas, Variation in responses to incretin therapy: Modifiable and non-modifiable factors. *Front. Mol. Biosci.* **10**, 1170181 (2023).

70. C. E. Hinds, E. Peace, S. Chen, I. Davies, L. El Eid, A. Tomas, T. Tan, J. Minnion, B. Jones, S. R. Bloom, Abolishing β-arrestin recruitment is necessary for the full metabolic benefits of G protein-biased glucagon-like peptide-1 receptor agonists. *Diabetes Obes. Metab.* **26**, 65–77 (2024).

71. R. A. Scott, D. F. Freitag, L. Li, A. Y. Chu, P. Surendran, R. Young, N. Grarup, A. Stancáková, Y. Chen, T. V. Varga, H. Yaghootkar, J. Luan, J. H. Zhao, S. M. Willems, J. Wessel, S. Wang, N. Maruthur, K. Michailidou, A. Pirie, S. J. van der Lee, C. Gillson, A. A. Al Olama, P. Amouyel, L. Arriola, D. Arveiler, I. Aviles-Olmos, B. Balkau, A. Barricarte, I. Barroso, S. B. Garcia, J. C. Bis, S. Blankenberg, M. Boehnke, H. Boeing, E. Boerwinkle, I. B. Borecki, J. Bork-Jensen, S. Bowden, C. Caldas, M. Caslake, CVD consortium, L. A. Cupples, C. Cruchaga, J. Czajkowski, M. den Hoed, J. A. Dunn, H. M. Earl, G. B. Ehret, E. Ferrannini, J. Ferrieres, T. Foltynie, I. Ford, N. G. Forouhi, F. Gianfagna, C. Gonzalez, S. Grioni, L. Hiller, J.-H. Jansson, M. E. Jørgensen, J. W. Jukema, R. Kaaks, F. Kee, N. D. Kerrison, T. J. Key, J. Kontto, Z. Kote-Jarai, A. T. Kraja, K. Kuulasmaa, J. Kuusisto, A. Linneberg, C. Liu, G. Marenne, K. L. Mohlke, A. P. Morris, K. Muir, M. Müller-Nurasyid, P. B. Munroe, C. Navarro, S. F. Nielsen, P. M. Nilsson, B. G. Nordestgaard, C. J. Packard, D. Palli, S. Panico, G. M. Peloso, M. Perola, A. Peters, C. J. Poole, J. R. Quirós, O. Rolandsson, C. Sacerdote, V. Salomaa, M.-J. Sánchez, N. Sattar, S. J. Sharp, R. Sims, N. Slimani, J. A. Smith, D. J. Thompson, S. Trompet, R. Tumino, D. L. van der A, Y. T. van der Schouw, J. Virtamo, M. Walker, K. Walter, GERAD_EC Consortium, Neurology Working Group of the Cohorts for Heart, Aging Research in Genomic Epidemiology (CHARGE), Alzheimer's Disease Genetics Consortium, Pancreatic Cancer Cohort Consortium, European Prospective Investigation into Cancer and Nutrition–Cardiovascular Disease (EPIC-CVD), EPIC-Inter Act, J. E. Abraham, L. T. Amundadottir, J. L. Aponte, A. S. Butterworth, J. Dupuis, D. F. Easton, R. A. Eeles, J. Erdmann, P. W. Franks, T. M. Frayling, T. Hansen, J. M. M. Howson, T. Jørgensen, J. Kooner, M. Laakso, C. Langenberg, M. I. McCarthy, J. S. Pankow, O. Pedersen, E. Riboli, J. I. Rotter, D. Saleheen, N. J. Samani, H. Schunkert, P. Vollenweider, S. O'Rahilly, CHARGE consortium, CHD Exome+ Consortium, CARDIOGRAM Exome Consortium, P. Deloukas, J. Danesh, M. O. Goodarzi, S. Kathiresan, J. B. Meigs, M. G. Ehm, N. J. Wareham, D. M. Waterworth, A genomic approach to therapeutic target validation identifies a glucose-lowering GLP1R variant protective for coronary heart disease. *Sci. Transl. Med.* **8**, 341ra376 (2016).

72. S. Fang, F. Cui, D. J. Drucker, Glucagon-like peptide-1 medicines in neurological and psychiatric disorders. *Cell Rep. Med.* **6**, 102511 (2025).

73. B. Sa, A. Maristany, A. Subramaniam, R. Guillen, B. Buonocore, A. Smith, S. E. Oldak, V. Padilla, Psychiatric effects of GLP-1 receptor agonists: A systematic review of emerging evidence. *Diabetes Obes. Metab.* **28**, 50–59 (2026).

74. J. Guo, M. R. Hayes, L. Leggio, E. Oru, L. Rinaman, GLP-1 receptor agonists and research to treat overeating and substance use disorders. *J. Neurosci.* **45**, (2025).

75. M. Saikia, M. M. Holter, L. R. Donahue, I. S. Lee, Q. C. Zheng, J. L. Wise, J. E. Todero, D. J. Phuong, D. Garibay, R. Coch, K. W. Sloop, A. Garcia-Ocana, C. G. Danko, B. P. Cummings, GLP-1 receptor signaling increases PCSK1 and β cell features in human α cells. *JCI insight* **6**, e141851 (2021).

76. T. Katahira, T. Miyatsuka, M. Miura, L. Suzuki, M. Himuro, Y. Nishida, H. Satoh, H. Watada, Conversion of pancreatic α cells into insulin-producing cells modulated by β-cell insufficiency and supplemental insulin administration. *Biochem. Biophys. Res. Commun.* **521**, 178–183 (2020).

77. A. L. Hutchison, F. Tavaglione, S. Romeo, M. Charlton, Endocrine aspects of metabolic dysfunction-associated steatotic liver disease (MASLD): Beyond insulin resistance. *J. Hepatol.* **79**, 1524–1541 (2023).

78. T. M. Cacciottolo, K. Lawler, K. M. Méndez-Acevedo, E. M. de Oliveira, A. Syanda, E. Henning, J. M. Keogh, R. Bounds, M. Smith, D. Ashraf, D. Harman, A. Duckworth, E. M. Godfrey, L. Watson, M. Hoare, B. Jones, A. Baker, T. Rashid, I. S. Farooqi, Glucagon receptor deficiency causes early-onset hepatic steatosis. *Diabetes* **74**, 1787–1801 (2025).

79. L. Liu, Y. Xia, B. Wang, Y. Zhang, Efficacy of incretin-based therapies in patients with metabolic dysfunction-associated steatotic liver disease: An updated systematic review and meta-analysis of randomized controlled trials. *J. Gastroenterol. Hepatol.* **40**, 2659–2673 (2025).

80. R. C. Laker, S. Egolf, S. Will, L. Lantier, O. P. McGuinness, C. Brown, N. Bhagroo, S. Oldham, K. Kuszpit, A. Alfaro, X. Li, T. Kang, G. Pellegrini, A.-C. Andréasson, S. Kajani, S. Sitaula, M. R. Larsen, C. J. Rhodes, GLP-1R/GCGR dual agonism dissipates hepatic steatosis to restore insulin sensitivity and rescue pancreatic beta-cell function in obese male mice. *Nat. Commun.* **16**, 4714 (2025).

81. S. Kajani, R. C. Laker, E. Ratkova, S. Will, C. J. Rhodes, Hepatic glucagon action: Beyond glucose mobilization. *Physiol. Rev.* **104**, 1021–1060 (2024).

82. D. Wootten, C. A. Reynolds, K. J. Smith, J. C. Mobarec, C. Koole, E. E. Savage, K. Pabreja, J. Simms, R. Sridhar, S. G. B. Furness, M. Liu, P. E. Thompson, L. J. Miller, A. Christopoulos, P. M. Sexton, The extracellular surface of the GLP-1 receptor is a molecular trigger for biased agonism. *Cell* **165**, 1632–1643 (2016).

83. X. Zhang, M. J. Belousoff, Y.-L. Liang, R. Danev, P. M. Sexton, D. Wootten, Structure and dynamics of semaglutide- and taspoglutide-bound GLP-1R-Gs complexes. *Cell Rep.* **36**, 109374 (2021).

84. A. Cuzzolin, G. Deganutti, V. Salmaso, M. Sturlese, S. Moro, AquaMMapS: An alternative tool to monitor the role of water molecules during protein-ligand association. *ChemMedChem* **13**, 522–531 (2018).

85. S. M. Clardy, E. J. Keliher, J. F. Mohan, M. Sebas, C. Benoist, D. Mathis, R. Weissleder, Fluorescent exendin-4 derivatives for pancreatic β-cell analysis. *Bioconjug. Chem.* **25**, 171–177 (2014).

86. M. Lucey, T. Ashik, A. Marzook, Y. Wang, J. Goulding, A. Oishi, J. Broichhagen, D. J. Hodson, J. Minnion, Y. Elani, R. Jockers, S. J. Briddon, S. R. Bloom, A. Tomas, B. Jones, Acylation of the incretin peptide exendin-4 directly impacts glucagon-like peptide-1 receptor signaling and trafficking. *Mol. Pharmacol.* **100**, 319–334 (2021).

87. J. Ast, A. Arvaniti, N. H. F. Fine, D. Nasteska, F. B. Ashford, Z. Stamataki, Z. Koszegi, A. Bacon, B. J. Jones, M. A. Lucey, S. Sasaki, D. I. Brierley, B. Hastoy, A. Tomas, G. D'Agostino, F. Reimann, F. C. Lynn, C. A. Reissaus, A. K. Linnemann, E. D'Este, D.

Calebiro, S. Trapp, K. Johnsson, T. Podewin, J. Broichhagen, D. J. Hodson, Super-resolution microscopy compatible fluorescent probes reveal endogenous glucagon-like peptide-1 receptor distribution and dynamics. *Nat. Commun.* **11**, 1–18 (2020).

88. Z. Fang, S. Chen, P. Pickford, J. Broichhagen, D. J. Hodson, I. R. Corrêa Jr., S. Kumar, F. Görlitz, C. Dunsby, P. M. W. French, G. A. Rutter, T. Tan, S. R. Bloom, A. Tomas, B. Jones, The influence of peptide context on signaling and trafficking of glucagon-like peptide-1 receptor biased agonists. *ACS Pharmacol. Transl. Sci.* **3**, 345–360 (2020).

89. B. Jones, E. R. McGlone, Z. Fang, P. Pickford, I. R. Correa Jr., A. Oishi, R. Jockers, A. Inoue, S. Kumar, F. Görlitz, C. Dunsby, P. M. W. French, G. A. Rutter, T. Tan, A. Tomas, S. R. Bloom, Genetic and biased agonist-mediated reductions in beta-arrestin recruitment prolong cAMP signaling at glucagon family receptors. *J. Biol. Chem.* **296**, 100133 (2021).

90. Q. Wan, N. Okashah, A. Inoue, R. Nehme, B. Carpenter, C. G. Tate, N. A. Lambert, Mini G protein probes for active G protein-coupled receptors (GPCRs) in live cells. *J. Biol. Chem.* **293**, 7466–7473 (2018).

91. Y. Shintani, A. Hayata-Takano, K. Moriguchi, T. Nakazawa, Y. Ago, A. Kasai, K. Seiriki, N. Shintani, H. Hashimoto, beta-Arrestin1 and 2 differentially regulate PACAP-induced PAC1 receptor signaling and trafficking. *PLOS ONE* **13**, e0196946 (2018).

92. N. R. Johnston, R. K. Mitchell, E. Haythorne, M. P. Pessoa, F. Semplici, J. Ferrer, L. Piemonti, P. Marchetti, M. Bugliani, D. Bosco, E. Berishvili, P. Duncanson, M. Watkinson, J. Broichhagen, D. Trauner, G. A. Rutter, D. J. Hodson, Beta cell hubs dictate pancreatic islet responses to glucose. *Cell Metab.* **24**, 389–401 (2016).

93. E. Bru-Tari, N. Cobo-Vuilleumier, P. Alonso-Magdalena, R. S. Dos Santos, L. Marroqui, A. Nadal, B. R. Gauthier, I. Quesada, Pancreatic alpha-cell mass in the early-onset and advanced stage of a mouse model of experimental autoimmune diabetes. *Sci. Rep.* **9**, 9515 (2019).

94. I. Miguel-Escalada, S. Bonas-Guarch, I. Cebola, J. Ponsa-Cobas, J. Mendieta-Esteban, G. Atla, B. M. Javierre, D. M. Y. Rolando, I. Farabella, C. C. Morgan, J. Garcia-Hurtado, A. Beucher, I. Moran, L. Pasquali, M. Ramos-Rodriguez, E. V. R. Appel, A. Linneberg, A. P.

Gjesing, D. R. Witte, O. Pedersen, N. Grarup, P. Ravassard, D. Torrents, J. M. Mercader, L. Piemonti, T. Berney, E. J. P. de Koning, J. Kerr-Conte, F. Pattou, I. O. Fedko, L. Groop, I. Prokopenko, T. Hansen, M. A. Marti-Renom, P. Fraser, J. Ferrer, Human pancreatic islet three-dimensional chromatin architecture provides insights into the genetics of type 2 diabetes. *Nat. Genet.* **51**, 1137–1148 (2019).

95. L. Y. Brown, W. Dong, B. Kantor, An improved protocol for the production of lentiviral vectors. *STAR Protoc* **1**, 100152 (2020).

96. T. Peng, K. Thorn, T. Schroeder, L. Wang, F. J. Theis, C. Marr, N. Navab, A BaSiC tool for background and shading correction of optical microscopy images. *Nat. Commun.* **8**, 14836 (2017).

97. N. Jaccard, L. D. Griffin, A. Keser, R. J. Macown, A. Super, F. S. Veraitch, N. Szita, Automated method for the rapid and precise estimation of adherent cell culture characteristics from phase contrast microscopy images. *Biotechnol. Bioeng.* **111**, 504–517 (2014).

98. Y. Riahi, J. D. Wikstrom, E. Bachar-Wikstrom, N. Polin, H. Zucker, M.-S. Lee, W. Quan, L. Haataja, M. Liu, P. Arvan, E. Cerasi, G. Leibowitz, Autophagy is a major regulator of beta cell insulin homeostasis. *Diabetologia* **59**, 1480–1491 (2016).

99. K. J. Livak, T. D. Schmittgen, Analysis of relative gene expression data using real-time quantitative PCR and the 2(-delta delta C(T)) method. *Methods* **25**, 402–408 (2001).

100. Y. L. Liang, M. Khoshouei, M. Radjainia, Y. Zhang, A. Glukhova, J. Tarrasch, D. M. Thal, S. G. B. Furness, G. Christopoulos, T. Coudrat, R. Danev, W. Baumeister, L. J. Miller, A. Christopoulos, B. K. Kobilka, D. Wootten, G. Skiniotis, P. M. Sexton, Phase-plate cryo-EM structure of a class B GPCR–G-protein complex. *Nature* **546**, 118–123 (2017).

101. R. Fernandez-Leiro, S. H. W. Scheres, A pipeline approach to single-particle processing in RELION. *Acta Crystallogr. D Struct. Biol.* **73**, 496–502 (2017).

102. T. Wagner, F. Merino, M. Stabrin, T. Moriya, C. Antoni, A. Apelbaum, P. Hagel, O. Sitsel, T. Raisch, D. Prumbaum, D. Quentin, D. Roderer, S. Tacke, B. Siebolds, E. Schubert, T. R.

Shaikh, P. Lill, C. Gatsogiannis, S. Raunser, SPHIRE-crYOLO is a fast and accurate fully automated particle picker for cryo-EM. *Commun. Biol.* **2**, 218 (2019).

103. A. Punjani, J. L. Rubinstein, D. J. Fleet, M. A. Brubaker, cryoSPARC: Algorithms for rapid unsupervised cryo-EM structure determination. *Nat. Methods* **14**, 290–296 (2017).

104. T. I. Croll, ISOLDE: A physically realistic environment for model building into low-resolution electron-density maps. *Acta Crystallogr. D Struct. Biol.* **74**, 519–530 (2018).

105. E. F. Pettersen, T. D. Goddard, C. C. Huang, G. S. Couch, D. M. Greenblatt, E. C. Meng, T. E. Ferrin, UCSF Chimera—A visualization system for exploratory research and analysis. *J. Comput. Chem.* **25**, 1605–1612 (2004).

106. D. Liebschner, P. V. Afonine, M. L. Baker, G. Bunkoczi, V. B. Chen, T. I. Croll, B. Hintze, L. W. Hung, S. Jain, A. J. McCoy, N. W. Moriarty, R. D. Oeffner, B. K. Poon, M. G. Prisant, R. J. Read, J. S. Richardson, D. C. Richardson, M. D. Sammito, O. V. Sobolev, D. H. Stockwell, T. C. Terwilliger, A. G. Urzhumtsev, L. L. Videau, C. J. Williams, P. D. Adams, Macromolecular structure determination using x-rays, neutrons and electrons: Recent developments in Phenix. *Acta Crystallogr. D Struct. Biol.* **75**, 861–877 (2019).

107. M. A. Lomize, A. L. Lomize, I. D. Pogozheva, H. I. Mosberg, OPM: Orientations of proteins in membranes database. *Bioinformatics* **22**, 623–625 (2006).

108. J. Huang, A. D. MacKerell Jr., CHARMM36 all-atom additive protein force field: Validation based on comparison to NMR data. *J. Comput. Chem.* **34**, 2135–2145 (2013).

109. S. Doerr, M. J. Harvey, F. Noé, G. De Fabritiis, HTMD: High-throughput molecular dynamics for molecular discovery. *J. Chem. Theory Comput.* **12**, 1845–1852 (2016).

110. T. J. Dolinsky, J. E. Nielsen, J. A. McCammon, N. A. Baker, PDB2PQR: An automated pipeline for the setup of Poisson–Boltzmann electrostatics calculations. *Nucleic Acids Res.* **32**, W665–W667 (2004).

111. M. H. M. Olsson, C. R. SØndergaard, M. Rostkowski, J. H. Jensen, PROPKA3: Consistent treatment of internal and surface residues in empirical p K a predictions. *J. Chem. Theory Comput.* **7**, 525–537 (2011).

112. B. Sommer, Membrane packing problems: A short review on computational membrane modeling methods and tools. *Comput. Struct. Biotechnol. J.* **5**, e201302014 (2013).

113. W. L. Jorgensen, J. Chandrasekhar, J. D. Madura, R. W. Impey, M. L. Klein, Comparison of simple potential functions for simulating liquid water. *J. Chem. Phys.* **79**, 926–935 (1983).

114. M. J. Harvey, G. Giupponi, G. De Fabritiis, ACEMD: Accelerating biomolecular dynamics in the microsecond time scale. *J. Chem. Theory Comput.* **5**, 1632–1639 (2009).

115. H. J. C. Berendsen, J. P. M. Postma, W. F. Van Gunsteren, A. Dinola, J. R. Haak, Molecular dynamics with coupling to an external bath. *J. Chem. Phys.* **81**, 3684–3690 (1984).

116. R. J. Loncharich, B. R. Brooks, R. W. Pastor, Langevin dynamics of peptides: The frictional dependence of isomerization rates of N-acetylalanyl-N′-methylamide. *Biopolymers* **32**, 523–535 (1992).

117. V. Kräutler, W. F. Van Gunsteren, P. H. Hünenberger, A fast SHAKE algorithm to solve distance constraint equations for small molecules in molecular dynamics simulations. *J. Comput. Chem.* **22**, 501–508 (2001).

118. U. Essmann, L. Perera, M. L. Berkowitz, T. Darden, H. Lee, L. G. Pedersen, A smooth particle mesh Ewald method. *J. Chem. Phys.* **103**, 8577–8593 (1995).

119. A. Fiser, A. Sali, Modeller: Generation and refinement of homology-based protein structure models. *Methods Enzymol.* **374**, 461–491 (2003).

120. R. T. McGibbon, K. A. Beauchamp, M. P. Harrigan, C. Klein, J. M. Swails, C. X. Hernández, C. R. Schwantes, L.-P. Wang, T. J. Lane, V. S. Pande, MDTraj: A modern open library for the analysis of molecular dynamics trajectories. *Biophys. J.* **109**, 1528–1532 (2015).

121. W. Humphrey, A. Dalke, K. Schulten, VMD: Visual molecular dynamics. *J. Mol. Graph.* **14**, 33–38 (1996).

122. D. Wootten, J. Simms, L. J. Miller, A. Christopoulos, P. M. Sexton, Polar transmembrane interactions drive formation of ligand-specific and signal pathway-biased family B G protein-coupled receptor conformations. *Proc. Natl. Acad. Sci. U.S.A.* **110**, 5211–5216 (2013).

123. R. Irannejad, J. C. Tomshine, J. R. Tomshine, M. Chevalier, J. P. Mahoney, J. Steyaert, S. G. Rasmussen, R. K. Sunahara, H. El-Samad, B. Huang, M. von Zastrow, Conformational biosensors reveal GPCR signalling from endosomes. *Nature* **495**, 534–538 (2013).

124. V. Yu, F. Yong, A. Marta, S. Khadayate, A. Osakwe, S. Bhattacharya, S. S. Varghese, P. Chabosseau, S. M. Tabibi, K. Chen, E. Georgiadou, N. Parveen, M. Suleiman, Z. Stamoulis, L. Marselli, C. De Luca, M. Tesi, G. Ostinelli, L. Delgadillo-Silva, X. Wu, Y. Hatanaka, A. Montoya, J. Elliott, B. Patel, N. Demchenko, C. Whilding, P. Hajkova, P. Shliaha, H. Kramer, Y. Ali, P. Marchetti, R. Sladek, S. Dhawan, D. J. Withers, G. A. Rutter, S. J. Millership, Differential CpG methylation at Nnat in the early establishment of beta cell heterogeneity. *Diabetologia* **67**, 1079–1094 (2024).

125. S. Tyanova, T. Temu, J. Cox, The MaxQuant computational platform for mass spectrometry-based shotgun proteomics. *Nat. Protoc.* **11**, 2301–2319 (2016).

126. A. D. Shah, R. J. A. Goode, C. Huang, D. R. Powell, R. B. Schittenhelm, LFQ-analyst: An easy-to-use interactive web platform to analyze and visualize label-free proteomics data preprocessed with MaxQuant. *J. Proteome Res.* **19**, 204–211 (2020).
